# Genome-wide association study of 398,238 women unveils seven loci associated with high-grade serous ovarian cancer

**DOI:** 10.1038/s41525-025-00529-w

**Published:** 2025-11-20

**Authors:** Daniel R. Barnes, Jonathan P. Tyrer, Joe Dennis, Goska Leslie, Manjeet K. Bolla, Michael Lush, Amber M. Aeilts, Kristiina Aittomäki, Nadine Andrieu, Irene L. Andrulis, Hoda Anton-Culver, Adalgeir Arason, Banu K. Arun, Judith Balmaña, Elisa V. Bandera, Rosa B. Barkardottir, Lieke P. V. Berger, Amy Berrington de Gonzalez, Pascaline Berthet, Katarzyna Białkowska, Line Bjørge, Amie M. Blanco, Marinus J. Blok, Kristie A. Bobolis, Natalia V. Bogdanova, James D. Brenton, Henriett Butz, Saundra S. Buys, Maria A. Caligo, Ian Campbell, Carmen Castillo, Kathleen B. M. Claes, Sarah V. Colonna, Linda S. Cook, Mary B. Daly, Agnieszka Dansonka-Mieszkowska, Miguel de la Hoya, Anna deFazio, Allison DePersia, Yuan Chun Ding, Jennifer A. Doherty, Susan M. Domchek, Thilo Dörk, Zakaria Einbeigi, Christoph Engel, D. Gareth Evans, Lenka Foretova, Renée T. Fortner, Florentia Fostira, Maria Cristina Foti, Eitan Friedman, Megan N. Frone, Patricia A. Ganz, Aleksandra Gentry-Maharaj, Gord Glendon, Andrew K. Godwin, Anna González-Neira, Mark H. Greene, Jacek Gronwald, Aliana Guerrieri-Gonzaga, Ute Hamann, Thomas V. O. Hansen, Holly R. Harris, Jan Hauke, Florian Heitz, Frans B. L. Hogervorst, Maartje J. Hooning, John L. Hopper, Chad D. Huff, David G. Huntsman, Evgeny N. Imyanitov, Louise Izatt, Anna Jakubowska, Paul A. James, Ramunas Janavicius, Esther M. John, Siddhartha Kar, Beth Y. Karlan, Catherine J. Kennedy, Lambertus A.L.M. Kiemeney, Irene Konstantopoulou, Jolanta Kupryjanczyk, Yael Laitman, Ofer Lavie, Kate Lawrenson, Jenny Lester, Fabienne Lesueur, Carlos Lopez-Pleguezuelos, Phuong L. Mai, Siranoush Manoukian, Taymaa May, Iain A. McNeish, Usha Menon, Roger L. Milne, Francesmary Modugno, Jennifer M. Mongiovi, Marco Montagna, Kirsten B. Moysich, Susan L. Neuhausen, Finn C. Nielsen, Catherine Noguès, Edit Oláh, Olufunmilayo I. Olopade, Ana Osorio, Laura Papi, Harsh Pathak, Celeste L. Pearce, Inge S. Pedersen, Ana Peixoto, Tanja Pejovic, Pei-Chen Peng, Beth N. Peshkin, Paolo Peterlongo, C. Bethan Powell, Darya Prokofyeva, Miquel Angel Pujana, Paolo Radice, Muhammad U. Rashid, Gad Rennert, George Richenberg, Dale P. Sandler, Naoko Sasamoto, Veronica W. Setiawan, Priyanka Sharma, Weiva Sieh, Christian F. Singer, Katie Snape, Anna P. Sokolenko, Penny Soucy, Melissa C. Southey, Dominique Stoppa-Lyonnet, Rebecca Sutphen, Christian Sutter, Yen Y. Tan, Manuel R. Teixeira, Kathryn L. Terry, Liv Cecilie V. Thomsen, Marc Tischkowitz, Amanda E. Toland, Toon Van Gorp, Ana Vega, Digna R. Velez Edwards, Penelope M. Webb, Jeffrey N. Weitzel, Nicolas Wentzensen, Alice S. Whittemore, Stacey J. Winham, Anna H. Wu, Siddhartha Yadav, Yao Yu, Argyrios Ziogas, Andrew Berchuck, Fergus J. Couch, Ellen L. Goode, Marc T. Goodman, Alvaro N. Monteiro, Kenneth Offit, Susan J. Ramus, Harvey A. Risch, Joellen M. Schildkraut, Mads Thomassen, Jacques Simard, Douglas F. Easton, Michelle R. Jones, Georgia Chenevix-Trench, Simon A. Gayther, Antonis C. Antoniou, Paul D. P. Pharoah

**Affiliations:** 1 Centre for Cancer Genetic Epidemiology, Department of Public Health and Primary Care, University of Cambridge, Cambridge, UK; 2 Department of Internal Medicine, Division of Human Genetics, Ohio State University Comprehensive Cancer Center, The Ohio State University, Columbus, OH USA; 3 Department of Clinical Genetics, Helsinki University Hospital, University of Helsinki, Helsinki, Finland; 4 Inserm U1331, Paris, France; 5 Institut Curie, Paris, France; 6 Mines Paris, Paris, France; 7 PSL Research University, Paris, France; 8 Department of Molecular Genetics, University of Toronto, Toronto, ON Canada; 9 Lunenfeld-Tanenbaum Research Institute, Sinai Health System, Toronto, ON Canada; 10 Department of Medicine, University of California Irvine, Irvine, CA USA; 11 Department of Pathology, Landspitali - the National University Hospital of Iceland, Reykjavik, Iceland; 12 BMC (Biomedical Centre), Faculty of Medicine, University of Iceland, Reykjavik, Iceland; 13 Department of Breast Medical Oncology, University of Texas MD Anderson Cancer Center, Houston, TX USA; 14 Hereditary Cancer Genetics Group, Vall d’Hebron Institute of Oncology (VHIO), Barcelona, Spain; 15 Department of Medical Oncology, University Hospital of Vall d’Hebron, Barcelona, Spain; 16 Cancer Prevention and Control Program, Rutgers Cancer Institute of New Jersey, New Brunswick, NJ USA; 17 University of Groningen, University Medical Center Groningen, Department of Genetics, Groningen, The Netherlands; 18 Clinical Cancer Epidemiology, Institute of Cancer Research, London, UK; 19 Département de Biopathologie, Centre François Baclesse, Caen, France; 20 INSERM UMR1245 Cancer and Brain Genomics, Université de Rouen Normandie, Rouen, France; 21 Department of Genetics and Pathology, Pomeranian Medical University, Szczecin, Poland; 22 Department of Obstetrics and Gynecology, Haukeland University Hospital, Bergen, Norway; 23 Centre for Cancer Biomarkers CCBIO, Department of Clinical Science, University of Bergen, Bergen, Norway; 24 Cancer Genetics and Prevention Program, University of California San Francisco, San Francisco, CA USA; 25 Department of Clinical Genetics, Maastricht University Medical Center, Maastricht, The Netherlands; 26 City of Hope Clinical Cancer Genetics Community Research Network, Duarte, CA USA; 27 Department of Radiation Oncology, Hannover Medical School, Hannover, Germany; 28 Gynaecology Research Unit, Hannover Medical School, Hannover, Germany; 29 N.N. Alexandrov Research Institute of Oncology and Medical Radiology, Minsk, Belarus; 30 Cancer Research UK Cambridge Institute, University of Cambridge, Cambridge, UK; 31 Department of Molecular Genetics, National Institute of Oncology, Budapest, Hungary; 32 National Tumour Biology Laboratory, National Institute of Oncology, Budapest, Hungary; 33 Department of Oncology Biobank, National Institute of Oncology, Budapest, Hungary; 34 Department of Medicine, Huntsman Cancer Institute, University of Utah, Salt Lake City, UT USA; 35 SOD Genetica Molecolare, University Hospital, Pisa, Italy; 36 Cancer Genetics Laboratory, Peter MacCallum Cancer Centre, Melbourne, VIC Australia; 37 Hereditary Cancer Program, IDIBELL (Bellvitge Biomedical Research Institute), Catalan Institute of Oncology, Barcelona, Spain; 38 Center for Medical Genetics, Ghent University Hospital, Ghent, Belgium; 39 Department of Biomolecular Medicine, Ghent University, Ghent, Belgium; 40 Cancer Research Institute Ghent, Ghent, Belgium; 41 Department of Internal Medicine, Huntsman Cancer Institute, University of Utah, Salt Lake City, UT USA; 42 Department of Epidemiology, Colorado School of Public Health, University of Colorado, Aurora, CO USA; 43 Department of Clinical Genetics, Fox Chase Cancer Center, Philadelphia, PA USA; 44 Department of Pathology and Laboratory Medicine, Institute of Oncology and Maria Sklodowska-Curie Cancer Center, Warsaw, Poland; 45 Molecular Oncology Laboratory, CIBERONC, Hospital Clinico San Carlos, IdISSC (Instituto de Investigación Sanitaria del Hospital Clínico San Carlos), Madrid, Spain; 46 Centre for Cancer Research, The Westmead Institute for Medical Research, Sydney, NSW Australia; 47 Department of Gynaecological Oncology, Westmead Hospital, Sydney, NSW Australia; 48 Faculty of Medicine and Health, The University of Sydney, Sydney, NSW Australia; 49 The Daffodil Centre, The University of Sydney, a joint venture with Cancer Council NSW, Sydney, NSW Australia; 50 Center for Medical Genetics, Endeavor Health, Evanston, IL USA; 51 The University of Chicago Pritzker School of Medicine, Chicago, IL USA; 52 Department of Population Sciences, Beckman Research Institute of City of Hope, Duarte, CA USA; 53 Department of Population Health Sciences, Huntsman Cancer Institute, University of Utah, Salt Lake City, UT USA; 54 Basser Center for BRCA, Abramson Cancer Center, University of Pennsylvania, Philadelphia, PA USA; 55 Department of Oncology, Institute of Clinical Sciences, Sahlgrenska Academy, University of Gothenburg, Gothenburg, Sweden; 56 Institute for Medical Informatics, Statistics and Epidemiology, University of Leipzig, Leipzig, Germany; 57 Genomic Medicine, Division of Evolution and Genomic Sciences, The University of Manchester, Manchester Academic Health Science Centre, Manchester Universities Foundation Trust, St. Mary’s Hospital, Manchester, UK; 58 Genomic Medicine, North West Genomics hub, Manchester Academic Health Science Centre, Manchester Universities Foundation Trust, St. Mary’s Hospital, Manchester, UK; 59 Department of Cancer Epidemiology and Genetics, Masaryk Memorial Cancer Institute, Brno, Czech Republic; 60 Division of Cancer Epidemiology, German Cancer Research Center (DKFZ), Heidelberg, Germany; 61 Department of Research, Cancer Registry of Norway, Norwegian Institute of Public Health, Oslo, Norway; 62 Molecular Diagnostics Laboratory, INRASTES, National Centre for Scientific Research ‘Demokritos’, Athens, Greece; 63 Immunology and Molecular Oncology Unit, Veneto Institute of Oncology IOV - IRCCS, Padua, Italy; 64 Sackler Faculty of Medicine, Tel Aviv University, Ramat Aviv, Israel; 65 The Susanne Levy Gertner Oncogenetics Unit, Chaim Sheba Medical Center, Ramat Gan, Israel; 66 Assuta Medical Center, Tel-Aviv, Israel; 67 National Cancer Institute, Clinical Genetics Branch, Division of Cancer Epidemiology and Genetics, Bethesda, MD USA; 68 Schools of Medicine and Public Health, Division of Cancer Prevention & Control Research, Jonsson Comprehensive Cancer Centre, UCLA, Los Angeles, CA USA; 69 MRC Clinical Trials Unit at UCL, Institute of Clinical Trials & Methodology, University College London, London, UK; 70 Department of Women’s Cancer, Elizabeth Garrett Anderson Institute for Women’s Health, University College London, London, UK; 71 Department of Pathology and Laboratory Medicine, University of Kansas Medical Center, Kansas City, KS USA; 72 Human Genotyping Unit-CeGen, Spanish National Cancer Research Centre, Madrid, Spain; 73 Centre for Biomedical Network Research on Rare Diseases (CIBERER), Madrid, Spain; 74 Division of Cancer Prevention and Genetics, IEO, European Institute of Oncology IRCCS, Milan, Italy; 75 Molecular Genetics of Breast Cancer, German Cancer Research Center (DKFZ), Heidelberg, Germany; 76 Department of Clinical Genetics, Rigshospitalet, Copenhagen University Hospital, Copenhagen, Denmark; 77 Department of Clinical Medicine, Faculty of Health and Medical Sciences, University of Copenhagen, Copenhagen, Denmark; 78 Program in Epidemiology, Division of Public Health Sciences, Fred Hutchinson Cancer Center, Seattle, WA USA; 79 Department of Epidemiology, University of Washington, Seattle, WA USA; 80 Center for Familial Breast and Ovarian Cancer, Faculty of Medicine and University Hospital Cologne, University of Cologne, Cologne, Germany; 81 Center for Integrated Oncology (CIO), Faculty of Medicine and University Hospital Cologne, University of Cologne, Cologne, Germany; 82 Department of Gynecology and Gynecologic Oncology, Kliniken Essen-Mitte, Essen, Germany; 83 Family Cancer Clinic, The Netherlands Cancer Institute - Antoni van Leeuwenhoek hospital, Amsterdam, The Netherlands; 84 Department of Medical Oncology, Family Cancer Clinic, Erasmus MC Cancer Institute, Rotterdam, The Netherlands; 85 Centre for Epidemiology and Biostatistics, Melbourne School of Population and Global Health, University of Melbourne, Melbourne, VIC Australia; 86 Department of Epidemiology, University of Texas MD Anderson Cancer Center, Houston, TX USA; 87 British Columbia’s Ovarian Cancer Research (OVCARE) Program, BC Cancer, Vancouver General Hospital, and University of British Columbia, Vancouver, BC Canada; 88 Department of Pathology and Laboratory Medicine, University of British Columbia, Vancouver, BC Canada; 89 Department of Obstetrics and Gynecology, University of British Columbia, Vancouver, BC Canada; 90 Department of Molecular Oncology, BC Cancer Research Centre, Vancouver, BC Canada; 91 Department of Tumor Growth Biology, N.N. Petrov Institute of Oncology, St. Petersburg, Russia; 92 Clinical Genetics, Guy’s and St Thomas’ NHS Foundation Trust, London, UK; 93 Independent Laboratory of Molecular Biology and Genetic Diagnostics, Pomeranian Medical University, Szczecin, Poland; 94 Parkville Familial Cancer Centre, Peter MacCallum Cancer Center and the Royal Melbourne Hospital, Melbourne, VIC Australia; 95 Sir Peter MacCallum Department of Oncology, University of Melbourne, Melbourne, VIC Australia; 96 State Research Institute Centre for Innovative Medicine, Vilnius, Lithuania; 97 Hematology, Oncology and Transfusion Medicine Center, Oncogenetics Unit, Vilnius University Hospital Santaros Clinics, Vilnius, Lithuania; 98 Department of Human and Medical Genetics, Faculty of Medicine, Vilnius University, Vilnius, Lithuania; 99 Department of Epidemiology & Population Sciences, Stanford University School of Medicine, Stanford University, Stanford, CA USA; 100 Department of Medicine (Oncology), Stanford University School of Medicine, Stanford University, Stanford, CA USA; 101 Stanford Cancer Institute, Stanford University School of Medicine, Stanford University, Stanford, CA USA; 102 Centre for Cancer Genetic Epidemiology, Department of Oncology, University of Cambridge, Cambridge, UK; 103 David Geffen School of Medicine, Department of Obstetrics and Gynecology, University of California at Los Angeles, Los Angeles, CA USA; 104 Women’s Cancer Program at the Samuel Oschin Comprehensive Cancer Institute, Cedars-Sinai Medical Center, Los Angeles, CA USA; 105 Radboud Institute for Health Sciences, Radboud University Medical Center, Nijmegen, The Netherlands; 106 Technion-Israel Institute of Technology, Haifa, Israel; 107 Carmel Medical Center, Haifa, Israel; 108 Division of Gynecologic Oncology, Department of Obstetrics and Gynecology, Women’s Cancer Program at the Samuel Oschin Cancer Institute Cedars-Sinai Medical Center, Los Angeles, CA USA; 109 Fundación Pública Galega de Medicina Xenómica, Santiago de Compostela, Spain; 110 Instituto de Investigación Sanitaria de Santiago de Compostela (IDIS), Complejo Hospitalario Universitario de Santiago, SERGAS, Santiago de Compostela, Spain; 111 Escola de Doutoramento Internacional, Universidade de Santiago, Santiago de Compostela, Spain; 112 Magee-Womens Hospital, University of Pittsburgh School of Medicine, Pittsburgh, PA USA; 113 Unit of Medical Genetics, Department of Medical Oncology and Hematology, Fondazione IRCCS Istituto Nazionale dei Tumori di Milano, Milan, Italy; 114 Princess Margaret Cancer Center, Toronto, ON Canada; 115 Division of Cancer and Ovarian Cancer Action Research Centre, Department Surgery & Cancer, Imperial College London, London, UK; 116 Institute of Cancer Sciences, University of Glasgow, Glasgow, UK; 117 Cancer Epidemiology Division, Cancer Council Victoria, Melbourne, VIC Australia; 118 Precision Medicine, School of Clinical Sciences at Monash Health, Monash University, Clayton, VIC Australia; 119 Womens Cancer Research Center, Magee-Womens Research Institute and Hillman Cancer Center, Pittsburgh, PA USA; 120 Division of Gynecologic Oncology, Department of Obstetrics, Gynecology and Reproductive Sciences, University of Pittsburgh School of Medicine, Pittsburgh, PA USA; 121 Department of Obstetrics and Gynecology, Brigham and Women’s Hospital, Boston, MA USA; 122 Department of Epidemiology, Harvard T.H. Chan School of Public Health, Boston, MA USA; 123 Roswell Park Comprehensive Cancer Center, Buffalo, NY USA; 124 Center for Genomic Medicine, Rigshospitalet, Copenhagen University Hospital, Copenhagen, Denmark; 125 Département d’Anticipation et de Suivi des Cancers, Oncogénétique Clinique, Institut Paoli-Calmettes, Marseille, France; 126 Aix Marseille Université, INSERM, IRD, SESSTIM, Marseille, France; 127 Center for Clinical Cancer Genetics, The University of Chicago, Chicago, IL USA; 128 Health Research Institute-Fundación Jiménez Díaz University Hospital, Universidad Autónoma de Madrid (IIS-FJD, UAM), Madrid, Spain; 129 Department of Experimental and Clinical Biomedical Sciences ‘Mario Serio’, Medical Genetics Unit, University of Florence, Florence, Italy; 130 Department of Epidemiology, University of Michigan School of Public Health, Ann Arbor, MI USA; 131 Department of Preventive Medicine, Keck School of Medicine, University of Southern California Norris Comprehensive Cancer Center, Los Angeles, CA USA; 132 Molecular Diagnostics, Aalborg University Hospital, Aalborg, Denmark; 133 Clinical Cancer Research Center, Aalborg University Hospital, Aalborg, Denmark; 134 Department of Clinical Medicine, Aalborg University, Aalborg, Denmark; 135 Department of Laboratory Genetics, Portuguese Oncology Institute of Porto (IPO Porto) / Porto Comprehensive Cancer Center, Porto, Portugal; 136 Cancer Genetics Group, IPO Porto Research Center (CI-IPOP) / RISE@CI-IPOP (Health Research Network), Portuguese Oncology Institute of Porto (IPO Porto) / Porto Comprehensive Cancer Center, Porto, Portugal; 137 Department of Obstetrics & Gynecology, Providence Medical Center, Medford, OR USA; 138 Providence Cancer Center, Medford, OR USA; 139 Department of Computational Biomedicine, Cedars-Sinai Medical Center, Los Angeles, CA USA; 140 Lombardi Comprehensive Cancer Center, Georgetown University, Washington, DC USA; 141 Jess and Mildred Fisher Center for Hereditary Cancer and Clinical Genomics Research, Georgetown University, Washington, DC USA; 142 Laboratory of Hematology-Oncology, European Institute of Oncology IRCCS, Milan, Italy; 143 Ufa University of Science and Technology, Ufa, Russia; 144 ProCURE, IDIBELL (Bellvitge Biomedical Research Institute), Catalan Institute of Oncology, Barcelona, Spain; 145 ProCURE, IDIBGI (Girona Biomedical Research Institute), Catalan Institute of Oncology, Girona, Spain; 146 Unit of Predictive Medicine: Molecular Bases of Genetic Risk, Department of Experimental Oncology, Fondazione IRCCS Istituto Nazionale dei Tumori, Milan, Italy; 147 Department of Basic Sciences, Shaukat Khanum Memorial Cancer Hospital and Research Centre (SKMCH & RC), Lahore, Pakistan; 148 The Association for Promotion of Research in Precision Medicine, Haifa, Israel; 149 MRC Integrative Epidemiology Unit, University of Bristol, Bristol, UK; 150 Epidemiology Branch, National Institute of Environmental Health Sciences, NIH, Research Triangle Park, NC USA; 151 Department of Obstetrics, Gynecology and Reproductive Biology, Harvard Medical School, Boston, MA USA; 152 Department of Population and Public Health Sciences, University of Southern California, Los Angeles, CA USA; 153 Department of Internal Medicine, Division of Medical Oncology, University of Kansas Medical Center, Westwood, KS USA; 154 Department of Health Science and Policy, Icahn School of Medicine at Mount Sinai, New York, NY USA; 155 Department of Genetics and Genomic Sciences, Icahn School of Medicine at Mount Sinai, New York, NY USA; 156 Dept of OB/GYN and Comprehensive Cancer Center, Medical University of Vienna, Vienna, Austria; 157 Deptartment of Clinical Genetics, South West Thames Centre for Genomics, St George’s University Hospitals NHS Foundation Trust, London, UK; 158 Genomics Center, Centre Hospitalier Universitaire de Québec – Université Laval Research Center, Québec City, QC Canada; 159 Department of Clinical Pathology, Melbourne Medical School, University of Melbourne, Parkville, Victoria Australia; 160 Cancer Epidemiology Division, Cancer Council Victoria, East Melbourne, VIC Australia; 161 Genetics Department, Institut Curie, Paris, France; 162 INSERM U1339, Paris, France; 163 Université Paris Cité, Paris, France; 164 Health Informatics Institute, Morsani College of Medicine, University of South Florida, Tampa, FL USA; 165 Institute of Human Genetics, University Hospital Heidelberg, Heidelberg, Germany; 166 Department of Pathology and Molecular Immunology, School of Medicine and Biomedical Sciences (ICBAS), University of Porto, Porto, Portugal; 167 Obstetrics and Gynecology Epidemiology Center, Brigham and Women’s Hospital and Harvard Medical School, Boston, MA USA; 168 Medical Birth Registry of Norway, Norwegian Institute of Public Health, Oslo, Norway; 169 Department of Genomic Medicine, National Institute for Health Research Cambridge Biomedical Research Centre, University of Cambridge, Cambridge, UK; 170 Department of Cancer Biology and Genetics, Ohio State University Comprehensive Cancer Center, The Ohio State University, Columbus, OH USA; 171 Division of Gynecologic Oncology, University Hospital Leuven, Leuven, Belgium; 172 Leuven Cancer Institute, University of Leuven, Leuven, Belgium; 173 Centro de Investigación en Red de Enfermedades Raras (CIBERER), Madrid, Spain; 174 Department of Obstetrics and Gynecology, Vanderbilt University Medical Center, Nashville, TN USA; 175 Population Health Program, QIMR Berghofer Medical Research Institute, Brisbane, QID Australia; 176 Division of Precision Prevention, The University of Kansas Cancer Center, Kansas City, KS USA; 177 Division of Cancer Epidemiology and Genetics, National Cancer Institute, Bethesda, MD USA; 178 Department of Biomedical Data Science, Stanford University School of Medicine, Stanford, CA USA; 179 Department of Quantitative Health Sciences, Mayo Clinic College of Medicine, Rochester, MN USA; 180 Department of Population and Public Health Sciences, Keck School of Medicine, University of Southern California, Los Angeles, CA USA; 181 Department of Oncology, Mayo Clinic, Rochester, MN USA; 182 Department of Gynecologic Oncology, Duke University Hospital, Durham, NC USA; 183 Department of Laboratory Medicine and Pathology, Mayo Clinic, Rochester, MN USA; 184 Samuel Oschin Comprehensive Cancer Institute, Cancer Prevention and Genetics Program, Cedars-Sinai Medical Center, Los Angeles, CA USA; 185 Department of Cancer Epidemiology, Moffitt Cancer Center, Tampa, FL USA; 186 Clinical Genetics Research Lab, Department of Cancer Biology and Genetics, Memorial Sloan-Kettering Cancer Center, New York, NY USA; 187 Clinical Genetics Service, Department of Medicine, Memorial Sloan-Kettering Cancer Center, New York, NY USA; 188 AnaNeo Therapeutics, New York, NY USA; 189 School of Clinical Medicine, Faculty of Medicine and Health, University of NSW Sydney, Sydney, NSW Australia; 190 Adult Cancer Program, Lowy Cancer Research Centre, University of NSW Sydney, Sydney, NSW Australia; 191 Chronic Disease Epidemiology, Yale School of Medicine, New Haven, CT USA; 192 Department of Public Health Sciences, University of Virginia, Charlottesville, VA USA; 193 Department of Clinical Genetics, Odense University Hospital, Odense, Denmark; 194 Clinical Genome Center, Department of Clinical Research, University of Southern Denmark, Odense, Denmark; 195 Department of Molecular Medicine, Faculty of Medicine, Université Laval, Québec City, QC Canada; 196 Center for Bioinformatics and Functional Genomics, Cedars-Sinai Medical Center, Los Angeles, CA USA; 197 Department of Genetics and Computational Biology, QIMR Berghofer Medical Research Institute, Brisbane, QLD Australia

**Keywords:** Cancer genetics, Ovarian cancer, Cancer epidemiology, Cancer genetics, Ovarian cancer

## Abstract

Nineteen genomic regions have been associated with high-grade serous ovarian cancer (HGSOC). We meta-analyzed >22 million variants for 398,238 women from the Ovarian Cancer Association Consortium (OCAC), UK Biobank (UKBB) and Consortium of Investigators of Modifiers of *BRCA1*/*BRCA2* (CIMBA) to identify novel HGSOC susceptibility loci. Eight novel variants were associated with HGSOC risk. An interesting discovery biologically was *TP53* 3’-UTR SNP rs78378222-T’s association with HGSOC (per-T-allele relative risk (RR) = 1.44, 95% CI:1.28–1.62, *P* = 1.76 × 10^−9^). Polygenic scores (PGS) were developed using OCAC and CIMBA data and trained on FinnGen data. The optimal PGS included 64,518 variants and was associated with an odds ratio of 1.46 (95% CI:1.37–1.54) per standard deviation when validated in the UKBB. This study represents the largest HGSOC GWAS to date – demonstrating that improvements in imputation reference panels and increased sample sizes help to identify HGSOC associated variants that previously went undetected, ultimately improving PGS which can improve personalized HGSOC risk prediction.

## Introduction

Globally, epithelial ovarian cancer (EOC) is the seventh most common cancer diagnosed in women, with ~314,000 new cases diagnosed each year^1^. It is the most lethal gynecological cancer, responsible for ~207,000 deaths annually^1^. EOC is a collection of five major histotypes, namely high-grade serous (HGSOC), endometrioid, clear cell, low-grade serous (LGS) and mucinous, which are thought to have distinct etiology^2^. HGSOC is the most prevalent accounting for 60–70% of EOC diagnoses^2,3^, and accounting for most EOCs diagnosed in *BRCA1* and *BRCA2* pathogenic variant (PV) carriers^4–11^. Furthermore, HGSOC accounts for the majority of EOC mortality^12,13^.

To date, 40 genomic regions associated with EOC have been identified through genome-wide association studies (GWAS)^14–28^. For 19 of these regions, HGSOC is the most strongly associated histotype^14–22,26–28^. These studies have relied on imputation efforts that used the 1000 Genomes Project^29^ and Haplotype Reference Consortium^30^ reference panels, yielding up to ~11 million well-imputed genetic variants. The Trans-Omics for Precision Medicine (TOPMed) reference panel^31^ and imputation server^32^ have recently become publicly available. The TOPMed panel consists of ~308 million variants, yielding greater genomic coverage than previously available reference panels, with the added benefit of containing many more low-frequency and rare variants. This prompted us to re-impute genetic variant data from the population-based Ovarian Cancer Association Consortium (OCAC)^33^, and *BRCA1*/*2* carriers from the Consortium of Investigators of Modifiers of *BRCA1* and *BRCA2* (CIMBA)^34,35^ to assess whether the larger coverage of the genome from the TOPMed reference panel leads to detection of novel loci associated with HGSOC risk. We additionally made use of the UK Biobank (UKBB)^36,37^ to boost the sample size and power to detect associations. We combined these data with summary statistics from FinnGen^38,39^ and BioBank Japan^40,41^ to develop and validate polygenic models (PGM) and scores (PGS) for non-mucinous OC.

## Results

The genome-wide association analyses for HGSOC were based on up to 398,238 women from OCAC (*N* = 120,248, 30.2%), UKBB (*N* = 245,377, 61.6%) and CIMBA (*N* = 32,613, 8.2%) (Table 1, Supplementary Tables 1, 2). A total of 19,883 (5.0%) women were classified as being diagnosed with HGSOC. The mean (standard deviation, SD) age at diagnosis for women in OCAC and UKBB were 60.2 (10.9) years and 63.5 (10.0) years, respectively. The mean (SD) censoring ages for *BRCA1* and *BRCA2* PV carriers were 43.7 (SD = 12.0) years and 46.2 (SD = 12.9) years, respectively.

**Table 1:** Summary of European ancestry samples and imputed variants used in individual variant association analyses

Population	Study/consortium	Study design	Genotyping array	Imputation panel	*N* controls/unaffected^a^	*N* cases/affected^b^	*N* variants^c^
Population-based	OCAC	Case-control	GWAS panels^d^	TOPMed	9071	1745	142,163,775
			iCOGS	TOPMed	28,706	2661	142,163,775
			OncoArray	TOPMed	67,110	10,955	142,163,775
	UKBB	Nested case-control	UK BiLEVE Axiom Array and UK Biobank Axiom Array	UK10K and HRC	244,529	848	59,918,121
*BRCA1*/*2* carriers	CIMBA	Retrospective cohort	iCOGS	TOPMed	3947	632	80,292,260
			OncoArray	TOPMed	24,992	3042	104,435,671

*OCAC* Ovarian Cancer Association Consortium, *UKBB* UK Biobank, *CIMBA* Consortium of Investigators of Modifiers of *BRCA1* and *BRCA2*.

^a^Number of controls for population-based studies. UKBB analyses considered prevalent and incident cases to maximise number of cases. Number of carriers censored as unaffected for *BRCA1*/*2* carriers.

^b^Number of EOC cases for population-based studies. Number of carriers censored as affected with EOC for *BRCA1*/*2* carriers.

^c^Number of genotyped and well-imputed (imputation accuracy *r*^2^ > 0.30) genetic variants available for statistical analyses.

^d^The five GWAS panels were from the UK GWAS, US GWAS, Poland GWAS, Mayo Clinic Ovarian Cancer Study GWAS and the Brigham and Women’s Hospital GWAS.

### Re-examining previously identified associations with EOC

We looked up the associations for the lead variants previously reported as being associated with EOC in our newly generated results (Supplementary Table 3, Supplementary Figs. 1–41). Most lead variants previously reported to be associated specifically with HGSOC risk replicated in the present meta-analysis of OCAC, UKBB and *BRCA1/2* carriers at the significance threshold *P* < 5 × 10^−8^. Exceptions were chr2:111138666 (rs17041869)^26^, chr2:113216387 (rs895412), chr11: 62126500 (rs7937840)^26^ and chr22:28538325 (rs6005807)^22^ (Supplementary Table 3). However, the chr2:110525257..111658369 and chr2:112716387..113716387 regions contained other variants that were associated at the genome-wide significance level in the present analysis, whilst the chr11:61626500..62626500 and chr22:28038325..29038325 regions did not contain any variants associated with HGSOC at the genome-wide significance level. It should be noted, however, that the chr2:111138666 (rs17041869) and chr11:62126500 (rs7937840) variants were identified through a cross-cancer (breast, ovarian, and prostate) GWAS^26^ and were not specifically identified as HGSOC associated variants.

### Novel loci associated with HGSOC

Associations with a total of 5786 variants from 44 loci were significant at *P* < 5 × 10^−8^. We excluded 5778 variants at 37 loci from further consideration, as they were either near known associated regions (Supplementary Table 3), were not conditionally independent of the lead variant in a nearby known region, or were likely statistical artifacts arising from strata specific effects.

**Fig. 1:**
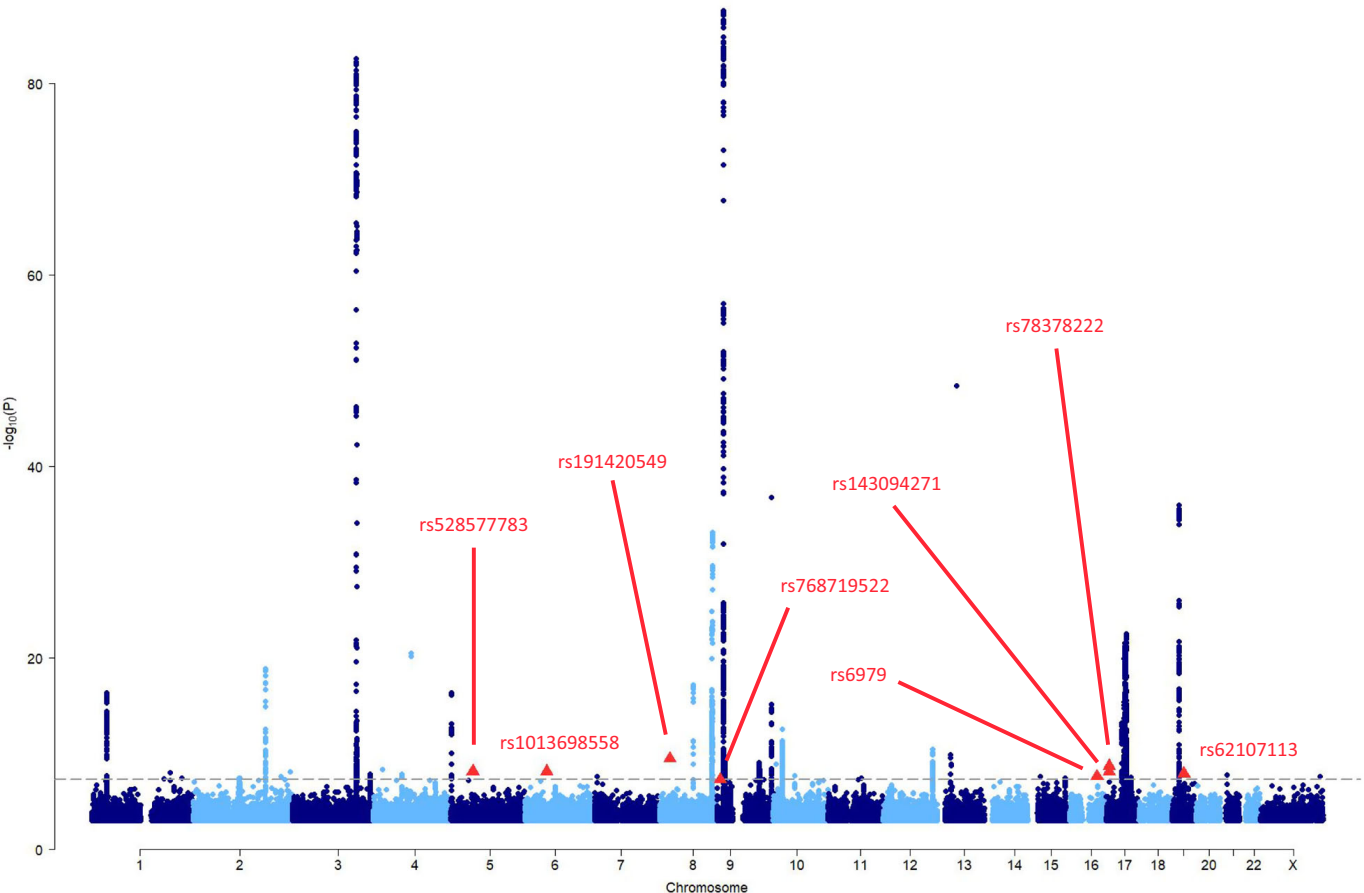
Manhattan plot showing the associations with HGSOC from the meta-analysis of OCAC, UKBB and CIMBA summary association data. The dashed line is the genome-wide statistical significance level (*P* = 5 × 10^−8^). The eight independent genome-wide statistically significant variants at seven novel loci are shown as red triangles.

There were eight variants associated (*P* < 5 × 10^−8^) with HGSOC, at 5q11, 6p12, 8p21, 9p24-23, 16q22, 17p13 and 19q12 (Table 2, Fig. 1, Supplementary Tables 4, 5).

**Table 2:** Eight independent genetic variants at seven loci associated with high-grade serous epithelial ovarian cancer from the OCAC, UK Biobank and *BRCA1/2* carrier meta-analysis

						Sample size^d^				Bayes false discovery probability	
Locus	Nearest gene (distance, kb)	SNP	Position^a^	Alleles^b^	Frequency^c^	Controls/unaffected	Cases/affected	RR (95% CI)^e^	*P*^f^	BFDP_3_^g^	BFDP_4_^g^
5q11.2	*FST* (24.693)	rs528577783	53511827	C/G	0.00120	375,400	18,882	5.15 (2.96–8.96)	6.60 × 10^−9^	0.33	3.21
6p12.1	*GCLC* (9.681)	rs1013698558	53554782	A/T	0.00148	133,826	19,035	2.35 (1.76–3.14)	6.86 × 10^−9^	0.06	0.62
8p21.2	*CDCA2* (156.65)	rs540569242	25522083	G/A	0.00014	375,839	19,254	25.60 (9.32–70.31)	3.15 × 10^−10^	3.17	24.70
9p24.1-p23	*PTPRD* (intronic)	rs768719522	8333517	C/T	0.00012	377,247	19,285	10.04 (4.38–22.99)	4.92 × 10^−8^	11.30	56.00
16q22.1	*ACD* (intronic)	rs6979	67657765	A/G	0.47145	378,355	19,883	1.07 (1.04–1.09)	2.30 × 10^−8^	0.12	1.19
17p13.1	*TNFSF12-TNFSF13* (intronic) *TNFSF13* (intronic)	rs143094271	7559785	A/G	0.97883	378,355	19,883	1.28 (1.18–1.39)	7.61 × 10^−9^	0.12	1.19
	*TP53* (3’ UTR)	rs78378222	7668434	G/T	0.98786	378,355	19,883	1.44 (1.28–1.62)	1.76 × 10^−9^	0.42	4.04
19q12	*CCNE1* (14.855)	rs62107113	29797136	G/A	0.22147	378,355	19,883	1.08 (1.05–1.11)	1.22 × 10^−8^	0.37	3.56

Supplementary Table 4 presents associations separately by OCAC, UK Biobank and *BRCA1*/*2* carriers.

*CI* confidence interval.

^a^Positions are Genome Reference Consortium Human Build 38 (GRCh38/hg38).

^b^Alleles = other/effect alleles.

^c^Frequency = frequency of the effect allele in the combined sample of OCAC and UK Biobank controls, and unaffected *BRCA1*/*2* carriers.

^d^Controls/unaffected = number of population-based controls from OCAC and UKBB, and the number of unaffected *BRCA1*/*2* carriers. Cases/affected = number of population-based EOC cases from OCAC and UKBB, and the number of affected *BRCA1*/*2* carriers.

^e^RR = relative risk from the OCAC, UK Biobank and *BRCA1/2* carrier meta-analysis, combining the odds ratio from the population-based data with the hazard ratio from the *BRCA1* and *BRCA2* carriers.

^f^*P*-value for association from the OCAC, UK Biobank and *BRCA1/2* carrier meta-analysis.

^g^BFDP_M_ = Bayes false discovery probability under model assuming a prior probability that 1 in 10^M^ variants are truly associated (%).

The associations at 5q11 (rs528577783-G; RR = 5.15, 95% CI:2.96–8.96), 6p12 (rs1013698558-T; RR = 2.35, 95% CI:1.76–3.14), 8p21 (rs540569242-A; RR = 25.60, 95% CI:9.32–70.31), and 9p24-p23 (rs768719522-T; RR = 10.04, 95% CI:4.38-22.99) were all single rare variants (MAF ≤ 0.15%) associated with large HGSOC effects. The single SNPs associated with HGSOC at 16q22 (rs6979-G; RR = 1.07, 95% CI:1.04-1.09) and 19q12 (rs62107113-A; RR = 1.08, 95% CI:1.05–1.11) were common and conferred modest effects on HGSOC risk. There were two moderately correlated (TOPMed European^42^ *r*^2^ = 0.46, D’ = 0.89) low-frequency (MAF: 1.2% and 2.1%) variants at the 17p13 locus. The *TNFS13*/*TNFSF12*-*TNFSF13* intronic variant rs143094271-G was associated with a per-allele RR = 1.28 (95% CI:1.18–1.39, *P* = 7.61 × 10^−9^); and rs78378222-T, a *TP53* 3’-UTR variant, with a per-allele RR = 1.44 (95% CI:1.28–1.62, *P* = 1.76 × 10^−9^). The association effect size estimates at 16q22, 19q12 and 17p13 were consistent between OCAC/UKBB and *BRCA1/2* PV carriers.

Bayesian false-discovery probabilities (BFDPs) indicated that six of these associations are likely to be true, although two rare variants, rs540569242 and rs768719522, had noticeably larger BFDPs (Table 2). Under a model assuming 1:1,000 truly associated variants, the BFDPs were 3.2% for rs540569242 and 11% for rs768719522. The other variants all had BFDP ≤ 0.42%.

### Credible causal variants (CCVs)

We defined 52 CCVs across the seven novel regions (Supplementary Table 6, Supplementary Figs. 42–48). Four regions (5q11, 6p12, 8p21, 9p24-23) had only the lead variant as a CCV, whilst the 16q22 (*N* = 5), 17p13 (*N* = 3) and 19q12 (*N* = 40) loci had several CCVs.

### Association of the PGS with HGSOC

Of the 1102 PGMs developed using OCAC and CIMBA data, the PGM that performed best in the FinnGen data comprised of 64,518 variants (Supplementary Data 1), denoted PGS_64518_. In the UKBB validation, the OR per SD of PGS_64518_ was estimated to be 1.46 (95% CI:1.37–1.54), with discriminatory ability of AUROC = 0.607 (95% CI:0.590–0.623) (Table 3). The association of PGS_64518_ was strongly attenuated in the BBJ validation (East Asian ancestry women), where the OR per SD was 1.12 (95% CI:1.05–1.20).

**Table 3:** Associations of polygenic scores (PGS) in UK Biobank and BioBank Japan

Nvariants	Selection^a^	Threshold^b^	UKBB associations^c^		BioBank Japan associations^d^	*BRCA1* carrier lifetime risks at PGS percentiles (%)^e^			*BRCA2* carrier lifetime risks at PGS percentiles (%)^e^		
			AUC	OR (95% CI)	OR (95% CI)	5^th^ centile	Median	95^th^ centile	5^th^ centile	Median	95^th^ centile
64,518	All	0.02	0.607	1.46 (1.37–1.54)	1.12 (1.05–1.20)	25.9	42.8	64.7	9.3	16.7	28.9
5957^f^	All	0.02	0.603	1.45 (1.37–1.54)	1.14 (1.07–1.22)	26.2	42.8	64.4	9.4	16.7	28.7
3448^f^	All	0.01	0.604	1.45 (1.37–1.54)	1.14 (1.06–1.22)	26.2	42.8	64.3	9.5	16.7	28.7
400^f^	Filter	0.0005	0.603	1.43 (1.35–1.52)	1.13 (1.06–1.21)	26.7	42.9	63.6	9.7	16.8	28.3
36^g^	All	Ad hoc	0.595	1.40 (1.32–1.48)	1.13 (1.06–1.21)	27.7	43.0	62.3	10.1	16.9	27.5

*PGS* polygenic score, *UKBB* UK Biobank, *AUC* area under the receiver operator classification (ROC) curve, *OR* odds ratio per standard deviation, *CI* confidence interval.

^a^Set of variants considered for inclusion. “All” means that all imputed variants were considered. “Filter” means only non-indel variants genotyped on the OncoArray were considered.

^b^The *P*-value:LD-r^2^ ratio used to select variants for inclusion in the PGM.

^c^Association of the PGS estimated in 245,611 European ancestry women from UK Biobank (244,529 controls, 1082 cases).

^d^Association of the PGS estimated in 61,457 East Asian ancestry women from BioBank Japan (60,614 controls, 843 cases).

^e^Lifetime risks of developing ovarian cancer (at age 80 years) at the 5^th^, 50^th^ (median), and 95^th^ percentiles of the PGS distribution for a *BRCA1*/*2* pathogenic variant carrier. Calculations assume ovarian cancer incidence rates for *BRCA1*/*2* pathogenic variant carriers from Kuchenbaecker et al.^130^.

^f^Subset of the most predictive variants from the 64,518 variant PGS that were well genotyped on the OncoArray.

^g^36 variant PGM developed by Dareng et al.^43^.

When restricting the PGS to include only genotyped variants from the 64,518 genotyped and imputed variants, which may make their implementation easier, PGS with 5957 (all genotyped variants from the 64,518) and 400 variants had similar performance characteristics. Relative to the PGS_64518_, a PGS considering the 400 most strongly associated genotyped variants, denoted PGS_400_, resulted in a small decrease in the AUROC to 0.603, and a marginally attenuated OR per SD (OR = 1.43, 95% CI:1.35–1.52) in the UKBB.

### Predicted absolute risks for the general population and BRCA1/2 pathogenic variant carriers

**Fig. 2:**
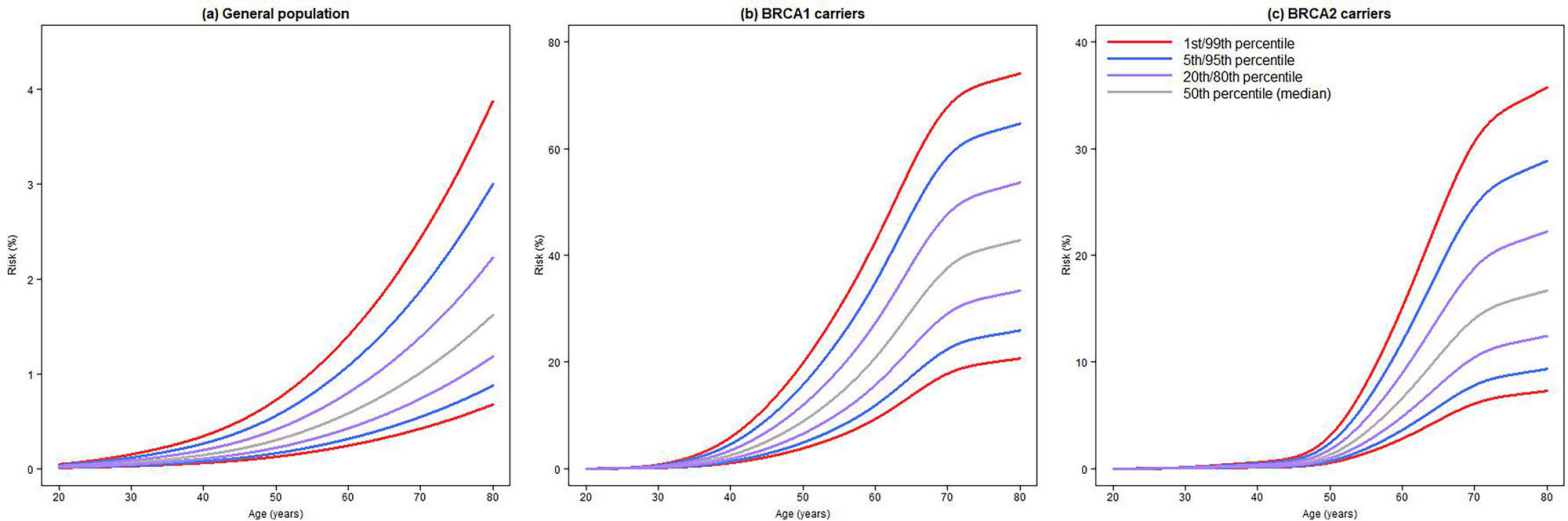
Predicted cumulative risks of developing EOC by PGS percentiles. Predicted cumulative risks of developing EOC based on the PGS_64518_ at various percentiles of the PGS distribution for: **a** the general population (0.7% for 1^st^ percentile to 3.9% for the 99^th^ percentile), **b** *BRCA1* PV carriers (20.7% for 1^st^ percentile to 74.1% for the 99^th^ percentile), and **c** *BRCA2* PV carriers (7.3% for 1^st^ percentile to 35.7% for the 99^th^ percentile).

Absolute lifetime risks of developing EOC by age 80 years for a woman from the general population were calculated to be 0.9%, 1.6% and 3.0% at the 5^th^, 50^th^ and 95^th^ percentiles of the PGS_64518_ (Table 3, Fig. 2). The absolute lifetime risks (at age 80 years) for *BRCA1* PV carriers were predicted to be 25.9%, 42.8% and 64.7% at the 5^th^, 50^th^ and 95^th^ percentiles of the PGS_64518_ distribution, respectively (Table 3, Fig. 2). The corresponding risks for PGS_64518_ for *BRCA2* PV carriers at the same PGS percentiles were predicted to be 9.3%, 16.7% and 28.9%, respectively (Table 3, Fig. 2). The range of predicted percentile specific risks for the previously published 36 variant PGS^43^ was narrower, with risks for the same percentiles of 27.7%, 43.0% and 62.3% for *BRCA1* PV carriers, respectively, and 10.1%, 16.9% and 27.5% for *BRCA2* PV carriers, respectively. The PGS_400_ yielded absolute risks which were approximately at the midpoint of the 36 and 64,518 variant PGS absolute risks (*BRCA1* PV carriers: 26.7%, 42.9% and 63.6% at the 5^th^, 50^th^ and 95^th^ percentiles, respectively; *BRCA2* PV carriers: 9.7%, 16.8% and 28.3% at the 5^th^, 50^th^ and 95^th^ percentiles, respectively).

There was a total reclassification of *BRCA2* PV carriers at the 10% risk threshold when considering PGS_400_ and PGS_64518_ of 4.8% and 5.4%, respectively, compared to the 36 variant PGS (Supplementary Table 7).

## Discussion

We conducted the largest GWAS to date for HGSOC, both in terms of the sample size and the number of genetic variants assessed. To do this, we made use of large international consortia (OCAC and CIMBA), and harnessed data from the UKBB to bolster the sample size. We also utilized recent advances in imputation reference panels, namely the TOPMed panel, which allowed us to investigate the largest number of genetic variants to date. We identified eight independent variants at seven loci to be genome-wide statistically significant for association with HGSOC risk, including four rare variants (MAF < 1%) and two low-frequency polymorphisms (MAF 1–5%), demonstrating that GWAS with greater genomic coverage for imputation can contribute to identify previously undiscovered rare variant associations. Based on these associations, we defined 52 CCVs that have the strongest statistical evidence for being the likely causal variant for each locus. We also developed a polygenic model that exhibited improved discriminatory ability compared to previously published models.

The four rare variants were associated with large effect sizes, with RR estimates ranging from 2.35 to 25.6. The large effect sizes seem implausible and may reflect a winner’s curse effect with true effect sizes actually being lower than these estimates^44^. Moreover, they may represent false positive associations, hence we estimated BFDPs^45^ for the eight novel variants to determine the likelihood of this. Six variants had low BFDPs, indicating they are likely true associations. However, 8p21 rs540569242 and 9p24-p23 rs768719522 had noticeably larger BFDPs and are more likely to represent false positive associations.

Variant rs78378222, in the *TP53* 3’-UTR, with the major T-allele (AF 98.8%) is associated with an increased risk of HGSOC. The same allele has been associated with an increased risk of triple-negative breast cancer^46^ with a per-allele relative risk of 1.45, similar to its association with HGSOC (RR = 1.44). In contrast, the minor (G) allele of rs78378222 is associated with increased risks of skin^47–51^, brain^51–55^ and prostate cancers^47,56^. One study also found rs78378222 to be associated with standing height, lean body mass and basal metabolic rate^51^. The minor allele has been shown to impede *TP53* 3’-end processing, resulting in downregulated p53 mRNA levels and protein levels, and decreased apoptosis^57^. Furthermore, germline and somatic variations in *TP53* are well established factors in cancer development and progression through its role as a tumor suppressor^58–61^ and the *TP53* 3’-UTR germline variant has been shown to interact with tumor *TP53* mutation status^62^. A previous study by the OCAC found five SNPs in the *TP53* region, within ±21 kb of rs78378222, to be associated with invasive EOC^63^. However, none of these SNPs are in LD with rs78378222.

rs528577783 is a rare intergenic variant between the *FST* and *NDUFS4* genes. *FST* encodes follistatin, a single-chain gonadal protein that specifically inhibits follicle-stimulating hormone release and is moderately expressed in human reproductive tissues (Supplementary Table 5)^64^. A linkage study identified *FST* as a candidate gene for polycystic ovary syndrome (PCOS)^65^. PCOS may be associated with an increased risk of borderline or postmenopausal ovarian cancer^66^, although a recent Mendelian Randomization study found that genetically predicted PCOS was associated with decreased risk of ovarian cancer^67^. A GWAS of African ancestry women found rs37792 near *FST* to be genome-wide statistically significantly associated with HGSOC in African ancestry women^68^.

The 6p12 variant rs1013698558, located ~9.7 kb from the *GCLC* gene, was moderately associated with HGSOC. A study found a synthetic lethality relationship between *GCLC* and *ARID1A*-deficient OC cells^69^, whilst another reports that *GCLC* inhibition causes apoptosis in *ARID1A*-deficient cancer cells^70^. *ARID1A* has been implicated as a tumor suppressor gene^71^. It may be that the association we find for rs1013698558 with HGSOC is a result of the interplay between *GCLC* and *ARID1A*. The 8p21 variant rs540569242 is 156 kb from *CDCA2* and was associated with HGSOC with the largest RR that we report. *CDCA2* is involved in the cell division cycle and response to DNA damage. One study found *CDCA2* expression is upregulated in ovarian tumor tissue compared with normal tissue^72^. This study also found that *CDCA2* and its 100 most co-expressed genes were primarily involved in cell cycle, oocyte meiosis, progesterone-mediated oocyte maturation, p53 signaling and pyruvate metabolism pathways. We found that the *PTPRD* intronic variant rs768719522 at the 9p24-p23 locus had a large association RR with HGSOC. *PTPRD* has been implicated as a tumor suppressor gene^73^. This gene encodes a protein from the protein tyrosine phosphatase (PTP) family – PTPs are signaling molecules regulating processes such as cell growth, cell differentiation, the mitotic cycle and oncogenic transformation^74^. The common *ACD* missense variant rs6979 at 16q22 conferred a small per-allele RR to HGSOC risk. This gene encodes telomere protein TPP1, which is involved in maintenance of telomere length and protecting telomere ends. In addition to the 17p13 *TP53* 3’-UTR variant association, there was another independent variant associated with HGSOC risk at this locus. The rs143094271 variant is intronic in *TNFSF13*/*TNFSF12*-*TNFSF13*. These genes are members of the tumor necrosis factor family, which are involved in various cellular processes, including survival, proliferation, differentiation, and apoptosis. rs143094271-G has been associated with a decreased risk of having uterine fibroids (OR = 0.70)^75^. Women with uterine fibroids have been found to be at increased risk of developing OC^76^. However, despite rs143094271-G being associated with women being less likely to have uterine fibroids, we found that rs143094271-G yielded an increased risk of developing HGSOC. The 19q12 common variant rs62107113 is located ~15 kb from the *CCNE1* gene and was associated with a modest increased risk of developing HGSOC. *CCNE1* encodes cyclin E1, which regulate cyclin-dependent kinases. Overexpression of *CCNE1* has been observed in genomically unstable tumors, notably HGSOC^77–81^ and triple-negative breast cancer^78,82–84^. Moreover, it has been shown that replication stress in cells overexpressing *CCNE1* is likely a consequence of replication initiation, ultimately resulting in DNA damage and genomic instability^85^. There is evidence indicating *CCNE1* amplification is mutually exclusive to BRCA inactivation^79^. *CCNE1* is an exciting novel therapeutic target, Gallo et al. developed an orally bioavailable PKMYT1 inhibitor that activated CDK1 in *CCNE1* overexpressed cells, promoting early mitosis in cells undergoing DNA synthesis^86^.

In addition to the 40 loci previously found to be associated with EOC^14–27^, we have identified genetic variants at a further seven loci associated with HGSOC, taking the number of loci associated with EOC to 47, 26 specifically with HGSOC. The previous known loci (52 variants at 40 loci) explained 8.5% of the polygenic variance of OC, assuming a total polygenic variance of 2.004 from Lee et al.^87^. The six variants that we report to be associated with HGSOC with low BFDP explain an additional 2.8%, making the total variance explained by GWAS identified variants to be 11.3%.

For previously identified variants, the estimated associations from general population data (combined OCAC and UKBB) ORs were broadly consistent with the estimated HRs for *BRCA1* and *BRCA2* PV carriers (Supplementary Table 3). Furthermore, testing for heterogeneity of effects resulted in a small number of variants exhibiting differences in effect sizes between population-based ORs and carrier HRs. None of the eight novel associations exhibited any heterogeneous effects between population-based ORs and *BRCA1*/*2* PV carrier HRs (Supplementary Table 4).

Using the S4 method, we developed a 64,518 variant PGM, whose PGS was associated with a per SD OR of 1.46 (95% CI:1.37–1.54) and had discriminatory ability of AUROC = 0.607 (95% CI:0.590–0.623). There was a small improvement over the previous best performing PGM developed by Dareng et al.^43^ (18,007 variants; OR per unit SD = 1.42; AUROC = 0.596) developed using similar methodology. The PGS_400_ could be more practical to implement in a clinical setting, since it contains fewer variants, all of which are known to successfully genotype from the OncoArray experiment, compared to the best performing PGS_64518_ which requires imputation. We found that the PGS_400_ had similar performance to the optimal PGS_64518_. This also suggests that most of the predictive ability of the PGS may derive from genotyped SNPs with the largest variance contributions.

When each of the PGS were tested in individuals of East Asian ancestry from BBJ, the PGS associations were strongly attenuated, each to a similar degree. For women of East Asian ancestry, the PGS consisting of genotyped variants with 5957 and 3448 variants performed marginally better, with a slightly larger OR per PGS SD. The observed attenuations for East Asian women compared with European women are likely a result of the PGM derivation data being strongly weighted towards Europeans, as the majority of OCAC and CIMBA samples came from this ancestry group.

We calculated lifetime risks of developing EOC by PGS percentiles for the optimal 64,518 variant PGS for *BRCA1*/*2* carriers. The risks ranged from 25.9% to 64.7% for *BRCA1* carriers, and 9.3% to 28.9% for *BRCA2* carriers, at the 5^th^ and 95^th^ percentiles, respectively. The range of risks for the 36 variant PGS that is currently implemented in the CanRisk ovarian cancer risk prediction algorithm^43,87^ were narrower than those for the PGS_64518_. The lifetime risks based on the PGS_400_ that used a subset of 400 reliably genotyped variants included in the PGS_64518_ at these percentiles sat approximately at the midpoints of the risks from the 64,518 and 36 variant PGS. We compared what risk reclassification (lower risk, <10%, or higher risk, ≥10% lifetime risk) would occur when using the PGS_64518_ or PGS_400_ versus the 36 variant PGS for *BRCA2* PV carriers. We were unable to assess reclassification for *BRCA1* carriers as the lifetime absolute risks at the lowest percentiles of the PGS distributions were always above 10%. We found that the PGS_64518_ and PGS_400_ led to total reclassification of risk groups of around 5% versus the 36 variant PGS. Most reclassification shifted women from lower risk (<10%) using the 36 variant PGS to higher risk (≥10%) using the alternative PGS_64518_ or PGS_400_. Taken together, these estimated lifetime risks and risk reclassifications will help to more accurately determine a carriers’ risk and inform clinical management of risk, such as the timing of risk reducing surgery or the initiation of cancer surveillance. In practice, PGS should be used in combination with pathogenic variants in ovarian cancer susceptibility genes and other risk lifestyle/hormonal factors in validated multifactorial cancer risk prediction models, such as CanRisk^87–89^, to improve comprehensive risk assessment. The clinical implementation of PGMs is mainly in the context of risk management for women with a family history of ovarian cancer. The CanRisk breast/ovarian cancer risk prediction model^87–89^ currently incorporates polygenic scores for both breast and ovarian cancer, together with lifestyle risk factors, family history and moderate/high penetrance risk alleles. The familial risk used in the model is the risk after exclusion of the contribution of polygenic risk as the polygenic risk explains ~11.3% of the excess familial risk. A key assumption that underlies the model is that the effects of the various risk factors are multiplicative (log-additive). There is a large body of evidence supporting this assumption^90–99^.

Strengths of this study include using the TOPMed imputation reference panel, enabling us to assess a larger number of low-frequency and rare variants than previous studies. A further strength was its power to detect low-frequency and rare variant associations, as well as previously unidentified common variant associations. This was facilitated by additional genotyped samples included in both OCAC and CIMBA and using population-based data from UKBB, resulting in the largest sample size analyzed for assessing genetic variant associations with HGSOC risk.

Limitations include the fact that the GWAS discovery data available were primarily of European ancestry; the associations of these variants are likely to differ for women of non-European ancestries, as they are likely to have different frequencies and LD patterns. A limitation of the PGM was that the derivation data differed from the GWAS discovery data presented here. Ideally, all the discovery GWAS data would have been used for PGM development. However, it was essential to validate the PGM on independent data, hence the OCAC and CIMBA data were used for development, whilst the UKBB data were reserved for validation. Lastly, the PGM training data (FinnGen) did not have specific histotypes available, meaning we were only able to consider overall EOC in the PGM training. As we were investigating non-mucinous OC, we would ideally have had specific EOC histotypes available at each stage of the PGM development, training and validation. However, given that HGSOC is the most prevalent EOC histotype, it is unlikely to have a major impact on the PGM hyperparameter fine-tuning.

Future research may aim to fine-map the novel loci identified here, to refine the candidate causal variants associated with HGSOC risk; and in-silico analyses may identify candidate target genes or pathways for further experimental studies^100^. Additionally, future research could aim to identify novel variants associated with other OC histotypes and to discover novel associations for other ancestries.

We have shown that improvements in imputation reference panels that have larger genomic coverage and increased sample sizes can assist in identification of novel HGSOC associated variants that previously went undetected, either from absence from genotyping or imputation reference panels, or from lack of power to detect associations. Furthermore, these associations can be used to develop PGM that outperform previous best efforts that can be incorporated into cancer risk prediction algorithms to improve personalized risk prediction for HGSOC.

## Methods

### Study samples

OCAC participants were enrolled in 65 studies from 16 countries and a large European multinational nested case-control study (Supplementary Table 1). OCAC individual participant data were used for GWAS discovery analyses and developing polygenic models (PGMs).

CIMBA study participants were enrolled in 64 studies from 28 countries (Supplementary Table 2). Eligibility was restricted to women aged at least 18 years at the time of recruitment who carried a PV in either *BRCA1* or *BRCA2*. Data collected included year of birth, PV description, age at recruitment, age at last follow-up, and age at breast and ovarian cancer (invasive, fallopian tube and peritoneal) diagnoses, and age or date of prophylactic surgeries (bilateral mastectomy and bilateral oophorectomy). Most participants were recruited through cancer genetics clinics and enrolled in regional/national research studies. CIMBA individual participant data were used in the GWAS discovery and in PGM development.

The UK Biobank (UKBB) is a large-scale biomedical research resource, with detailed genetic and health data on half a million UK participants^36,37^. For the purposes of these analyses, data from 245,377 female participants of European ancestry were used. UKBB individual participant data were used in the GWAS discovery analyses and to independently validate PGS.

FinnGen is a large collection of newly recruited and legacy samples from Finnish biobanks, research institutes, universities, university hospitals, international pharmaceutical partners, the Finnish Blood Service, the Finnish Biobank Cooperative, and the Finnish Institute for Health and Welfare, utilizing Finnish longitudinal health register data collected on every resident of Finland since 1969^38,39^. FinnGen summary statistics (data freeze 8) based on 150,658 women (149,394 controls, 1264 EOC cases of any histotype) were used to train PGM hyperparameters.

BioBank Japan (BBJ) is a large biobank resource containing clinical and genetic data on over 300,000 participants^40,41^. BBJ summary statistics based on 61,457 women (60,614 controls, 843 EOC cases) were used for assessing PGS associations for women of East Asian ancestry^101^.

### Genotyping and re-imputation using the TOPMed reference panel

Genotyping of OCAC and CIMBA samples were performed on one of two custom single nucleotide polymorphism (SNP) genotyping arrays, the iCOGS^23,102^ array or OncoArray^22,103,104^. The iCOGS array included ~210,000 SNPs that were selected for previous evidence of association with breast, ovarian and prostate cancer. The OncoArray is a custom genotyping chip consisting of ~533,000 SNPs, approximately half of which is a GWAS backbone that tags common SNPs. A standard quality control (QC) process was applied, including assessment of SNP call rate, allele frequency, genotyping intensity clustering, Hardy-Weinberg equilibrium, and SNP concordance from duplicated samples^104^. OCAC had additional samples genotyped on GWAS arrays^14–16^. These data were imputed to the TOPMed reference panel (version R2 on GRCh38, with 97,256 samples)^31^ using the Michigan Imputation Server^32,105^. Phasing was performed with Eagle2^106^ and imputation with Minimac^107,108^. Prior to imputation, variants were excluded from imputation genotype files if they: (1) were not in Hardy-Weinberg equilibrium (*P* < 1 × 10^−7^ in controls, or *P* < 1 × 10^−12^ in cases); (2) had poor cluster plots; or (3) had a call rate <95% (common variants), or a call rate <98% (rare variants, MAF < 1%). We used https://www.well.ox.ac.uk/~wrayner/tools/ script HRC-1000G-check-bim-v4.3.0.pl to remove variants not on the TOPMed reference panel or align them to the correct strand. This tool excluded variants where the genotyped frequency differed from the panel by more than 0.20. Samples were randomized into batches of <25,000 to meet the maximum sample requirement of the imputation server^31,32,105^. The same list of variants was included for each genotype batch. Details of the UKBB genotyping and imputation to a combined UK10K^109,110^ and HRC^30^ reference panel have been described elsewhere^36,37^. The OCAC, CIMBA and UKBB analyses were based on 142 million, 104 million and 60 million well-imputed (imputation *r*^2^ > 0.30) variants, respectively (Table 1). Downstream meta-analyses were restricted to variants that had minor allele counts (MACs) of MAC > 5 and did not have heterogeneous effects (Cochran Q-statistic, *P*_het_ > 1 × 10^−8^) in the meta-analysis of OCAC studies. Variants were aligned to the Genome Reference Consortium Human Build 38 (GRCh38/hg38).

Analyses including FinnGen and BBJ data made use of summary statistic data only. Details of FinnGen and BBJ genotyping and imputation have been described elsewhere^38–41,101^.

### Statistical analyses of OCAC and UKBB data

**Fig. 3:**
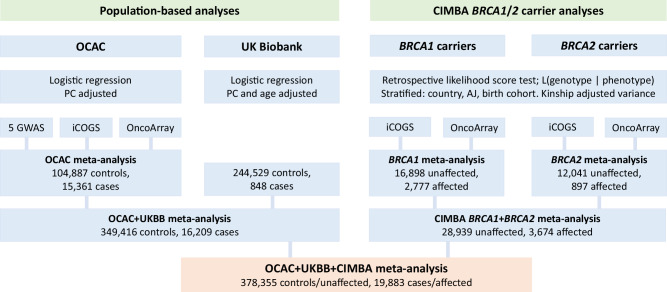
Schema describing the analytical process for the GWAS analyses from OCAC, UKBB and CIMBA, and subsequent meta-analyses.

We examined the associations between genotypes and HGSOC risk in the OCAC data using logistic regression (using custom software). Analyses were conducted separately for OncoArray, iCOGS, and five GWAS datasets^14–16,111^ and were combined by fixed-effects inverse-variance weighted meta-analysis (Fig. 3). We included project-specific principal components (PCs) as covariates in the model with the number of PCs based on the inflection point observed in a scree plot (Supplementary Fig. 49). PCs for OncoArray data were calculated using 33,661 uncorrelated (*r*^2^ < 0.10) common (MAF > 0.05) variants. Calculations were performed using a custom program (PCAcalc), available at https://github.com/CCGE-Cambridge/OCAC_CIMBA_HGSOC. Details of PC calculations for the other genotype data has been previously described elsewhere^19,23^. All women were of European ancestry and unrelated, determined using genetic data^22,103^. Ancestry was evaluated using the FastPop software^112^. Women with >80% European ancestry were retained for statistical analyses. Relatedness was checked by genetic concordance statistics. This process has been described previously^22^. Briefly, women with concordance statistics between 0.74 and 0.86 were considered to be related (values > 0.86 considered duplicates). In instances of case-control pairs, the case was retained for statistical analyses and the control was excluded. For instances of case-case and control-control pairs, the sample with the lower call rate was excluded.

The UKBB analysis was restricted to women with European ancestry, selected based on their PCs. EOC histotypes were classified using diagnostic codes provided by UKBB, with “serous”, “undifferentiated”, or “other” classified as HGSOC, a methodology similar to that used in OCAC^22^. Association analyses between genotypes and HGSOC risk were assessed by logistic regression (Fig. 3). We adjusted for the top four PCs and age at recruitment.

### Statistical analyses of BRCA1 and BRCA2 pathogenic variant carriers

Analyses of CIMBA data were limited to carriers of European ancestry, determined by genetic data and multidimensional scaling^22,103^. We used 33,661 common uncorrelated variants (the same set used to calculate PCs) to calculate kinship coefficients between all CIMBA participants and 267 HapMap samples (CHD, JPT, YRI and CEU ancestries). These kinship coefficients were converted to distances and then underwent multidimensional scaling. Using the top two PCs, the proportion of European ancestry for each participant was calculated. Women with >27% non-European ancestry were excluded, ensuring that women with Ashkenazi Jewish ancestry were retained for statistical analyses. Association analyses were performed separately by genotyping array (iCOGS or OncoArray), and separately for *BRCA1* and *BRCA2* PV carriers (Fig. 3). iCOGS and OncoArray associations were combined by fixed-effects inverse-variance weighted meta-analysis to estimate *BRCA1* and *BRCA2* PV carrier specific associations. The association analysis was carried out within a survival analysis framework, by modeling the retrospective likelihood of observing the genotypes conditional on the disease phenotypes to adjust for the non-random ascertainment with respect to disease phenotypes^113,114^. The censoring process followed carriers from birth until the first occurrence of: EOC (including fallopian tube and peritoneal cancers) diagnosis, risk-reducing salpingo-oophorectomy, or study entry. Breast cancer diagnoses were not considered to be a censoring event and EOC was the endpoint of interest. Associations were then assessed using the score test statistic based on the retrospective likelihood^113,114^ assuming *BRCA1* and *BRCA2* PV carrier specific and age-cohort specific EOC incidences^88^. Analyses were stratified by country and Ashkenazi Jewish ancestry, and to account for relatedness between individuals we calculated kinship adjusted variances^115^. Only variants that were available through OncoArray genotyping and imputation were considered, as the majority of samples were available from this genotyping platform (Table 1). As HGSOC is the predominant histotype for both *BRCA1* and *BRCA2* PV carriers, the associations were combined by fixed-effects and inverse variance weighted meta-analysis using the METAL software^116^.

### Meta-analyses

We pooled the combined OCAC and UKBB summary association data (per-allele odds ratios, ORs) with the combined *BRCA1* and *BRCA2* PV carrier summary association data (per-allele hazard ratios, HRs) by fixed-effects inverse-variance weighted meta-analysis using METAL^116^ to give per-allele relative risks (RRs, a combination of population-based ORs and *BRCA1*/*2* carrier HRs) (Fig. 3).

### Eliminating likely statistical artifacts

The associations of all variants with genome-wide significant associations and falling outside known regions were re-evaluated to eliminate likely spurious associations potentially due to unstable effect estimates from strata with small numbers. For this purpose, the associations were re-analyzed, pooling individual level data from OCAC and UKBB, incorporating 29 PCs derived across all genotyping projects (PCs set to 0 for women not in specific studies/projects). The UKBB data were also adjusted for age (set to 0 for the OCAC samples). Any variant with imputation accuracy *r*^2^ < 0.30 within a panel were considered missing for that particular panel. The associations for *BRCA1*/*2* PV carriers were reassessed assuming all study participants came from a single stratum.

We also re-evaluated potentially novel associations with variants in regions proximal to known regions by performing approximate conditional analyses^117^. This approach utilized summary statistics from the combined OCAC, UKBB and *BRCA1/2* PV carrier meta-analysis and the linkage disequilibrium (LD) structure from 111,304 women genotyped on the OncoArray from OCAC and CIMBA.

For variants passing these checks, we calculated Bayesian false-discovery probabilities (BFDPs)^45^. BFDPs are approximate Bayes factors, statistical measures of the strength of evidence in favor of a given statistical model over another. They are akin to false-positive reporting probabilities, aiming to control the number of false positive associations whilst not dismissing too many associations that may merit scientific interest, by including Bayesian priors on an effect or association. BFDPs are especially useful in contexts where many statistical tests are performed, such as GWAS. We assumed prior probabilities of 1:1000 and 1:10,000 variants being truly associated. BFDPs were calculated using the BFDP function from the R gap package^118^.

### Genomic database searches

Variants found to be associated with HGSOC at the genome-wide statistical significance level that also passed the additional checks detailed above, and the nearest genes to these variants, were looked up in genomic resource databases. We accessed dbSNP^74^, Phenoscanner (version 2)^119–121^, PheWeb (version 1.3.15)^122^, GTEx^64^ and eQTLGen^123,124^ (accessed on 15^th^ May 2023).

### Defining credible causal variants

The lead variant (variant with the smallest *P*-value) at each novel region may not be causal. Therefore, we identified lists of credible causal variants (CCVs) that are likely to contain the genetic variant responsible for altering HGSOC risk defined as the set of variants within ±500 kb of the lead variant whose *P*-value was within two orders of magnitude of the lead variant’s *P*-value^125^.

### Development of polygenic risk models

**Fig. 4:**
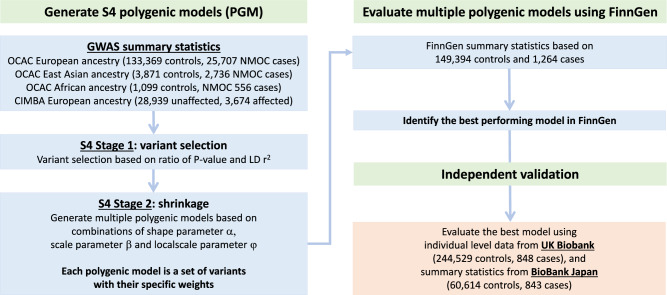
Schema describing the development of polygenic models, determining the optimal model, and validating the resultant polygenic score in European ancestry women from UKBB and East Asian ancestry women from BBJ. S4 select and shrink using summary statistics, PGM polygenic model, NMOC non-mucinous ovarian cancer, LD linkage disequilibrium.

Previous analyses of PGMs specific to HGS, LGS, and mucinous histotypes showed that all histotypes, except mucinous, were associated with either the HGSOC PGS or LGSOC PGS^28^. Genetic correlations between mucinous ovarian cancer and other ovarian cancer histotypes have been estimated to be 0.24, 0.21, 0.23 and −0.21 with high-grade serous ovarian cancer, low-grade serous ovarian cancer, endometrioid ovarian cancer and clear cell, respectively^28^. Whereas the genetic correlations are stronger for pairs of the other non-mucinous histotypes^28^. Thus, the non-mucinous histotypes were combined here^126^. The PGMs were developed on summary statistics using associations obtained from a meta-analysis of the OCAC and CIMBA data (Fig. 4). To maximize sample size and genetic diversity, the OCAC summary statistics came from a meta-analysis of European (133,369 controls, 25,707 cases), East Asian (3871 controls, 2736 cases) and African (1099 controls, 556 cases) ancestry women, considering the associations with non-mucinous OC. Hence, these OCAC summary statistics differed from those obtained from the analyses described in ‘Statistical analyses of OCAC and UKBB data’, as a result of analyzing additional cases from non-mucinous histotypes (and further controls from studies previously excluded because they had no HGSOC cases), and additional women of East Asian and African ancestry. The CIMBA summary statistics were identical to those generated in the analyses described in ‘Statistical analyses of *BRCA1* and *BRCA2* pathogenic variant carriers’.

We developed 1102 PGMs (sets of variants and their weights) for non-mucinous OC using the Select and Shrink with Summary Statistics (S4) method^43,126^. We used two *P*-value to LD *r*^2^ ratios to select variants: (i) P:*r*^2^ < 0.02 that resulted in ~64k variants (562 PGMs tested) and (ii) P:*r*^2^ < 0.15 resulting in ~394k variants (540 models tested). The LD structure was weighted to reflect the average effects from each ancestry based on a subset of OCAC OncoArray data. Each model had different combinations of model hyperparameters (shrinkage parameters controlling small and large variant effect sizes, and an overall shrinkage parameter) that were trained using summary statistics based on 150,658 women (149,394 controls, 1264 EOC cases) from FinnGen (data freeze 8)^38,39^. EOC histotype data was not available for FinnGen, hence we used the associations with overall EOC for PGM training.

Polygenic scores (PGS) are PGMs applied to observed or imputed genotypes. We used the resultant PGM to calculate PGS on individual-level data in the UKBB^36,37^ to test its association with HGSOC and calculated its discriminatory ability for HGSOC by estimating the area under the ROC (AUROC) curve. AUROCs were calculated using the R pROC package auc function^127^.

We assessed the performance of the PGS for women of East Asian ancestry using BBJ data. As individual-level data were unavailable, we used association summary statistics from BBJ^101^ and assessed the PGS performance using a previously described method for evaluating PGS on summary statistic data^126^. The reference panel used consisted of individuals of East Asian ancestry from the 1000 Genomes Project^29^.

We also created candidate PGMs consisting of genotyped variants that could be more easily applied in clinical settings by selecting variants known to reliably genotype from the OncoArray^103^, many of which had been chosen for their relevance to ovarian cancer. We selected a subset of genotyped variants, based on “relative importance”, where the importance of each variant is approximately proportional to p*(1-*p*)*β^2^, where β is the log-RR and *p* is the minor allele frequency for that variant. We ranked each variant based on relative importance and selected the top *N* desired variants.

### Absolute risks of EOC by PGS percentile

We calculated predicted lifetime risks (to age 80 years) of developing EOC for women in the general population (unselected for PV status), *BRCA1* and *BRCA2* PV carriers at the 1^st^, 5^th^, 20^th^, 50^th^ (median), 80^th^, 95^th^ and 99^th^ percentiles of the various PGS distributions, following previously published methodology^128^. To ensure consistency with known EOC risks for the general population, *BRCA1* and *BRCA2* PV carriers, average age-specific EOC incidence rates were constrained over PGS percentiles to agree with external EOC incidence rates for the general population^129^ and *BRCA1*/*2* carriers^130^.

We examined the number of *BRCA2* PV carriers genotyped on the OncoArray that would transition between risk groups (low (<10%) or high (≥10%) lifetime risk) of developing EOC, based on their observed PGS percentile. These risk reclassification analyses were limited to *BRCA2* carriers as their lifetime risks transition over the 10% lifetime risk threshold, whereas a *BRCA1* carrier is already at substantially increased lifetime risk.

### Ethics statement

All study participants provided written informed consent and participated in research studies at the host institute under ethically approved protocols. This study was conducted in accordance with the Declaration of Helsinki.

All study participants provided written informed consent and participated in research or clinical studies at the host institute under ethically approved protocols. The studies and their approving institutes are: Australian site of the Breast Cancer Family Registry (BCFR-AU) - The University of Melbourne Health Sciences Human Ethics Sub-Committee; Northern California site of the Breast Cancer Family Registry (BCFR-NC) - Northern California Cancer Center Institutional Review Board; New York site of the Breast Cancer Family Registry (BCFR-NY) - Columbia University Medical Center Institutional Review Board; Ontario site of the Breast Cancer Family Registry (BCFR-ON) - Mount Sinai Hospital Research Ethics Board; Philadelphia site of the Breast Cancer Family Registry (BCFR-PA) - Institutional Review Board Fox Chase Cancer Center; Utah site of the Breast Cancer Family Registry (BCFR-UT) - Institutional Review Board University of Utah; Baltic Familial Breast and Ovarian Cancer Consortium (BFBOCC) - Centrālā medicīnas ētikas Komiteja; Lietuvos Bioetikos Komitetas; BRCA-gene mutations and breast cancer in South African women (BMBSA) - University of Pretoria and Pretoria Academic Hospitals Ethics Committee; Beckman Research Institute of the City of Hope (BRICOH) - City of Hope Medical Center Institutional Review Board; Copenhagen Breast Cancer Study (CBCS) - De Videnskabsetiske Komiteer I Region Hovedsladen; Spanish National Cancer Centre (CNIO) - Instituto de Salud Carlos III Comité de Bioética y Bienestar Animal; City of Hope Cancer Center (COH) - City of Hope Institutional Review Board; CONsorzio Studi ITaliani sui Tumori Ereditari Alla Mammella (CONSIT TEAM) - Comitato Etico Indipendente della Fondazione IRCCS “Istituto Nazionale dei Tumori”; National Centre for Scientific Research Demokritos (DEMOKRITOS) - Bioethics committee of NCSR “Demokritos”, 240/EHΔ/11.3; National Centre for Scientific Research Demokritos (DEMOKRITOS) - Papageorgiou Hospital Ethics Committee; Dana Farber Cancer Institute (DFCI) - Dana Farber Cancer Institute Institutional Review Board; Deutsches Krebsforschungszentrum (DKFZ) - Ethik-Kommission des Klinikums der Universität; Deutsches Krebsforschungszentrum (DKFZ) - Hospital Universitario de San Ignacio Comité de Investigaciones y Etica; Deutsches Krebsforschungszentrum (DKFZ) - Shaukat Khanum Memorial Cancer Hospital and Research Centre Institutional Review Board; Epidemiological study of BRCA1 and BRCA2 mutation carriers (EMBRACE) - Anglia & Oxford MREC; Fox Chase Cancer Center (FCCC) - Institutional Review Board Fox Chase Cancer Center; Fundación Pública Galega de Medicina Xenómica - Comite Autonomico de Etica da Investigacion de Galicia; German Consortium of Hereditary Breast and Ovarian Cancer (GC-HBOC) - Ethik-Kommission der Medizinischen Fakultät der Universät zu Köln; Genetic Modifiers of cancer risk in BRCA1/2 mutation carriers (GEMO) - Comité consultatif sur le traitement de I’information en matière de recherche dans le domaine de la santé; Georgetown University (GEORGETOWN) - MedStar Research Institute - Georgetown University Oncology Institutional Review Board; Ghent University Hospital (G-FAST) - Universitair Ziekenhuis Gent - Ethics Committee; Hospital Clinico San Carlos (HCSC) - Comité Ético de Investigación Clínia Hospital Clínico San Carlos; Helsinki Breast Cancer Study (HEBCS) - Helsingin ja uudenmaan sairaanhoitopiiri (Helsinki University Central Hospital ethics committee); HEreditary Breast and Ovarian study Netherlands (HEBON) - Protocol Toetsingscommissie van het Nederlands Kanker Instituut/Antoni van Leeuwenhoek Ziekenhuis; Molecular Genetic Studies of Breast- and Ovarian Cancer in Hungary (HUNBOCS) - Institutional Review Board of the Hungarian National Institute of Oncology; University Hospital Vall d’Hebron (HVH) - The Hospital Universitario Vall d’Hebron Clinical Research Ethics Committee; Institut Català d’Oncologia (ICO) - Catalan Institute of Oncology Institutional Review Board; International Hereditary Cancer Centre (IHCC) - Komisji Bioetycznej Pomorskiej Akademii Medycznej (Pomeranian Medical University Bioethics Committee); Iceland Landspitali - University Hospital (ILUH) - Vísindasiđanefnd National Boethics Committee; Interdisciplinary Health Research International Team Breast Cancer Susceptibility (INHERIT) - Comité d'éthique de la recherche du Centre Hospitalier Universitaire de Québec; Istituto Oncologico Veneto Hereditary Breast and Ovarian Cancer Study (IOVHBOCS) - Centro Oncologico Regionale Azienda Ospedale Di Padova Comitato Etico; Portuguese Oncology Institute-Porto Breast Cancer Study - COMISSÃO DE ÉTICA PARA A SAÚDE (CES); Kathleen Cuningham Foundation Consortium for Research into Familial Breast Cancer (KCONFAB) - Queensland Institute of Medical Research - Human Research Ethics Committee; Kathleen Cuningham Foundation Consortium for Research into Familial Breast Cancer (KCONFAB) - Peter MacCallum Cancer Centre Ethics Committee; University of Kansas Medical Center(KUMC) - The University of Kansas Medical Center Human Research Protection Program; Mayo Clinic (MAYO) - Mayo Clinic Institutional Review Boards; McGill University (MCGILL) - McGill Faculty of Medicine Institutional Review Board; Modifier Study of Quantitative Effects on Disease (MOD-SQUAD) - Mayo Clinic Institutional Review Boards; Memorial Sloane Kettering Cancer Center (MSKCC) - Human Biospecimen Utilization Committee; Memorial Sloane Kettering Cancer Center (MSKCC) - Memorial Sloan-Kettering Cancer Center IRB; General Hospital Vienna (MUV) - Ethikkommission der Medizinischen Universität Wien; Women’s College Research Institute Hereditary Breast and Ovarian Cancer Study - University of Toronto Health Sciences Review Ethics Board; National Cancer Institute (NCI) - NIH Ethics Office; National Israeli Cancer Control Center (NICCC) - Carmel Medical Center Institutional Review Board (Helsinki Committee); N.N. Petrov Institute of Oncology (NNPIO) - N.N. Petrov Institional Ethical Committee; NorthShore University HealthSystem (NORTHSHORE) - Institutional Review Board of NorthShore University HealthSystem; NRG Oncology (NRG_ONCOLOGY) - Cancer Prevention and Control Protocol Review Committee; Ontario Cancer Genetics Network (OCGN) - University Health Network Research Ethics Board; The Ohio State University Comprehensive Cancer Center (MACBRCA) - The Ohio State University Cancer Institutional Review Board; Odense University Hospital (OUH) - Den Videnskabsetiske Komité for Region Syddanmark; Pisa Breast Cancer Study (PBCS) - Azienda Ospedaliera Pisana Comitato Etico per lo studio del farmaco sull’uomo; Sheba Medical Centre - Chaim Sheba Medical Center IRB; Swedish Breast Cancer Study (SWE-BRCA) - Regionala Etikprövningsnämnden Stockholm; University of Chicago (UCHICAGO) - The University of Chicago Biological Sciences Division. Institutional Review Board (BSD IRB); University of California Los Angeles (UCLA) - UCLA Institutional Review Board (UCLA IRB); University of California San Francisco (UCSF) - Human Research Protection Program Institutional Review Board (IRB); UK and Gilda Radner Familial Ovarian Cancer Registries (UKGRFOCR) - Roswell Park Cancer Institute IRB; UK and Gilda Radner Familial Ovarian Cancer Registries (UKGRFOCR) - Cambridge Local Research Ethics Committee; University of Pennsylvania (UPENN) - University of Pennsylvania Institutional Review Board; Cancer Family Registry University of Pittsburg (UPITT) - University of Pittsburgh Institutional Review Board; University of Texas MD Anderson Cancer Center (UTMDACC) - University of Texas MD Anderson Cancer Center Office of Protocol Research Institutional Review Board; Victorian Familial Cancer Trials Group (VFCTG) - Peter MacCallum Cancer Centre Ethics Committee; Women’s Cancer Program at Cedars-Sinai Medical Center (WCP) - (Cedars-Sinai Medical Center) CSMC Institutional Review Board. GynBiobank (WMH) – Western Sydney Local Health District Human Research Ethics Committee.

## Supplementary information

Supplementary Information

Supplementary Data

## Data Availability

GWAS summary statistics from OCAC and CIMBA are publicly available from the GWAS Catalog under study accession identifiers GCST90455658 (OCAC), GCST90455659 (CIMBA BRCA1 PV carriers), GCST90455660 (CIMBA BRCA2 PV carriers). The summary statistics from the meta-analysis of OCAC, UKBB and CIMBA associations are also available under study accession identifier GCST90455661. Variants included in the polygenic scores and their corresponding weights are publicly available from the PGS Catalog under identifiers PGS005086 (PGS_64518_), PGS005087 (PGS_5957_), PGS005088 (PGS_3448_) and PGS005089 (PGS_400_). The FinnGen summary statistics used were from data freeze 8. The summary statistics across all data freezes are available at https://www.finngen.fi/en/access_results and the data freeze 8 summary statistics are available at https://r8.finngen.fi/. The Biobank Japan summary statistics used were from Sakaue et al.^101^. The summary statistic data for all traits are available at https://pheweb.jp/ and the ovarian cancer summary statistic data can be accessed here https://pheweb.jp/pheno/OvC.

## Code availability

Code and software used to implement analyses is publicly available at https://github.com/CCGE-Cambridge/OCAC_CIMBA_HGSOC.
