# Supplementary material for: Genome-wide association study of 398,238 women unveils seven loci associated with high-grade serous ovarian cancer
Source: NPJ Genom Med. 2025 Nov 20;10:73. doi: 10.1038/s41525-025-00529-w (PMC12635163; doi:10.1038/s41525-025-00529-w)

SUPPLEMENTARY RESULTS

S4 PGM hyperparameters

The best performing PGS contained 64,518 variants, that had S4 hyperparameters: $\alpha = 0.1$, $\beta = 0.8$ and $\varphi = 8 \times 10^{-8}$. α controls the degree of shrinkage towards zero for the model coefficients. A smaller α means more shrinkage, which implies more sparsity and less variance. β controls the amount of shrinkage for extreme values of the model coefficients. A larger β means more shrinkage for extreme values. φ controls the overall scale of the shrinkage in the model. A larger φ means a larger scale, which implies more variability and less shrinkage for all coefficients.

Supplementary Table 1: Numbers of controls and EOC cases by genotyping array and country from population-based studies.

Panel	Stratum	Study	Controls	Cases (invasive, serous, mucinous LMP)	HGSOC	ENOC	CCOC	MOC	LGSOC	Other
iCOGS	Australia	AUS	76	37	30	3	1	2	1	0
	Australia	BCA	960	0	0	0	0	0	0	0
	Australia	MCC	32	8	4	1	1	0	0	2
	Belarus	HMO	99	79	66	7	0	4	0	2
	Belgium	BEL	521	13	8	0	3	2	0	0
	Canada	BCA	648	0	0	0	0	0	0	0
	Canada	OVA	549	496	278	52	29	66	52	19
	Canada	TOR	32	19	11	6	2	0	0	0
	Denmark	BCA	4519	0	0	0	0	0	0	0
	Denmark	PVD	0	4	3	1	0	0	0	0
	Finland	BCA	1301	0	0	0	0	0	0	0
	Finland	HOC	263	39	25	3	3	8	0	0
	Germany	BAV	132	27	17	3	0	3	3	1
	Germany	BCA	4034	0	0	0	0	0	0	0
	Germany	GER	42	12	5	2	0	2	2	1
	Germany	HJO	12	143	103	13	1	7	5	14
	Germany	HSK	0	38	25	6	0	0	6	1
	Norway	BCA	217	0	0	0	0	0	0	0
	Norway	NOR	31	58	40	7	3	4	2	2
	Poland	BCA	324	0	0	0	0	0	0	0
	Poland	POC	413	319	256	30	6	27	0	0
	Poland	POL	43	5	3	1	0	1	0	0
	Poland	WOC	0	4	4	0	0	0	0	0
	UK	BCA	4514	0	0	0	0	0	0	0
	UK	RMH	0	4	1	1	1	0	0	1
	UK	SEA	4425	36	12	7	0	11	4	2
	UK	SOC	0	20	7	2	1	6	1	3
	UK	SRO	0	156	112	17	8	3	3	13
	UK	UKO	149	5	3	0	0	2	0	0
	UK	UKR	0	1	1	0	0	0	0	0
	USA	BCA	2589	0	0	0	0	0	0	0
	USA	DOV	52	38	22	2	1	6	5	2
	USA	GRR	0	111	79	18	9	4	0	1
	USA	HAW	1	0	0	0	0	0	0	0
	USA	HOP	278	181	124	13	6	12	16	10
	USA	LAX	0	3	3	0	0	0	0	0
	USA	MAY	2	3	1	0	0	0	1	1
	USA	MDA	87	319	262	18	4	24	0	11
	USA	MSK	399	274	237	13	12	0	11	1
	USA	NCO	0	12	2	1	1	3	5	0
	USA	NEC	1004	891	413	140	33	115	175	15
	USA	NHS	367	67	50	6	1	5	4	1
	USA	NJO	183	171	104	28	20	7	8	4
	USA	ORE	0	19	10	0	1	4	4	0
	USA	STA	17	77	54	8	7	3	4	1
	USA	UCI	36	180	96	17	14	26	25	2
	USA	USC	355	324	190	39	13	39	38	5
GWAS	USA	BWH	0	22	22	0	0	0	0	0
	USA	NEC	114	70	70	0	0	0	0	0
	Canada	TOR	513	479	296	106	34	40	0	3
	Poland	POL	555	234	134	28	9	16	18	29
	USA	MAY	438	508	371	31	20	37	47	2
	USA	PLC	1	0	0	0	0	0	0	0
	USA	POC	0	1	1	0	0	0	0	0
	UK	RMH	0	145	75	26	17	18	0	9
	UK	SEA	0	1139	365	200	127	127	255	65
	UK	UKO	0	484	284	75	42	52	12	19
	UK	UKR	0	37	20	5	1	3	1	7
	UK	WTC	6116	0	0	0	0	0	0	0
	USA	MAY	515	26	18	3	1	2	2	0
	USA	NCO	650	102	63	18	9	5	6	1
	USA	TBO	169	38	26	1	5	5	1	0
OncoArray	Australia	AUS	1139	1501	897	127	70	194	177	36
	Australia	BCA	2312	0	0	0	0	0	0	0
	Australia	MCC	142	131	67	11	7	22	20	4
	Australia	OPL	0	482	371	29	29	24	19	10
	Australia	RBH	0	139	92	18	11	10	2	6
	Australia	WMH	0	145	109	13	5	0	15	3
	Belarus	HMO	247	65	54	5	1	3	0	2
	Belgium	BEL	1264	725	470	45	25	88	94	3
	Canada	BCA	1360	0	0	0	0	0	0	0
	Canada	OVA	724	636	333	81	45	88	73	16
	Canada	TOR	164	442	263	60	16	69	29	5
	Canada	UHN	0	177	130	22	12	4	8	1
	Canada	VAN	0	172	139	3	6	16	8	0
	Denmark	BCA	716	0	0	0	0	0	0	0
	Denmark	PVD	0	195	151	15	9	11	9	0
	Europe	EPC	870	418	335	36	14	31	0	2
	Finland	BCA	245	0	0	0	0	0	0	0
	Finland	HOC	278	263	154	35	16	50	7	1
	Germany	BAV	286	289	193	27	13	24	29	3
	Germany	BCA	4460	0	0	0	0	0	0	0
	Germany	GER	376	199	110	18	6	27	25	13
	Germany	HJO	0	212	134	26	5	10	13	24
	Germany	HSK	0	122	98	12	0	1	7	4
	Greece	BCA	512	0	0	0	0	0	0	0
	Greece	GRC	0	329	238	38	18	15	13	7
	Netherlands	BCA	1763	0	0	0	0	0	0	0

	Netherlands	NTH	584	254	108	63	20	33	23	7
	Norway	NOR	344	183	123	20	8	15	12	5
	Poland	BCA	1553	0	0	0	0	0	0	0
	Poland	POC	173	165	124	18	7	10	0	6
	Poland	POL	0	46	28	9	2	3	3	1
	Poland	WOC	205	200	150	20	17	10	1	2
	Russia	HUO	113	47	18	0	1	2	2	24
	Spain	BCA	1036	0	0	0	0	0	0	0
	Spain	CNI	178	80	48	11	7	5	6	3
	Sweden	BCA	6706	0	0	0	0	0	0	0
	Sweden	LUN	1526	35	15	3	0	9	7	1
	Sweden	SMC	93	83	67	10	2	3	0	1
	UK	BCA	4643	0	0	0	0	0	0	0
	UK	BGS	0	214	127	21	7	35	17	7
	UK	CAM	0	224	187	9	16	0	0	12
	UK	ICN	0	390	297	29	39	3	13	9
	UK	RMH	0	37	24	4	1	6	0	2
	UK	SEA	1782	1045	365	65	53	224	307	31
	UK	SOC	0	291	145	63	10	45	16	12
	UK	SRO	0	3	1	0	0	0	2	0
	UK	UKO	971	307	177	47	34	29	12	8
	UK	UKR	0	12	7	1	1	0	1	2
	USA	BCA	21174	0	0	0	0	0	0	0
	USA	BVU	391	135	100	15	11	3	4	2
	USA	DKE	0	80	58	7	6	3	3	3
	USA	DOV	1459	1226	637	147	67	149	207	19
	USA	GRR	0	22	18	1	2	1	0	0
	USA	HAW	171	104	57	14	5	15	11	2
	USA	HOP	1189	507	288	71	36	38	38	36
	USA	LAX	0	376	257	19	10	12	77	1
	USA	MAC	0	207	168	19	12	3	3	2
	USA	MAY	261	635	434	83	42	34	36	6
	USA	MDA	297	305	225	15	14	8	31	12
	USA	MEC	6	5	4	1	0	0	0	0
	USA	MOF	401	365	269	28	15	24	18	11
	USA	MSK	205	202	175	9	6	0	3	9
	USA	NCO	179	833	430	96	70	80	150	7
	USA	NEC	566	494	248	97	31	60	50	8
	USA	NHS	314	320	173	49	16	37	38	7
	USA	ORE	0	81	61	10	1	1	7	1
	USA	PLC	1098	255	189	19	8	10	17	12
	USA	RPC	0	96	71	7	3	6	1	8
	USA	SIS	1242	117	55	6	3	3	2	48
	USA	STA	311	276	129	27	14	37	63	6
	USA	TBO	1	176	131	25	7	8	2	3
	USA	UCI	292	257	87	35	10	49	75	1
	USA	USC	788	605	342	51	26	77	92	17
**Total**			**104887**	**25415**	**15361**	**2823**	**1394**	**2478**	**2615**	**744**

Cases is the sum of the specific histotypes in columns (HGSOC to Other).

LMP = low malignant potential, HGSOC = high-grade serous ovarian cancer, ENOC = endometrioid ovarian cancer, CCOC = clear cell ovarian cancer, MOC = mucinous ovarian cancer.

Supplementary Table 2: Numbers of unaffected *BRCA1*/*2* carriers and carriers diagnosed with EOC, by CIMBA study.

Study	Country	*BRCA1* carriers iCOGS		*BRCA1* carriers OncoArray		*BRCA2* carriers iCOGS		*BRCA2* carriers OncoArray		*BRCA1*+BRCA2 carriers		Total
		Unaffected	Affected	Unaffected	Affected	Unaffected	Affected	Unaffected	Affected	Unaffected[a]	Affected[b]	
BCFR-AU	Australia	2	0	35	2	0	0	38	1	75	3	**78**
BCFR-NC	USA	2	0	35	1	0	0	27	0	64	1	**65**
BCFR-NY	USA	6	2	54	8	3	0	43	9	106	19	**125**
BCFR-ON	Canada	9	0	115	6	5	0	83	1	212	7	**219**
BCFR-PA	USA	24	6	39	5	4	1	6	0	73	12	**85**
BCFR-UT	USA	1	0	183	18	1	0	143	8	328	26	**354**
BFBOCC	Latvia, Lithuania	17	7	151	93	0	0	15	3	183	103	**286**
BIDMC	USA	2	0	70	14	1	0	51	0	124	14	**138**
BMBSA	South Africa	2	1	47	11	6	0	126	14	181	26	**207**
BRICOH	USA	17	3	133	16	9	1	115	8	274	28	**302**
CBCS	Denmark	106	26	152	29	21	2	118	9	397	66	**463**
CCGCRN	USA	13	1	197	23	11	0	133	8	354	32	**386**
CNIO	Spain	77	15	60	3	202	15	55	4	394	37	**431**
CONSIT TEAM	Italy	324	122	408	127	171	17	277	36	1180	302	**1482**
DEMOKRITOS	Greece	22	10	161	52	0	0	31	1	214	63	**277**
DFCI	USA	6	0	131	14	2	0	123	3	262	17	**279**
DKFZ	Germany, Pakistan, Colombia	2	0	54	1	1	0	21	3	78	4	**82**
EMBRACE	UK, Ireland	24	2	1725	182	26	2	1754	111	3529	297	**3826**
FCCC	USA	28	11	65	10	8	1	34	8	135	30	**165**
FPGMX	Spain	0	0	85	23	0	0	69	6	154	29	**183**
G-FAST	Belgium	121	11	163	26	0	0	157	4	441	41	**482**
GC-HBOC	Germany	145	20	1688	261	74	6	1044	76	2951	363	**3314**
GEMO	France	183	41	1209	263	230	20	835	72	2457	396	**2853**
GENEPSO	France	0	0	168	6	0	0	91	0	259	6	**265**
GEORGETOWN	USA	3	0	11	0	0	0	0	0	14	0	**14**
GOG	USA, Australia	11	0	319	0	13	0	291	0	634	0	**634**
HCSC	Spain	8	3	122	19	6	0	141	12	277	34	**311**
HEBCS	Finland	7	1	98	19	3	0	119	10	227	30	**257**
HEBON	Netherlands	287	49	1185	74	112	12	833	35	2417	170	**2587**
HUNBOCS	Hungary	0	0	242	38	0	0	104	9	346	47	**393**
HVH	Spain	1	0	98	20	0	0	121	7	220	27	**247**
ICO	Spain	4	2	226	53	2	0	314	34	546	89	**635**
IHCC	Poland	462	36	190	8	0	0	0	0	652	44	**696**
ILUH	Iceland	0	0	0	0	5	0	133	7	138	7	**145**
INHERIT	Canada	7	1	77	12	1	0	76	3	161	16	**177**
IOVHBOCS	Italy	5	4	132	72	3	0	141	25	281	101	**382**
IPOBCS	Portugal	1	2	99	16	0	0	157	4	257	22	**279**
KCONFAB	Australia	45	3	655	57	29	1	548	14	1277	75	**1352**
KUMC	USA	0	0	24	3	0	0	12	0	36	3	**39**
MAYO	USA	18	4	219	23	12	0	115	10	364	37	**401**
MCGILL	Canada	0	0	50	4	0	0	33	1	83	5	**88**
MODSQUAD	Czechia	132	41	0	0	0	0	0	0	132	41	**173**
MSKCC	USA	88	3	320	55	17	1	313	42	738	101	**839**
MUV	Austria	21	1	477	74	9	0	253	14	760	89	**849**
NAROD	Canada	124	21	0	0	0	0	0	0	124	21	**145**
NCI	USA	6	1	140	10	0	0	77	6	223	17	**240**
NICCC	Israel	0	0	0	0	162	7	0	0	162	7	**169**
NNPIO	Russia	4	1	48	18	0	0	2	0	54	19	**73**
NORTHSHORE	USA	0	0	68	12	1	1	47	8	116	21	**137**
OCGN	Canada	7	2	164	40	6	0	149	21	326	63	**389**
OSU CCG	USA	17	1	73	11	7	2	86	12	183	26	**209**
OUH	Denmark	17	3	465	83	7	1	383	42	872	129	**1001**
PBCS	Italy	6	5	66	22	0	0	7	0	79	27	**106**
SMC	Israel	82	16	141	24	22	2	72	8	317	50	**367**
SWE-BRCA	Sweden	67	23	327	98	6	1	53	11	453	133	**586**
UCHICAGO	USA	7	0	86	8	3	1	54	3	150	12	**162**
UCLA	USA	0	0	0	0	39	2	0	0	39	2	**41**
UCSF	USA	23	8	70	19	2	1	55	8	150	36	**186**
UKGRFOCR	UK, USA	2	3	23	27	0	0	5	10	30	40	**70**
UPENN	USA	21	11	391	64	9	0	319	26	740	101	**841**
UPITT	USA	0	0	133	19	0	0	93	6	226	25	**251**
UTMDACC	USA	62	10	37	5	0	0	56	12	155	27	**182**
VFCTG	Australia	1	2	172	31	0	0	186	11	359	44	**403**
WCP	USA	14	0	129	10	3	0	50	4	196	14	**210**
**Total**		**2693**	**535**	**14205**	**2242**	**1254**	**97**	**10787**	**800**	**28939**	**3674**	**32613**

[a] Sum of all unaffected *BRCA1* (iCOGS and OncoArray) and *BRCA2* (iCOGS and OncoArray) carriers.

[b] Sum of all affected *BRCA1* (iCOGS and OncoArray) and *BRCA2* (iCOGS and OncoArray) carriers.

Supplementary Table 3: Previously identified genomic regions associated with EOC.

							OCAC associations[a]						Meta-analysis: OCAC+UKBB+carrier associations[b]					Meta-analysis: OCAC+UKBB associations[b]					*BRCA1* carrier associations[b]						*BRCA2* carrier associations[b]					
**Publication**	**Cytoband**	**Region**	**SNP**	**Position[c]**	**Nearest gene**	**Alleles[d]**	Histotype	EAF	OR	95% CI		P	EAF	RR	95% CI		P	EAF	OR	95% CI		P	EAF	HR	95% CI		P	P_{het}[e]	EAF	HR	95% CI		P	P_{het}[e]
Kuchenbaecker 2015[1]*	1p36.12	chr1:21641722-22641722	rs3820282	22141722	*WNT4*	C/T	NMOC	0.156	1.09	1.06	1.11	4.87E-10	0.153	1.08	1.05	1.11	1.21E-06	0.156	1.07	1.04	1.11	6.27E-05	0.138	1.13	1.04	1.23	4.32E-03	2.4E-01	0.138	1.07	0.92	1.23	3.79E-01	9.6E-01
Kuchenbaecker 2015[1]*	1p34.3	chr1:37116521-38116450	rs12039431	37616450	*RSPO1*	G/A	HGSOC	0.260	1.11	1.08	1.14	5.06E-17	0.261	1.11	1.08	1.14	5.06E-17	0.259	1.11	1.08	1.14	5.15E-14	0.268	1.09	1.03	1.17	6.63E-03	7.0E-01	0.271	1.16	1.04	1.30	7.55E-03	4.3E-01
Phelan 2017[2]*	2q13	chr2:110525257-111658369		111025257	*ACOXL*	G/A	HGSOC	0.163	1.09	1.05	1.12	3.71E-08	0.162	1.09	1.05	1.12	3.71E-08	0.164	1.09	1.05	1.12	2.88E-07	0.155	1.09	1.01	1.18	3.35E-02	9.6E-01	0.154	1.03	0.90	1.18	7.06E-01	4.1E-01
Kar 2016[3]**	2q13	chr2:110525257-111658369	rs17041869 [f]	111138666	*BCL2L11*	A/G	HGSOC	0.130	1.06	1.02	1.09	6.32E-04	0.128	1.06	1.02	1.09	6.32E-04	0.131	1.07	1.03	1.11	3.83E-04	0.114	1.05	0.96	1.15	3.33E-01	6.8E-01	0.119	0.95	0.81	1.10	4.72E-01	1.3E-01
Dareng 2024[4]	2q13	chr2:110525257-111658369	rs1470053	111158369		G/T	LGSOC	0.185	0.86	0.80	0.93	1.25E-04	0.182	1.00	0.97	1.03	9.65E-01	0.182	0.99	0.96	1.02	4.32E-01	0.184	1.07	0.99	1.15	9.14E-02	6.3E-02	0.181	1.03	0.91	1.18	6.10E-01	4.9E-01
Dareng 2024[4]	2q13	chr2:112716387-113716387	rs895412 [f]	113216387	*PAX8*	T/C	HGSOC	0.477	1.05	1.03	1.08	5.29E-06	0.478	1.05	1.03	1.08	5.29E-06	0.477	1.06	1.03	1.08	1.34E-05	0.482	1.06	1.00	1.12	6.36E-02	9.8E-01	0.485	0.99	0.89	1.09	7.83E-01	1.9E-01
Kelemen 2015[5]	2q13	chr2:112721787-113721787		113221787	*PAX8*	G/A	MOC	0.152	1.26	1.17	1.36	2.61E-10	0.150	1.01	0.98	1.04	4.60E-01	0.152	1.01	0.98	1.05	4.51E-01	0.141	1.00	0.92	1.09	9.61E-01	7.4E-01	0.139	1.03	0.89	1.19	7.34E-01	8.8E-01
Dareng 2024[4]	2q14.2	chr2:119888925-120888925	rs72827480	120388925		C/T	MOC	0.600	1.15	1.09	1.22	1.07E-06	0.602	1.02	1.00	1.05	6.15E-02	0.601	1.02	0.99	1.05	1.34E-01	0.609	1.02	0.96	1.08	5.47E-01	9.8E-01	0.604	1.07	0.97	1.19	1.85E-01	3.5E-01
Goode 2010[6]	2q31.1	chr2:175674850-177143894	rs6433571	176174850	*HAGLR*	T/G	NMOC	0.313	1.11	1.09	1.13	3.94E-25	0.318	1.11	1.09	1.14	1.32E-19	0.319	1.12	1.09	1.15	2.32E-18	0.319	1.06	0.99	1.13	7.35E-02	9.3E-02	0.310	1.14	1.02	1.26	2.00E-02	8.2E-01
Kelemen 2015[5]*	2q31.1	chr2:175674850-177143894	rs2594950	176643894	*LINC01117*	C/G	MOC	0.660	1.13	1.06	1.20	6.49E-05	0.660	1.02	1.00	1.05	5.94E-02	0.660	1.03	1.00	1.05	4.91E-02	0.658	1.01	0.95	1.07	8.14E-01	5.9E-01	0.656	1.01	0.90	1.12	9.12E-01	7.2E-01
Phelan 2017[2]	3q23	chr3:138630701-139630701	rs112071820	139130701		T/C	MOC	0.289	1.29	1.21	1.37	6.67E-17	0.289	1.01	0.99	1.04	3.07E-01	0.289	1.01	0.98	1.04	5.12E-01	0.290	1.03	0.97	1.10	3.50E-01	5.4E-01	0.288	1.02	0.91	1.14	7.11E-01	8.4E-01
Pharoah 2013[7]	3q25.31	chr3:156184698-157184698		156684698	*TIPARP*	C/T	HGSOC	0.051	1.56	1.49	1.63	7.02E-83	0.050	1.56	1.49	1.63	7.02E-83	0.051	1.57	1.49	1.64	3.04E-73	0.045	1.43	1.24	1.64	6.00E-07	2.2E-01	0.045	1.77	1.39	2.25	2.72E-06	3.2E-01
Phelan 2017[2]	3q28	chr3:190314093-191314093		190814093		A/G	NMOC	0.686	1.07	1.05	1.10	2.13E-11	0.706	1.07	1.04	1.09	2.34E-07	0.710	1.07	1.04	1.10	2.02E-06	0.693	1.06	1.00	1.13	6.36E-02	8.8E-01	0.694	1.05	0.94	1.17	3.74E-01	7.8E-01
Dareng 2024[4]	4q13.3	chr4:69226524-70226524	rs4149419	69726524	*SULTB1*	T/G	HGSOC	0.642	1.07	1.04	1.09	2.18E-08	0.643	1.07	1.04	1.09	2.18E-08	0.641	1.07	1.05	1.10	3.77E-08	0.653	1.05	0.99	1.12	1.17E-01	5.0E-01	0.651	1.01	0.92	1.12	7.82E-01	2.9E-01
Kuchenbaecker 2015[1]*	4q26	chr4:118546750-119546750		119046750		A/G	NMOC	0.327	1.05	1.03	1.07	2.14E-06	0.330	1.06	1.03	1.08	1.86E-06	0.327	1.06	1.03	1.09	5.41E-06	0.342	1.02	0.96	1.09	4.64E-01	2.8E-01	0.339	1.10	0.99	1.22	6.72E-02	4.9E-01
Couch 2013[8]	4q32.3	chr4:164487569-166700821	rs4691139	164987569		A/G	CCOC	0.465	1.02	0.95	1.10	5.38E-01	0.468	1.03	1.00	1.05	2.44E-02	0.465	1.01	0.99	1.04	3.42E-01	0.480	1.14	1.08	1.21	5.04E-06	1.2E-04	0.489	0.93	0.84	1.02	1.40E-01	9.7E-02
Phelan 2017[2]	4q32.3	chr4:164487569-166700821		166200821		T/C	LGSOC	0.214	1.15	1.07	1.23	1.41E-04	0.199	1.02	0.99	1.04	2.97E-01	0.192	1.02	0.99	1.05	2.62E-01	0.229	1.03	0.96	1.11	4.22E-01	7.6E-01	0.229	0.93	0.82	1.05	2.41E-01	1.6E-01
Bojesen 2013[9]	5p15.33	chr5:779675-1795234	rs10069690	1279675	*TERT*	C/T	HGSOC	0.258	1.11	1.08	1.14	4.19E-17	0.260	1.11	1.08	1.14	4.19E-17	0.257	1.15	1.12	1.18	7.79E-24	0.275	0.92	0.87	0.98	1.44E-02	6.8E-10	0.269	1.08	0.96	1.20	1.86E-01	2.6E-01
Bojesen 2013[9]	5p15.33	chr5:779675-1795234	rs7705526	1285859		C/A	LGSOC	0.328	1.31	1.23	1.39	5.53E-20	0.329	1.08	1.05	1.10	8.22E-11	0.326	1.10	1.07	1.13	1.01E-12	0.341	0.98	0.92	1.04	5.70E-01	9.9E-04	0.342	1.08	0.97	1.19	1.68E-01	7.0E-01
Dareng 2024[4]	5p15.33	chr5:779675-1795234	rs2853677	1287079		A/G	LGSOC	0.429	1.24	1.17	1.31	1.36E-13	0.426	1.05	1.02	1.07	7.14E-05	0.424	1.06	1.03	1.08	1.17E-05	0.432	0.99	0.93	1.05	6.44E-01	3.3E-02	0.440	1.05	0.95	1.16	3.45E-01	8.9E-01
Dareng 2024[4]	5p15.33	chr5:779675-1795234	rs2853669	1295234		G/A	NMOC	0.711	1.03	1.01	1.05	1.02E-02	0.691	1.04	1.02	1.06	1.28E-03	0.689	1.05	1.02	1.08	2.02E-04	0.699	0.99	0.93	1.05	6.91E-01	7.0E-02	0.692	1.01	0.91	1.12	8.86E-01	4.5E-01
Dareng 2024[4]	5q11.2	chr5:54680727-55680728	rs336126	55180728		A/G	NMOC	0.287	1.06	1.04	1.09	8.94E-09	0.256	1.06	1.03	1.08	1.96E-05	0.254	1.07	1.04	1.11	4.56E-07	0.263	0.99	0.92	1.06	7.40E-01	2.4E-02	0.263	0.96	0.86	1.08	5.06E-01	6.5E-02
Phelan 2017[2]	5q12.3	chr5:66329868-67329868	rs555025179	66829868		T/G	EnOC	0.483	0.89	0.84	0.94	9.58E-06	0.486	1.00	0.98	1.03	7.71E-01	0.483	1.00	0.98	1.03	7.72E-01	0.500	0.98	0.93	1.04	5.74E-01	5.3E-01	0.490	1.06	0.96	1.17	2.71E-01	3.2E-01
Kuchenbaecker 2015[1]*	6p22.1	chr6:27968282-28968283		28468283		T/C	NMOC	0.717	1.05	1.03	1.07	2.31E-06	0.719	1.06	1.03	1.08	7.53E-06	0.718	1.06	1.03	1.08	1.07E-04	0.728	1.05	0.99	1.12	1.20E-01	9.4E-01	0.719	1.11	0.99	1.24	6.75E-02	4.0E-01
Phelan 2017[2]	8q21.11	chr8:75732128-76732128		76232128		G/A	LGSOC	0.971	1.54	1.26	1.89	2.10E-05	0.970	1.04	0.97	1.11	2.31E-01	0.969	1.03	0.96	1.11	3.97E-01	0.970	1.04	0.88	1.24	6.58E-01	9.4E-01	0.972	1.20	0.89	1.62	2.22E-01	3.2E-01
Pharoah 2013[7]	8q21.13	chr8:81241409-82241409		81741409		A/G	HGSOC	0.069	1.20	1.15	1.24	5.91E-18	0.070	1.20	1.15	1.24	5.91E-18	0.069	1.22	1.17	1.27	2.97E-18	0.070	1.09	0.97	1.22	1.28E-01	8.0E-02	0.072	1.08	0.89	1.30	4.42E-01	2.2E-01
Dareng 2024[4]	8q24.21	chr8:126575659-129029685	rs6470494	127075659		C/T	MOC	0.280	1.12	1.06	1.19	1.28E-04	0.281	1.00	0.98	1.03	8.20E-01	0.280	1.01	0.98	1.03	5.98E-01	0.280	0.97	0.91	1.03	3.43E-01	2.8E-01	0.286	1.03	0.92	1.15	6.20E-01	7.2E-01
Phelan 2017[2]*	8q24.21	chr8:126575659-129029685	rs9886651	127805637		A/G	NMOC	0.458	1.08	1.06	1.10	4.62E-16	0.460	1.10	1.07	1.12	2.50E-16	0.459	1.09	1.06	1.12	3.49E-12	0.463	1.12	1.05	1.18	2.00E-04	4.7E-01	0.455	1.13	1.02	1.24	1.58E-02	5.1E-01
Dareng 2024[4]	8q24.21	chr8:126575659-129029685	rs7833298	128068411		C/G	HGSOC	0.613	1.07	1.05	1.10	1.17E-09	0.612	1.07	1.05	1.10	1.17E-09	0.614	1.08	1.05	1.11	1.83E-09	0.607	1.03	0.97	1.09	3.64E-01	1.3E-01	0.603	1.08	0.98	1.20	1.24E-01	9.7E-01
Goode 2010[6]	8q24.21	chr8:126575659-129029685	rs10088218	128529685		A/G	HGSOC	0.870	1.24	1.19	1.28	7.73E-34	0.870	1.24	1.19	1.28	7.73E-34	0.871	1.26	1.21	1.31	4.83E-31	0.870	1.16	1.07	1.26	5.73E-04	8.9E-02	0.867	1.17	1.01	1.35	3.55E-02	3.4E-01
Song 2009[10]	9p22.2	chr9:16414718-17414718	rs3814113	16914718	*BNC2*	A/G	HGSOC	0.800	1.34	1.30	1.38	2.81E-88	0.800	1.34	1.30	1.38	2.81E-88	0.800	1.33	1.29	1.38	1.85E-66	0.802	1.34	1.24	1.44	4.66E-15	9.1E-01	0.805	1.50	1.33	1.70	9.04E-11	6.3E-02
Dareng 2024[4]	9p22.1	chr9:18530885-19530885	rs7851336	19030885	*SAXO1*	A/C	HGSOC	0.239	1.08	1.05	1.11	1.20E-08	0.238	1.08	1.05	1.11	1.20E-08	0.240	1.09	1.06	1.12	1.93E-09	0.227	1.04	0.97	1.12	2.63E-01	2.2E-01	0.233	0.96	0.86	1.08	5.35E-01	4.3E-02
Chen 2014[11]	9q22.33	chr9:98498959-99498959	rs1413299	98998959		T/G	CCOC	0.632	0.96	0.89	1.04	2.77E-01	0.631	1.00	0.98	1.03	7.90E-01	0.632	1.01	0.98	1.03	6.92E-01	0.628	0.99	0.93	1.05	7.65E-01	6.7E-01	0.631	1.01	0.90	1.12	9.22E-01	1.0E+00
Phelan 2017[2]	9q31.1	chr9:101680944-102680944	rs320203	102180944		C/A	MOC	0.150	0.81	0.74	0.88	4.69E-07	0.149	1.02	0.99	1.05	2.23E-01	0.149	1.02	0.98	1.05	3.59E-01	0.148	1.02	0.94	1.11	5.78E-01	8.7E-01	0.148	1.06	0.92	1.22	4.07E-01	5.6E-01
Kar 2016[3]**	9q31.1	chr9:103604421-104604422	rs2122577	104104422	*SMC2*	C/T	HGSOC	0.553	1.07	1.05	1.09	1.46E-09	0.556	1.07	1.05	1.09	1.46E-09	0.552	1.08	1.05	1.11	8.25E-10	0.574	1.05	0.99	1.11	9.99E-02	3.8E-01	0.564	0.98	0.89	1.08	6.90E-01	6.2E-02
Kuchenbaecker 2015[1]*	9q34.2	chr9:132779613-133779427	rs635634	133279427	*ABO*	C/T	HGSOC	0.196	1.12	1.09	1.15	1.59E-15	0.199	1.12	1.09	1.15	1.59E-15	0.194	1.13	1.09	1.16	7.11E-15	0.220	1.09	1.01	1.17	2.61E-02	3.4E-01	0.216	1.04	0.92	1.17	5.60E-01	2.0E-01
Pharoah 2013[7]	10p12.31	chr10:21032345-22032345	rs1243180	21532345	*MLLT10*	G/A	NMOC	0.321	1.09	1.06	1.11	1.86E-16	0.330	1.09	1.06	1.11	2.63E-13	0.325	1.09	1.06	1.12	3.66E-11	0.350	1.09	1.03	1.16	3.44E-03	8.9E-01	0.345	1.06	0.96	1.18	2.45E-01	6.5E-01
Chen 2014[11]	10p11.21	chr10:36380367-37380367	rs1192691	36880367		G/T	CCOC	0.941	0.89	0.76	1.04	1.39E-01	0.936	1.01	0.97	1.06	5.45E-01	0.940	1.02	0.97	1.08	3.91E-01	0.921	0.94	0.84	1.06	3.45E-01	2.2E-01	0.925	1.10	0.90	1.35	3.45E-01	4.9E-01
Phelan 2017[2]	10q24.33	chr10:103434544-104434543	rs7902587	103934543		C/T	LGSOC	0.100	1.26	1.16	1.37	1.43E-07	0.099	1.06	1.03	1.10	8.08E-04	0.099	1.06	1.02	1.10	6.04E-03	0.101	1.08	0.98	1.18	1.36E-01	7.4E-01	0.102	1.13	0.96	1.33	1.47E-01	4.5E-01
Kar 2016[3]**	11q12	chr11:61626500-62626500	rs7937840 [f]	62126500	*INCENP*	C/T	HGSOC	0.256	1.05	1.03	1.08	7.98E-05	0.256	1.05	1.03	1.08	7.98E-05	0.256	1.06	1.03	1.09	2.90E-05	0.256	1.01	0.94	1.08	8.31E-01	1.6E-01	0.257	1.03	0.92	1.15	6.43E-01	5.8E-01
Phelan 2017[2]*	12q24.31	chr12:120465921-121465921	rs7953249	120965921		A/G	HGSOC	0.416	1.08	1.05	1.10	6.69E-11	0.419	1.08	1.05	1.10	6.70E-11	0.414	1.07	1.05	1.10	9.83E-09	0.438	1.09	1.02	1.15	5.77E-03	7.5E-01	0.439	1.08	0.98	1.19	1.40E-01	9.6E-01
Kar 2016[3]**	15q26.1	chr15:90492097-91492099		90992099	*PRC1*	T/G	HGSOC	0.870	1.10	1.06	1.13	4.33E-08	0.869	1.10	1.06	1.13	4.33E-08	0.870	1.13	1.08	1.17	3.85E-10	0.867	0.98	0.90	1.07	6.53E-01	3.5E-03	0.868	1.02	0.89	1.18	7.60E-01	2.1E-01
Kuchenbaecker 2015[1]*	17q11.2	chr17:30339284-31339284	chr17:29181220:I	30839284		G/A	NMOC	0.732	1.05	1.03	1.07	9.26E-06	0.732	1.03	1.01	1.06	1.01E-02	0.731	1.05	1.02	1.08	1.08E-03	0.733	0.97	0.91	1.04	4.17E-01	4.3E-02	0.738	0.99	0.88	1.10	7.98E-01	3.0E-01
Pharoah 2013[7] & Shen 2013[12]	17q12	chr17:37243851-38240776	rs11651755	37740776	*HNF1B*	G/A	HGSOC	0.398	1.09	1.06	1.11	5.94E-14	0.398	1.09	1.06	1.11	5.94E-14	0.398	1.10	1.07	1.12	3.41E-13	0.398	1.05	0.99	1.11	1.18E-01	1.8E-01	0.412	1.09	0.99	1.20	9.25E-02	9.1E-01
Permuth-Wey 2013[13]	17q21.31	chr17:46059891-48445070		46109891	*PLEKH1*	A/G	HGSOC	0.226	1.13	1.10	1.16	1.68E-21	0.227	1.13	1.10	1.16	1.68E-21	0.225	1.12	1.09	1.16	1.19E-15	0.233	1.15	1.07	1.23	1.15E-04	6.0E-01	0.241	1.26	1.12	1.41	8.34E-05	5.8E-02
Pharoah 2013[7]	17q21.32	chr17:46059891-48445070		48395070	*SKAP1*	C/G	NMOC	0.274	1.12	1.10	1.15	5.20E-29	0.274	1.13	1.10	1.16	3.02E-23	0.274	1.14	1.11	1.17	8.64E-22	0.270	1.09	1.02	1.16	9.45E-03	2.2E-01	0.276	1.08	0.97	1.21	1.57E-01	3.7E-01
Phelan 2017[2]	18q11.2	chr18:23345888-24345888		23845888		C/T	LGSOC	0.370	1.17	1.10	1.24	2.00E-07	0.361	1.02	1.00	1.04	1.12E-01	0.362	1.01	0.98	1.04	4.43E-01	0.358	1.07	1.00	1.14	3.48E-02	9.7E-02	0.355	1.03	0.92	1.14	6.26E-01	7.7E-01
Bolton 2010[14] & Lawrenson 2016[15]	19p13.11	chr19:16779480-17779482	rs4808075	17279482	*BABAM1*	T/C	HGSOC	0.295	1.16	1.14	1.19	1.07E-36	0.296	1.16	1.14	1.19	1.07E-36	0.295	1.20	1.17	1.23	1.22E-42	0.303	0.99	0.93	1.05	7.29E-01	2.7E-08	0.297	1.12	1.00	1.24	4.35E-02	2.0E-01
Kar 2016[3]**	19p13	chr19:18917997-19917997	rs1469713	19417997		A/G	NMOC	0.328	1.03	1.01	1.05	9.88E-03	0.353	1.02	1.00	1.04	9.34E-02	0.352	1.03	1.00	1.06	2.76E-02	0.353	0.97	0.91	1.03	3.52E-01	8.7E-02	0.360	1.01	0.91	1.12	8.19E-01	7.6E-01
Kelemen 2015[5]	19q13.2	chr19:38748147-39748147	rs688187	39248147		C/T	MOC	0.302	0.68	0.64	0.73	1.03E-29	0.306	1.04	1.01	1.06	4.05E-03	0.302	1.04	1.01	1.07	3.33E-03	0.324	1.02	0.95	1.08	6.27E-01	4.9E-01	0.319	1.01	0.91	1.13	8.19E-01	6.3E-01
Dareng 2024[4]	21q22.12	chr21:34208097-35208100	rs2070368	34708100		C/T	NMOC	0.582	1.06	1.04	1.08	1.01E-09	0.597	1.05	1.02	1.07	7.02E-05	0.598	1.05	1.02	1.07	2.98E-04	0.591	1.05	0.99	1.11	1.12E-01	9.5E-01	0.589	1.03	0.93	1.14	5.78E-01	7.4E-01
Phelan 2017[2]	22q12.1	chr22:28038325-29038325	rs6005807 [f]	28538325	*TTC28*	T/C	HGSOC	0.904	1.07	1.03	1.11	9.75E-04	0.905	1.07	1.03	1.11	9.75E-04	0.904	1.10	1.05	1.14	1.81E-05	0.905	0.94	0.85	1.04	2.22E-01	5.4E-03	0.913	0.96	0.80	1.14	6.33E-01	1.4E-01

EAF = effect allele frequency. RR = relative risk (per effect allele). CI = confidence interval. P = P-value for association with HGSOC.

[a] Lookups are presented for the variant's association with its most strongly associated EOC histotype from the OCAC.

Histotypes: NMOC = non-mucinous, HGSOC = high-grade serous, LGSOC = low-grade serous, MOC = mucinous, CCOC = clear cell, EnOC = endometrioid.

Variants with HGSOC as the most strongly associated histotype in the OCAC data are highlighted in green.

[b] Lookups are also presented from the present HGSOC meta-analysis of OCAC, UKBB and *BRCA1/2* carriers, and its component parts (associations from OCAC and UKBB combined, associations from *BRCA1* carriers, and associations from *BRCA2* carriers).

[c] Positions are Genome Reference Consortium Human Build 38 (GRCh38/hg38). SNP = rsID of the most strongly associated variant with "best histotype".

[d] Alleles = other/effect alleles.

[e] P_{het} = P-value for heterogeneity between the general population OR (OCAC+UKBB meta-analysis) and the PV carrier HRs (*BRCA1* PV carriers or *BRCA2* PV carriers).

[f] Variants that were most strongly associated with HGSOC in OCAC that did not replicate ($P<5x10^{-8}$) in the OCAC+UKBB+CIMBA meta-analysis.

* Previously identified through meta-analysis of OCAC and CIMBA. ** Previously identified through multi-cancer meta-analysis.

Supplementary Table 4: Novel associations for HGSOC by major analytical unit (OCAC, UKBB, *BRCA1* carriers and *BRCA2* carriers) and association lookups for East Asian ancestry women in BioBank Japan.

Locus	SNP	Variant[a]	Alleles[b]	OCAC						UK Biobank						OCAC+UKBB meta-analysis					
				EAF[c]		OR	95% CI		P	EAF[c]		OR	95% CI		P	EAF[c]		OR	95% CI		P
				Controls	*Cases*					*Controls*	*Cases*					*Controls*	*Cases*				
5q11.2	rs528577783	chr5:53511827:C:G	C/G	0.00004	0.00018	5.39	1.54	18.87	8.47E-03	0.0018	0.0071	5.10	2.75	9.45	2.62E-05	0.0013	0.0006	5.15	2.96	8.96	6.53E-09
6p12.1	rs1013698558	chr6:53554782:A:T	A/T	0.00148	0.00255	2.31	1.70	3.16	1.21E-07	No association available[d]						0.0015	0.0025	2.31	1.70	3.16	1.21E-07
8p21.2	rs540569242	chr8:25522083:G:A	G/A	0.00005	0.00022	15.35	1.64	144.06	1.68E-02	0.0002	0.0021	33.85	10.72	106.89	4.05E-05	0.0001	0.0003	28.70	10.32	79.82	1.25E-10
9p24.1-p23	rs768719522	chr9:8333517:C:T	C/T	0.00015	0.00044	4.75	1.81	12.47	1.58E-03	0.0001	0.0003	10.13	0.41	249.53	2.66E-01	0.0001	0.0004	5.05	2.00	12.75	5.97E-04
16q22.1	rs6979	chr16:67657765:A:G	A/G	0.47242	0.49237	1.07	1.04	1.09	1.12E-06	0.4681	0.4853	1.07	0.98	1.18	1.48E-01	0.4694	0.4920	1.07	1.04	1.09	3.80E-07
17p13.1	rs143094271	chr17:7559785:G:A	A/G	0.97775	0.98318	1.23	1.12	1.35	2.03E-05	0.9790	0.9855	1.47	0.99	2.20	4.42E-02	0.9786	0.9833	1.24	1.13	1.36	4.59E-06
	rs78378222	chr17:7668434:T:G	G/T	0.98726	0.99183	1.37	1.20	1.57	4.88E-06	0.9878	0.9930	1.83	1.01	3.30	2.71E-02	0.9877	0.9919	1.39	1.22	1.59	9.52E-07
19q12	rs62107113	chr19:29797136:G:A	G/A	0.21852	0.22896	1.07	1.04	1.11	4.43E-06	0.2231	0.2446	1.13	1.01	1.27	3.33E-02	0.2217	0.2298	1.08	1.05	1.11	5.95E-07

Locus	SNP	Variant[a]	Alleles[b]	*BRCA1* carriers						*BRCA2* carriers						BioBank Japan					
				EAF[c]		HR	95% CI		P	EAF[c]		HR	95% CI		P	EAF[c]		OR	95% CI		P
				Unaffected	*Affected*					*Unaffected*	*Affected*					*Controls*	*Cases*				
5q11.2	rs528577783	chr5:53511827:C:G	C/G	No association available[d]						No association available[d]						No association available[d]					
6p12.1	rs1013698558	chr6:53554782:A:T	A/T	0.00158	0.00143	4.31	1.68	11.08	2.37E-03	0.001732	0.000966	0.79	0.19	3.38	7.52E-01	No association available[d]					
8p21.2	rs540569242	chr8:25522083:G:A	G/A	0.00005	0.00002	0.17	0.00	1926.62	7.09E-01	0.000081	0.000003	0.42	0.00	3122.71	8.48E-01	No association available[d]					
9p24.1-p23	rs768719522	chr9:8333517:C:T	C/T	0.00023	0.00038	36.90	4.00	340.13	1.45E-03	No association available[d]						No association available[d]					
16q22.1	rs6979	chr16:67657765:A:G	A/G	0.49687	0.51201	1.09	1.03	1.16	3.47E-03	0.493432	0.503581	0.98	0.89	1.09	7.18E-01	0.159	0.159	1.00	0.87	1.14	9.43E-01
17p13.1	rs143094271	chr17:7559785:G:A	A/G	0.98284	0.98381	1.49	1.19	1.86	5.74E-04	0.981023	0.979364	1.35	0.94	1.94	1.01E-01	0.99754	0.99819	1.38	0.52	3.67	5.19E-01
	rs78378222	chr17:7668434:T:G	G/T	0.99157	0.99276	1.59	1.16	2.19	4.27E-03	0.989710	0.987710	1.77	1.09	2.88	2.05E-02	No association available[d]					
19q12	rs62107113	chr19:29797136:G:A	G/A	0.22044	0.22279	1.11	1.03	1.19	4.24E-03	0.215660	0.193236	1.04	0.92	1.17	5.59E-01	No association available[d]					

EAF = effect allele frequency. OR = odds ratio per effect allele. HR = hazard ratio per effect allele. CI = confidence interval. P = P-value for association.

[a] Positions are Genome Reference Consortium Human Build 38 (GRCh38/hg38).

[b] Alleles = other/effect alleles.

[c] EAF in controls and cases in the OCAC and UK Biobank data. EAF in unaffected and affected *BRCA1*/*2* pathogenic variant carrier data.

[d] UKBB did not contribute associations for rs1013698558 (chr6:53554782:A:T).

[e] HRs were estimated but had large standard errors, resulting in wide CIs. These associations were used in the meta-analysis between OCAC, UKBB and CIMBA, but contributed little as the inverse variance weights were extremely small.

[f] No association summary statistics were available in BioBank Japan.

Supplementary Table 5: Further details for eight novel variants associated with HGSOC risk.

Locus	SNP rsID	Chromosome: position[a]	Gene	Nearest gene(s)	Gene description[b]	Phenoscanner[c]	Pheweb[c]	GTEx[d]	eQTLGen[e]
5q11.2	rs528577783	5:53511827		Intergenic between *FST* and *NDUFS4*	***FST***: Follistatin is a singlE-chain gonadal protein that specifically inhibits folliclE-stimulating hormone release. The single FST gene encodes two isoforms, FST317 and FST344 containing 317 and 344 amino acids respectively, resulting from alternative splicing of the precursor mRNA. In a study in which 37 candidate genes were tested for linkage and association with polycystic ovary syndrome (PCOS) or hyperandrogenemia in 150 families, evidence was found for linkage between PCOS and follistatin (PMID: 10411917).			*FST* expressed in breast (19.54), fallopian tube (17.48), cervix ectocervix (17.24), prostate (16.34), vagina (10.23), uterus (7.96), ovary (7.949), cervix endocervix (7.502), testis (6.682)<br><br>*NDUFS4* expressed in ovary (114.4), cervix ectocervix (99.11), uterus (94.69), breast (90.65), cervix endocervix (89.49), fallopian tube (86.80), vagina (84.60), prostate (70.82), testis (59.19)	635 cis-eQTL variants for *NDUFS4*
6p12.1	rs1013698558	6:53554782		Intergenic between *GCLC* and *KILH*	***GCLC***: GlutamatE-cysteine ligase, also known as gamma-glutamylcysteine synthetase is the first ratE-limiting enzyme of glutathione synthesis. The enzyme consists of two subunits, a heavy catalytic subunit and a light regulatory subunit. This locus encodes the catalytic subunit, while the regulatory subunit is derived from a different gene located on chromosome 1p22-p21. Mutations at this locus have been associated with hemolytic anemia due to deficiency of gamma-glutamylcysteine synthetase and susceptibility to myocardial infarction.			*GCLC* expressed in vagina (26.17), prostate (24.53), breast (16.85), cervix endocervix (14.57), uterus (13.14), fallopian tube (13.08), cervix ectocervix (12.20), ovary (11.91), testis (10.43)	314 cis-eQTL variants for *GCLC*
8p21.2	rs540569242	8:25522083	*LOC107986933* intronic	*CDCA2*	***CDCA2***: This gene encodes a targeting subunit of the cell-cycle associated protein, protein phosphatase 1, with a role in targeting this protein to chromatin during anaphase. These two proteins comprise a phosphatase complex that is involved in nuclear envelope reformation and regulation of the DNA damage response. The encoded protein may also play a role in cancer progression. Alternative splicing results in multiple transcript variants.			*CDCA2* expressed in testis (36.57)	308 cis-eQTL variants for *CDCA2*
9p24.1-p23	rs768719522	9:8333517	*PTPRD* intronic		***PTPRD***: The protein encoded by this gene is a member of the protein tyrosine phosphatase (PTP) family. PTPs are known to be signaling molecules that regulate a variety of cellular processes including cell growth, differentiation, mitotic cycle, and oncogenic transformation. This PTP contains an extracellular region, a single transmembrane segment and two tandem intracytoplasmic catalytic domains, and thus represents a receptor-type PTP. The extracellular region of this protein is composed of three Ig-like and eight fibronectin type III-like domains.			*PTPRD* expressed in ovary (19.44), cervix ectocervix (9.529), cervix endocervix (9.261)	
16q22.1	rs6979	16:67657765	*ACD* missense	*CARMIL2* 500b downstream variant	***ACD***: This gene encodes a protein that is involved in telomere function. This protein is one of six core proteins in the telosome/shelterin telomeric complex, which functions to maintain telomere length and to protect telomere ends. Through its interaction with other components, this protein plays a key role in the assembly and stabilization of this complex, and it mediates the access of telomerase to the telomere. Multiple transcript variants encoding different isoforms have been found for this gene. This gene, which is also referred to as TPP1, is distinct from the unrelated TPP1 gene on chromosome 11, which encodes tripeptidyl-peptidase I.<br><br>***CARMIL2***: This gene encodes a member of the CARMIL (capping protein, Arp2/3, myosin-I linker) family of proteins. The encoded protein interacts with and negatively regulates the heterodimeric capping protein and promotes cell migration. Reduced expression of this gene has been observed in human psoriasis patients. Mutations in this gene cause a human immunodeficiency syndrome characterized by smooth muscle tumors and impaired T-cell function.	Phelan, 2017, Nat Genet:<br>Invasive EOC (P=1.19E-5)<br>Serous invasive EOC (P=1.43E-4)<br>HGS EOC (P=8.59E-5)<br><br>Bilateral oophorectomy in UKBB (P=6.07E-3)		*ACD* expressed in testis (66.80), ovary (56.89), uterus (48.56), cervix ectocervix (46.32), cervix endocervix (45.52), fallopian tube (44.17), prostate (33.88), vagina (29.35), breast (26.88)	1,525 cis-eQTL variants for *ACD*<br><br>Trans-eQTL variants for *ACD*: 10 SNPs at 6p22 (MHC region)
17p13.1	rs143094271	17:7559785	*TNFSF12-TNFSF13* intronic<br><br>*TNFSF13* itronic		***TNFSF12-TNFSF13***: This gene encodes a member of the tumor necrosis factor superfamily. It encodes a hybrid protein composed of the cytoplasmic and transmembrane domains of family member 12 fused to the C-terminal domain of family member 13. The hybrid protein is membrane anchored and presents the receptor-binding domain of family member 13 at the cell surface. It stimulates cycling in T- and B-lymphoma cell lines.<br><br>***TNFSF13***: The protein encoded by this gene is a member of the tumor necrosis factor (TNF) ligand family. This protein is a ligand for TNFRSF17/BCMA, a member of the TNF receptor family. This protein and its receptor are both found to be important for B cell development. In vitro experiments suggested that this protein may be able to induce apoptosis through its interaction with other TNF receptor family proteins such as TNFRSF6/FAS and TNFRSF14/HVEM. Alternative splicing results in multiple transcript variants. Some transcripts that skip the last exon of the upstream gene (TNFSF12) and continue into the second exon of this gene have been identified; such read-through transcripts are contained in GeneID 407977, TNFSF12-TNFSF13.	Phelan, 2017, Nat Genet:<br>HGS EOC (P=2.87E-4)<br>Serous invasive EOC (P=1.17E-3)<br>Invasive EOC (P=7.57E-3)<br>Mucinous EOC (P=1.23E-2)<br>Low grade and borderline serous EOC (1.41E-2)	Uterine leiomyoma (P=1.6E-12)<br>Other non-epithelial cancer of skin (P=8.8E-11)<br>Benign neoplasm of uterus (P=1.9E-10)<br>Skin cancer (P=2.4E-9)	*TNFSF13* expressed in ovary (45.72), fallopian tube (45.00), cervix endocervix (44.89), prostate (38.85), cervix ectocervix (35.23), breast (31.11), uterus (28.56), vagina (27.14)<br><br>rs143094271-A eQTL for:<br>*TP53* subcutaneous adipose (NES = -0.40, P=0.000010)<br>*TNFSF12* whole blood (NES = -0.38, P=0.000028)<br>*TP53* skin not exposed to sun (NES = -0.28, P=0.000029)	rs143094271-A cis-eQTL for:<br>*ATP1B2* (z = 13.9964, P=1.6443E-44)<br>*TNFSF12* (z = -8.4597, P=2.6784E-17)<br>*TP53* (z = -7.7031, P=1.327E-14)<br>*AC113189.5* (z = -6.3433, P=2.2475E-10)<br>*NLGN2* (z = 5.8329, P=5.4505E-9)<br>*CD68* (z = 4.6564, P=0.0000032179)
	rs78378222	17:7668434	*TP53* 3' UTR variant		***TP53***: This gene encodes a tumor suppressor protein containing transcriptional activation, DNA binding, and oligomerization domains. The encoded protein responds to diverse cellular stresses to regulate expression of target genes, thereby inducing cell cycle arrest, apoptosis, senescence, DNA repair, or changes in metabolism. Mutations in this gene are associated with a variety of human cancers, including hereditary cancers such as Li-Fraumeni syndrome. Alternative splicing of this gene and the use of alternate promoters result in multiple transcript variants and isoforms. Additional isoforms have also been shown to result from the use of alternate translation initiation codons from identical transcript variants (PMIDs: 12032546, 20937277).	G allele associated with Glioma (OR=2.53, 95%CI:2.19-2.91 P=8.64E-38) and Glioblastoma (OR=2.63, P=5E-29) [PMID 28346443]<br><br>Basal cell carcinoma, per G allele: PMID 25855136 (OR=7.92 P=1E-20). PMID 21946351 (OR=2.16, P=2E-20). PMID 27539887 (OR=1.41, P=2E-10)	Uterine leiomyoma (P=5.1E-24)<br>Other non-epithelial cancer of skin (P=1.4E-23)<br>Skin cancer (P=1.7E-21)<br>Benign neoplasm of uterus (P=1.1E-20)<br>Benign neoplasm of skin (P=7.0E-9)<br>Cancer of brain and nervous system (P=1.3E-8)<br>Manlignant and unknown neoplasms of brain and nervous system (P=1.7E-8)<br>Benign neoplasm of colon (P=1.8E-8)<br>Cancer of brain (P=4.4E-8)	*TP53* expressed in ovary (32.43), cervix endocervix (29.50), uterus (29.50), cervix ectocervix (28.48), fallopian tube (28.16), vagina (25.29), breast (24.56), prostate (20.30), testis (14.83).<br><br>rs78378222-G eQTL for:<br>*TP53* skin sun exposed lower leg (NES = -0.56, P=3E-11)<br>*TP53* skin not sun exposed suprapubic (NES = -0.58, P=2.1E-10)<br>*TP53* adipose subcutaneous (NES = -0.77, P=8.7E-10)<br>*TP53* esophagus mucosa (NES = -0.73, P=5.2E-7)<br>*TP53* cells cultured fibroblasts (NES = 0.47, P=0.0000091)	rs78378222-G cis-eQTL for:<br>*ATP1B2* (z = 10.7227, P=7.973E-27)<br>*TP53* (z = -9.389, P=6.0458E-21)<br>*TNFSF12* (z = -6.5431, P=6.0216E-11)<br>*AC113189.5* (z = -4.9421, P=7.7246E-7)<br>*NLGN2* (z = 4.8149, P=0.0000014735)<br>*CD68* (z = 4.7485, P=0.0000020503)<br>*CLDN7* (z = -4.7423, P=0.000002112)<br><br>rs78378222-G trans-eQTL for:<br>*CD70* (chr19, z = -5.3818, P=7.3745E-8)<br>*AEN* (chr15, z = -4.9034, P=9.4096E-7)
19q12	rs62107113	19:29797136		*CCNE1*	***CCNE1***: The protein encoded by this gene belongs to the highly conserved cyclin family, whose members are characterized by a dramatic periodicity in protein abundance through the cell cycle. Cyclins function as regulators of CDK kinases. Different cyclins exhibit distinct expression and degradation patterns which contribute to the temporal coordination of each mitotic event. This cyclin forms a complex with and functions as a regulatory subunit of CDK2, whose activity is required for cell cycle G1/S transition. This protein accumulates at the G1-S phase boundary and is degraded as cells progress through S phase. Overexpression of this gene has been observed in many tumors, which results in chromosome instability, and thus may contribute to tumorigenesis. This protein was found to associate with, and be involved in, the phosphorylation of NPAT protein (nuclear protein mapped to the ATM locus), which participates in cell-cycle regulated histone gene expression and plays a critical role in promoting cell-cycle progression in the absence of pRB.	Phelan, 2017, Nat Genet:<br>Serous invasive EOC (P=1.26E-05)<br>HGS EOC (P=1.93E-05)<br>Invasive EOC (P=3.68E-04)		*CCNE1* expressed in testis (28.54)<br><br>rs62107113-A eQTL for:<br>*CCNE1* skin not sun exposed suprapubic (NES = 0.52, P=1.7E-30)<br>*CCNE1* skin sun exposed lower leg (NES = 0.44, P=1.2E-29)<br>*CCNE1* esophagus mucosa (NES = 0.30, P=5.5E-13)<br>*CCNE1* thyroid (NES = -0.22, P=0.000027)<br>*CCNE1* adipose subcutaneous (NES = -0.21, P=0.000040)<br>*URI1* skin sun exposed lower leg (NES = 0.11, P=0.000077)	rs62107113-A cis-QTL for:<br>C19orf12 (z = 9.6242, P=6.3197E-22)<br>*CCNE1* (z = -7.2611, P=3.8367E-13)<br><br>180 cis-eQTL variants for *CCNE1*

[a] Positions are Genome Reference Consortium Human Build 38 (GRCh38/hg38).

[b] Gene descriptions are taken from dbSNP[16].

[c] Phenoscanner[17-19] and PheWeb[20] are lookups of relevant associations for these variants/genes. PheWeb[20] associations were those that were genome-wide statistically significant from the UKBB using TOPMed imputation.

[d] GTEx[21] gene expression data is reported for human reproductive tissues with TPM (transcripts per million) > 5.

[e] eQTLGen[22,23] lookups for cis- and trans-eQTLs for variants and genes were performed.

Supplementary Table 6: List of credible causal variants (CCVs) at each novel region.

Locus	Region	Variant[a]	Lead	Alleles		EAF	RR	95% CI		P
				Other	*Effect*					
5q11.2	5:53011827..54011827	chr5:53511827:C:G	**Yes**	C	G	0.0013	5.15	2.96	8.96	6.60E-09
6p12.1	6:53054782..54054782	chr6:53554782:A:T	**Yes**	A	T	0.0015	2.35	1.76	3.14	6.86E-09
8p21.2	8:25022083..26022083	chr8:25522083:G:A	**Yes**	G	A	0.0001	25.60	9.32	70.31	3.15E-10
9p24.1-p23	9:7833517..8833517	chr9:8333517:C:T	**Yes**	C	T	0.0002	10.04	4.38	22.99	4.92E-08
16q22.1	16:67157765..68157765	chr16:67657765:A:G	**Yes**	A	G	0.4750	1.07	1.04	1.09	2.30E-08
		chr16:67819522:T:C	No	T	C	0.4478	1.06	1.04	1.08	1.96E-07
		chr16:67829548:T:C	No	T	C	0.4488	1.06	1.04	1.08	3.02E-07
		chr16:67970916:T:C	No	T	C	0.4732	1.06	1.03	1.08	1.60E-06
		chr16:67983328:A:G	No	A	G	0.4724	1.06	1.03	1.08	1.54E-06
17p13.1	17:7168434..8168434	chr17:7559785:G:A	No	A	G	0.9793	1.28	1.18	1.39	7.61E-09
		chr17:7668434:T:G	**Yes**	G	T	0.9883	1.44	1.28	1.62	1.76E-09
		chr17:7675353:C:T	No	T	C	0.9806	1.27	1.16	1.38	7.67E-08
19q12	19:29297136..30297136	chr19:29782616:G:A	No	G	A	0.2096	1.07	1.04	1.10	9.09E-07
		chr19:29794641:C:T	No	C	T	0.3275	1.06	1.04	1.09	4.27E-07
		chr19:29795338:A:G	No	A	G	0.3295	1.06	1.04	1.09	6.42E-07
		chr19:29795915:T:C	No	T	C	0.3291	1.06	1.04	1.09	4.38E-07
		chr19:29797136:G:A	**Yes**	G	A	0.2214	1.08	1.05	1.11	1.22E-08
		chr19:29797270:G:A	No	G	A	0.3291	1.06	1.04	1.09	4.26E-07
		chr19:29797581:C:T	No	C	T	0.3299	1.06	1.04	1.09	3.86E-07
		chr19:29797638:T:C	No	T	C	0.3296	1.06	1.04	1.09	4.27E-07
		chr19:29798723:G:A	No	G	A	0.3272	1.06	1.04	1.09	3.09E-07
		chr19:29798872:C:T	No	C	T	0.3275	1.06	1.04	1.09	2.56E-07
		chr19:29799450:G:C	No	G	C	0.3299	1.06	1.04	1.09	2.14E-07
		chr19:29799904:A:G	No	A	G	0.3305	1.06	1.04	1.09	1.93E-07
		chr19:29799983:A:G	No	A	G	0.3301	1.06	1.04	1.09	3.09E-07
		chr19:29802360:C:T	No	C	T	0.3299	1.06	1.04	1.09	2.13E-07
		chr19:29802555:G:A	No	G	A	0.3299	1.06	1.04	1.09	1.87E-07
		chr19:29803809:G:A	No	G	A	0.3300	1.06	1.04	1.09	1.81E-07
		chr19:29804084:G:T	No	G	T	0.3284	1.06	1.04	1.09	2.10E-07
		chr19:29804399:G:A	No	G	A	0.3284	1.06	1.04	1.09	1.42E-07
		chr19:29805946:T:C	No	T	C	0.3285	1.06	1.04	1.09	2.17E-07
		chr19:29806609:A:G	No	A	G	0.3290	1.06	1.04	1.09	2.96E-07
		chr19:29806670:G:A	No	G	A	0.3289	1.06	1.04	1.09	1.64E-07
		chr19:29807012:G:C	No	G	C	0.3290	1.06	1.04	1.09	2.13E-07
		chr19:29807893:A:G	No	A	G	0.3290	1.06	1.04	1.09	2.30E-07
		chr19:29808727:A:C	No	A	C	0.3291	1.06	1.04	1.09	2.82E-07
		chr19:29808889:A:T	No	A	T	0.3287	1.06	1.04	1.09	2.04E-07
		chr19:29809110:G:A	No	G	A	0.3283	1.06	1.04	1.09	2.51E-07
		chr19:29828296:G:A	No	G	A	0.3161	1.06	1.04	1.09	1.75E-07
		chr19:29828985:C:T	No	C	T	0.3159	1.07	1.04	1.09	1.52E-07
		chr19:29830957:A:AT	No	A	AT	0.3617	1.06	1.03	1.08	1.02E-06
		chr19:29834425:G:T	No	G	T	0.3198	1.06	1.04	1.09	1.19E-06
		chr19:29834472:G:A	No	G	A	0.3199	1.06	1.04	1.09	1.00E-06
		chr19:29834775:G:A	No	G	A	0.3198	1.06	1.04	1.09	1.04E-06
		chr19:29849787:A:G	No	A	G	0.2629	1.07	1.04	1.09	1.90E-07
		chr19:29849817:G:A	No	G	A	0.2645	1.07	1.04	1.09	1.62E-07
		chr19:29850957:G:A	No	G	A	0.2626	1.07	1.04	1.09	2.49E-07
		chr19:29851772:A:T	No	A	T	0.2622	1.07	1.04	1.09	2.94E-07
		chr19:29852593:G:A	No	G	A	0.2631	1.07	1.04	1.09	1.91E-07
		chr19:29852759:A:C	No	A	C	0.2618	1.07	1.04	1.09	2.72E-07
		chr19:29852760:G:C	No	G	C	0.2618	1.07	1.04	1.09	2.73E-07
		chr19:29853629:C:T	No	C	T	0.2624	1.07	1.04	1.09	1.99E-07

[a] Positions are Genome Reference Consortium Human Build 38 (GRCh38/hg38).

EAF = effect allele frequency. RR = relative risk per effect allele. CI = confidence interval. P = P-value for association.

Lead variants for each set of CCVs are highlighted in green.

These data are presented graphically in Supplementary Figures 42-48.

Supplementary Table 7: Risk reclassification based on different polygenic scores for *BRCA2* carriers.

		**PGM_{64518}**		**PGM_{400}**	
	Lifetime risk	<10%	≥10%	<10%	≥10%
**PGM_{36}**	<10%	10643	471	10740	374
	≥10%	157	367	188	336
	Total reclassification	628 (5.40%)		562 (4.83%)	

Lifetime risks were categorized as lower risk (lifetime risk < 10%) or higher risk (lifetime risk ≥ 10%).

The PGM_{64518} and PGM_{400} were compared with PGM_{36} that is currently used in the CanRisk prediction algorithm[4,24].

SUPPLEMENTARY DATA LEGENDS

Supplementary Data 1: List of variants contributing to the polygenic models (PGMs).

Positions are Genome Reference Consortium Human Build 38 (GRCh38/hg38).

Weights are given for five PGMs and are per effect allele. Instances in which weights are not present (NA) means that that variant does not contribute to that PGM. The weights are taken from the OCAC, UKBB and CIMBA meta-analysis effect size estimates with the hyperparameters applied.

Four of the eight variants found in the discovery GWAS were directly included in the 64,518 variant PGM (rs6979 chr16:67657765, rs143094271 chr17:7559785, rs78378222 chr17:7668434 and rs62107113 chr19:29797136, highlighted in green in the table).

SUPPLEMENTARY FIGURE LEGENDS

Supplementary Figures 1-41: Regional association plots for genomic regions previously associated with any EOC histotype. The regions are described in Supplementary Table 3. Regions chr4:164487569-166700821 (Supplementary Figure 11) and chr8:126575659-129029685 (Supplementary Figure 19) have been truncated to chr4:164594195-166594195 and chr8:126802672-128802672, respectively. The chr17:46059891-48445070 region has been displayed across two figures, as chr17:45109891-47109891 and chr17:47395070-49395070 (Supplementary Figures 34-35).

Supplementary Figures 42-48: Regional association plots for seven novel genomic regions associated with HGSOC. Credible causal variants (CCVs) are highlighted in green. Lists of CCVs are presented in Supplementary Table 6.

Supplementary Figure 49: European ancestry principal components plots of the top two principal components for women participating in (a) OCAC, and (b) CIMBA.

SUPPLEMENTARY REFERENCES

1 Kuchenbaecker, K. B. *et al.* Identification of six new susceptibility loci for invasive epithelial ovarian cancer. *Nat Genet* **47**, 164-171 (2015). https://doi.org/10.1038/ng.3185

2 Phelan, C. M. *et al.* Identification of 12 new susceptibility loci for different histotypes of epithelial ovarian cancer. *Nat Genet* **49**, 680-691 (2017). https://doi.org/10.1038/ng.3826

3 Kar, S. P. *et al.* Genome-Wide Meta-Analyses of Breast, Ovarian, and Prostate Cancer Association Studies Identify Multiple New Susceptibility Loci Shared by at Least Two Cancer Types. *Cancer Discov* **6**, 1052-1067 (2016). https://doi.org/10.1158/2159-8290.CD-15-1227

4 Dareng, E. O. *et al.* Integrative multi-omics analyses to identify the genetic and functional mechanisms underlying ovarian cancer risk regions. *Am J Hum Genet* **111**, 1061-1083 (2024). https://doi.org/10.1016/j.ajhg.2024.04.011

5 Kelemen, L. E. *et al.* Genome-wide significant risk associations for mucinous ovarian carcinoma. *Nat Genet* **47**, 888-897 (2015). https://doi.org/10.1038/ng.3336

6 Goode, E. L. *et al.* A genome-wide association study identifies susceptibility loci for ovarian cancer at 2q31 and 8q24. *Nat Genet* **42**, 874-879 (2010). https://doi.org/10.1038/ng.668

7 Pharoah, P. D. *et al.* GWAS meta-analysis and replication identifies three new susceptibility loci for ovarian cancer. *Nat Genet* **45**, 362-370, 370e361-362 (2013). https://doi.org/10.1038/ng.2564

8 Couch, F. J. *et al.* Genome-wide association study in BRCA1 mutation carriers identifies novel loci associated with breast and ovarian cancer risk. *PLoS Genet* **9**, e1003212 (2013). https://doi.org/10.1371/journal.pgen.1003212

9 Bojesen, S. E. *et al.* Multiple independent variants at the TERT locus are associated with telomere length and risks of breast and ovarian cancer. *Nat Genet* **45**, 371-384, 384e371-372 (2013). https://doi.org/10.1038/ng.2566

10 Song, H. *et al.* A genome-wide association study identifies a new ovarian cancer susceptibility locus on 9p22.2. *Nat Genet* **41**, 996-1000 (2009). https://doi.org/10.1038/ng.424

11 Chen, K. *et al.* Genome-wide association study identifies new susceptibility loci for epithelial ovarian cancer in Han Chinese women. *Nat Commun* **5**, 4682 (2014). https://doi.org/10.1038/ncomms5682

12 Shen, H. *et al.* Epigenetic analysis leads to identification of HNF1B as a subtype-specific susceptibility gene for ovarian cancer. *Nat Commun* **4**, 1628 (2013). https://doi.org/10.1038/ncomms2629

13 Permuth-Wey, J. *et al.* Identification and molecular characterization of a new ovarian cancer susceptibility locus at 17q21.31. *Nat Commun* **4**, 1627 (2013). https://doi.org/10.1038/ncomms2613

14 Bolton, K. L. *et al.* Common variants at 19p13 are associated with susceptibility to ovarian cancer. *Nat Genet* **42**, 880-884 (2010). https://doi.org/10.1038/ng.666

15 Lawrenson, K. *et al.* Functional mechanisms underlying pleiotropic risk alleles at the 19p13.1 breast-ovarian cancer susceptibility locus. *Nat Commun* **7**, 12675 (2016). https://doi.org/10.1038/ncomms12675

16 Sherry, S. T., Ward, M. & Sirotkin, K. dbSNP-database for single nucleotide polymorphisms and other classes of minor genetic variation. *Genome Res* **9**, 677-679 (1999).

17 Staley, J. R. *et al.* PhenoScanner: a database of human genotype-phenotype associations. *Bioinformatics* **32**, 3207-3209 (2016). https://doi.org/10.1093/bioinformatics/btw373

18 Kamat, M. A. *et al.* PhenoScanner V2: an expanded tool for searching human genotype-phenotype associations. *Bioinformatics* **35**, 4851-4853 (2019). https://doi.org/10.1093/bioinformatics/btz469

19 PhenoScanner. *PhenoScanner V2: A database of human genotype-phenotype associations*, <http://www.phenoscanner.medschl.cam.ac.uk/> (2023).

20 PheWeb. *PheWeb*, <https://pheweb.org/UKB-TOPMed/> (2023).

21 GTEx. *Genotype-Tissue Expression project*, <https://gtexportal.org/home/> (2023).

22 Vosa, U. *et al.* Large-scale cis- and trans-eQTL analyses identify thousands of genetic loci and polygenic scores that regulate blood gene expression. *Nat Genet* **53**, 1300-1310 (2021). https://doi.org/10.1038/s41588-021-00913-z

23 eQTLGen. *eQTLGen*, <https://www.eqtlgen.org/> (2023).

24 Lee, A. *et al.* Comprehensive epithelial tubo-ovarian cancer risk prediction model incorporating genetic and epidemiological risk factors. *J Med Genet* **59**, 632-643 (2022). https://doi.org/10.1136/jmedgenet-2021-107904

PRISMA 2020 Checklist

Section and Topic	Item #	Checklist item	Location where item is reported
**TITLE**			
Title	1	Identify the report as a systematic review.	N/A
**ABSTRACT**			
Abstract	2	See the PRISMA 2020 for Abstracts checklist.	PRISMA for Abstracts (Supplementary Information)
**INTRODUCTION**			
Rationale	3	Describe the rationale for the review in the context of existing knowledge.	N/A
Objectives	4	Provide an explicit statement of the objective(s) or question(s) the review addresses.	N/A
**METHODS**			
Eligibility criteria	5	Specify the inclusion and exclusion criteria for the review and how studies were grouped for the syntheses.	N/A
Information sources	6	Specify all databases, registers, websites, organisations, reference lists and other sources searched or consulted to identify studies. Specify the date when each source was last searched or consulted.	N/A
Search strategy	7	Present the full search strategies for all databases, registers and websites, including any filters and limits used.	N/A
Selection process	8	Specify the methods used to decide whether a study met the inclusion criteria of the review, including how many reviewers screened each record and each report retrieved, whether they worked independently, and if applicable, details of automation tools used in the process.	N/A
Data collection process	9	Specify the methods used to collect data from reports, including how many reviewers collected data from each report, whether they worked independently, any processes for obtaining or confirming data from study investigators, and if applicable, details of automation tools used in the process.	N/A
Data items	10a	List and define all outcomes for which data were sought. Specify whether all results that were compatible with each outcome domain in each study were sought (e.g. for all measures, time points, analyses), and if not, the methods used to decide which results to collect.	N/A
	10b	List and define all other variables for which data were sought (e.g. participant and intervention characteristics, funding sources). Describe any assumptions made about any missing or unclear information.	N/A
Study risk of bias assessment	11	Specify the methods used to assess risk of bias in the included studies, including details of the tool(s) used, how many reviewers assessed each study and whether they worked independently, and if applicable, details of automation tools used in the process.	N/A
Effect measures	12	Specify for each outcome the effect measure(s) (e.g. risk ratio, mean difference) used in the synthesis or presentation of results.	p35-p38
Synthesis methods	13a	Describe the processes used to decide which studies were eligible for each synthesis (e.g. tabulating the study intervention characteristics and comparing against the planned groups for each synthesis (item #5)).	N/A
	13b	Describe any methods required to prepare the data for presentation or synthesis, such as handling of missing summary statistics, or data conversions.	p38
	13c	Describe any methods used to tabulate or visually display results of individual studies and syntheses.	p21
	13d	Describe any methods used to synthesize results and provide a rationale for the choice(s). If meta-analysis was performed, describe the model(s), method(s) to identify the presence and extent of statistical heterogeneity, and software package(s) used.	p35, p37-p38
	13e	Describe any methods used to explore possible causes of heterogeneity among study results (e.g. subgroup analysis, meta-regression).	N/A

PRISMA 2020 Checklist

Section and Topic	Item #	Checklist item	Location where item is reported
	13f	Describe any sensitivity analyses conducted to assess robustness of the synthesized results.	p38-p39
Reporting bias assessment	14	Describe any methods used to assess risk of bias due to missing results in a synthesis (arising from reporting biases).	N/A
Certainty assessment	15	Describe any methods used to assess certainty (or confidence) in the body of evidence for an outcome.	p38-p39
**RESULTS**			
Study selection	16a	Describe the results of the search and selection process, from the number of records identified in the search to the number of studies included in the review, ideally using a flow diagram.	N/A
	16b	Cite studies that might appear to meet the inclusion criteria, but which were excluded, and explain why they were excluded.	N/A
Study characteristics	17	Cite each included study and present its characteristics.	p20, Table 1, Supplementary Tables 1-2
Risk of bias in studies	18	Present assessments of risk of bias for each included study.	p21-p22
Results of individual studies	19	For all outcomes, present, for each study: (a) summary statistics for each group (where appropriate) and (b) an effect estimate and its precision (e.g. confidence/credible interval), ideally using structured tables or plots.	Supplementary Tables 1-2, Supplementary Table 4
Results of syntheses	20a	For each synthesis, briefly summarise the characteristics and risk of bias among contributing studies.	N/A
	20b	Present results of all statistical syntheses conducted. If meta-analysis was done, present for each the summary estimate and its precision (e.g. confidence/credible interval) and measures of statistical heterogeneity. If comparing groups, describe the direction of the effect.	p21-p22, Table 2, Supplementary Table 4
	20c	Present results of all investigations of possible causes of heterogeneity among study results.	p21-p22, Table 2, Supplementary Table 4
	20d	Present results of all sensitivity analyses conducted to assess the robustness of the synthesized results.	p21-p22, Table 2, Supplementary Table 4
Reporting biases	21	Present assessments of risk of bias due to missing results (arising from reporting biases) for each synthesis assessed.	N/A
Certainty of evidence	22	Present assessments of certainty (or confidence) in the body of evidence for each outcome assessed.	p21-p22, Table 2
**DISCUSSION**			
Discussion	23a	Provide a general interpretation of the results in the context of other evidence.	p24-p29

PRISMA 2020 Checklist

Section and Topic	Item #	Checklist item	Location where item is reported
	23b	Discuss any limitations of the evidence included in the review.	N/A
	23c	Discuss any limitations of the review processes used.	N/A
	23d	Discuss implications of the results for practice, policy, and future research.	p32
**OTHER INFORMATION**			
Registration and protocol	24a	Provide registration information for the review, including register name and registration number, or state that the review was not registered.	N/A
	24b	Indicate where the review protocol can be accessed, or state that a protocol was not prepared.	N/A
	24c	Describe and explain any amendments to information provided at registration or in the protocol.	N/A
Support	25	Describe sources of financial or non-financial support for the review, and the role of the funders or sponsors in the review.	p48-p60
Competing interests	26	Declare any competing interests of review authors.	p73-p74
Availability of data, code and other materials	27	Report which of the following are publicly available and where they can be found: template data collection forms; data extracted from included studies; data used for all analyses; analytic code; any other materials used in the review.	p47

From: Page MJ, McKenzie JE, Bossuyt PM, Boutron I, Hoffmann TC, Mulrow CD, et al. The PRISMA 2020 statement: an updated guideline for reporting systematic reviews. BMJ 2021;372:n71. doi: 10.1136/bmj.n71.
This work is licensed under CC BY 4.0. To view a copy of this license, visit https://creativecommons.org/licenses/by/4.0/

PRISMA 2020 for Abstracts Checklist

Section and Topic	Item #	Checklist item	Reported (Yes/No)
**TITLE**			
Title	1	Identify the report as a systematic review.	N/A
**BACKGROUND**			
Objectives	2	Provide an explicit statement of the main objective(s) or question(s) the review addresses.	N/A
**METHODS**			
Eligibility criteria	3	Specify the inclusion and exclusion criteria for the review.	N/A
Information sources	4	Specify the information sources (e.g. databases, registers) used to identify studies and the date when each was last searched.	N/A
Risk of bias	5	Specify the methods used to assess risk of bias in the included studies.	N/A
Synthesis of results	6	Specify the methods used to present and synthesise results.	Yes
**RESULTS**			
Included studies	7	Give the total number of included studies and participants and summarise relevant characteristics of studies.	N/A
Synthesis of results	8	Present results for main outcomes, preferably indicating the number of included studies and participants for each. If meta-analysis was done, report the summary estimate and confidence/credible interval. If comparing groups, indicate the direction of the effect (i.e. which group is favoured).	Yes
**DISCUSSION**			
Limitations of evidence	9	Provide a brief summary of the limitations of the evidence included in the review (e.g. study risk of bias, inconsistency and imprecision).	N/A
Interpretation	10	Provide a general interpretation of the results and important implications.	Yes
**OTHER**			
Funding	11	Specify the primary source of funding for the review.	N/A
Registration	12	Provide the register name and registration number.	N/A

From: Page MJ, McKenzie JE, Bossuyt PM, Boutron I, Hoffmann TC, Mulrow CD, et al. The PRISMA 2020 statement: an updated guideline for reporting systematic reviews. BMJ 2021;372:n71. doi: 10.1136/bmj.n71. This work is licensed under CC BY 4.0. To view a copy of this license, visit https://creativecommons.org/licenses/by/4.0/

Supplementary Figure 1: Regional association plot for previously identified ovarian cancer region **chr1:21641722-22641722**

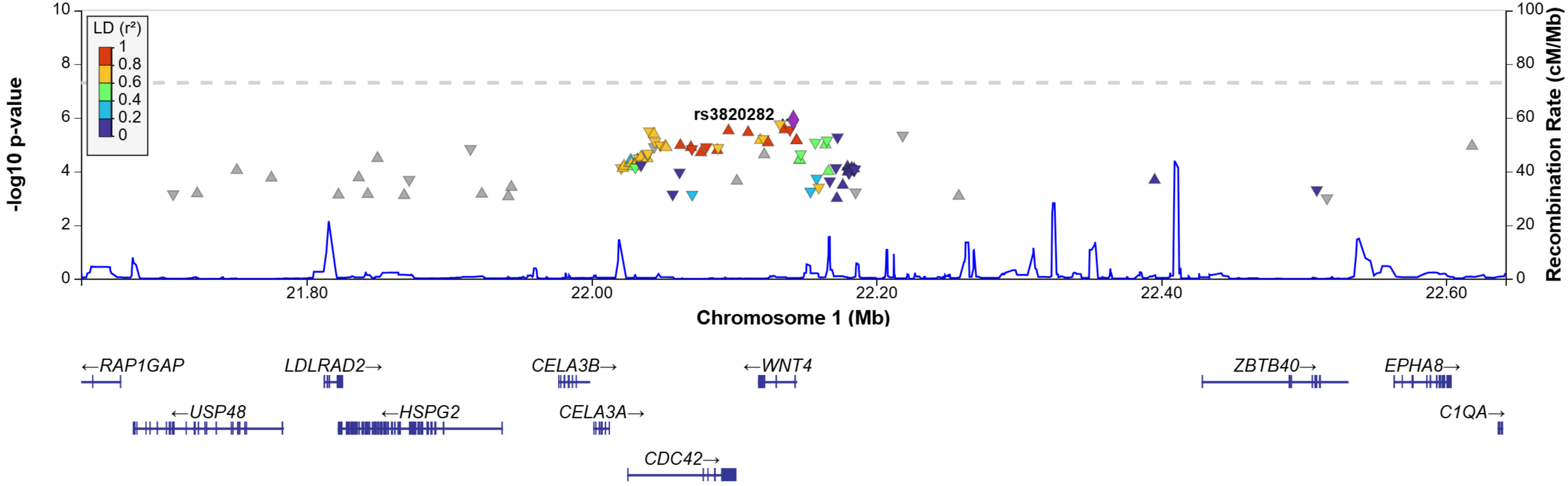

Supplementary Figure 2: Regional association plot for previously identified ovarian cancer region **chr1:37116521-38116450**

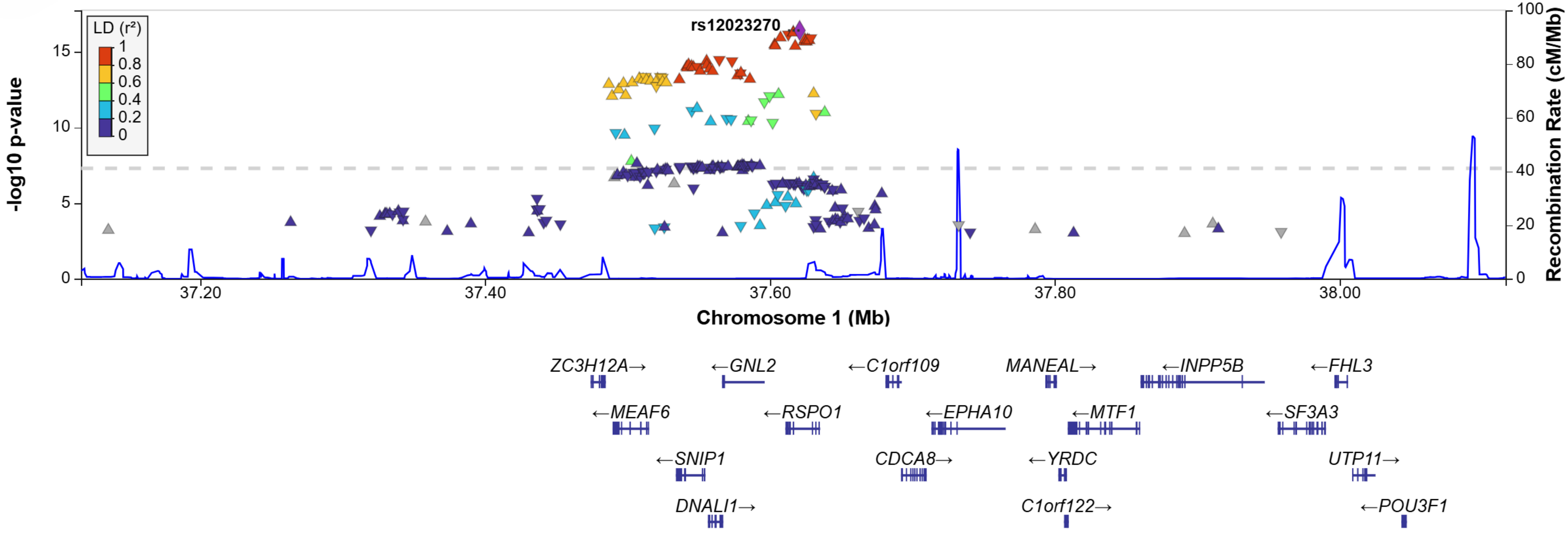

Supplementary Figure 3: Regional association plot for previously identified ovarian cancer region **chr2:110525257-111658369**

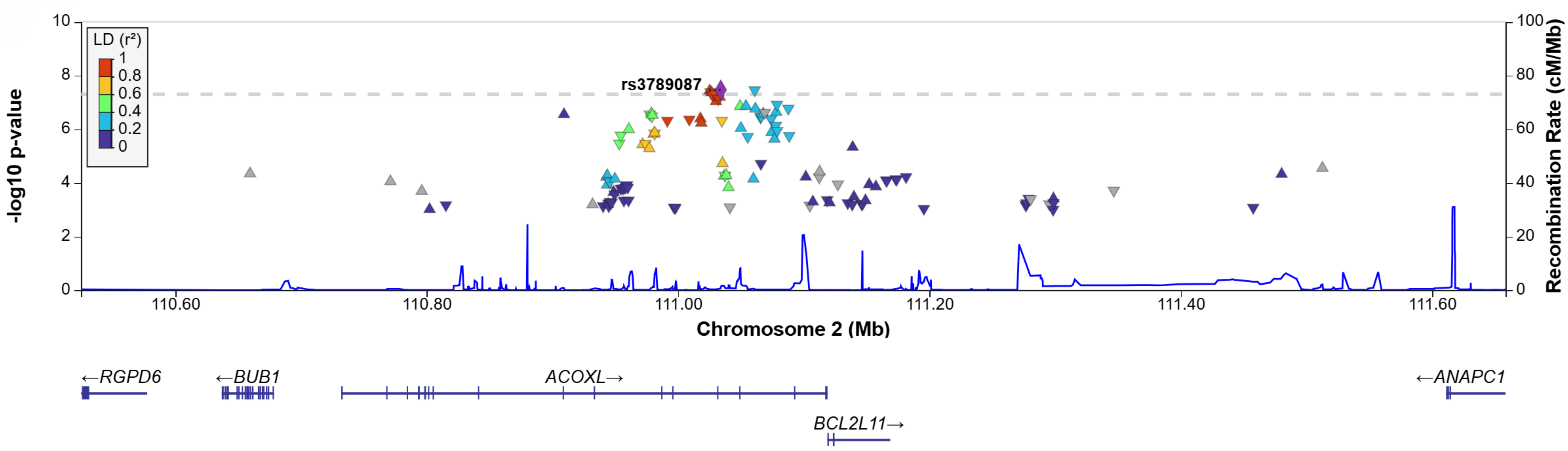

Supplementary Figure 4: Regional association plot for previously identified ovarian cancer region **chr2:112716387-113721787**

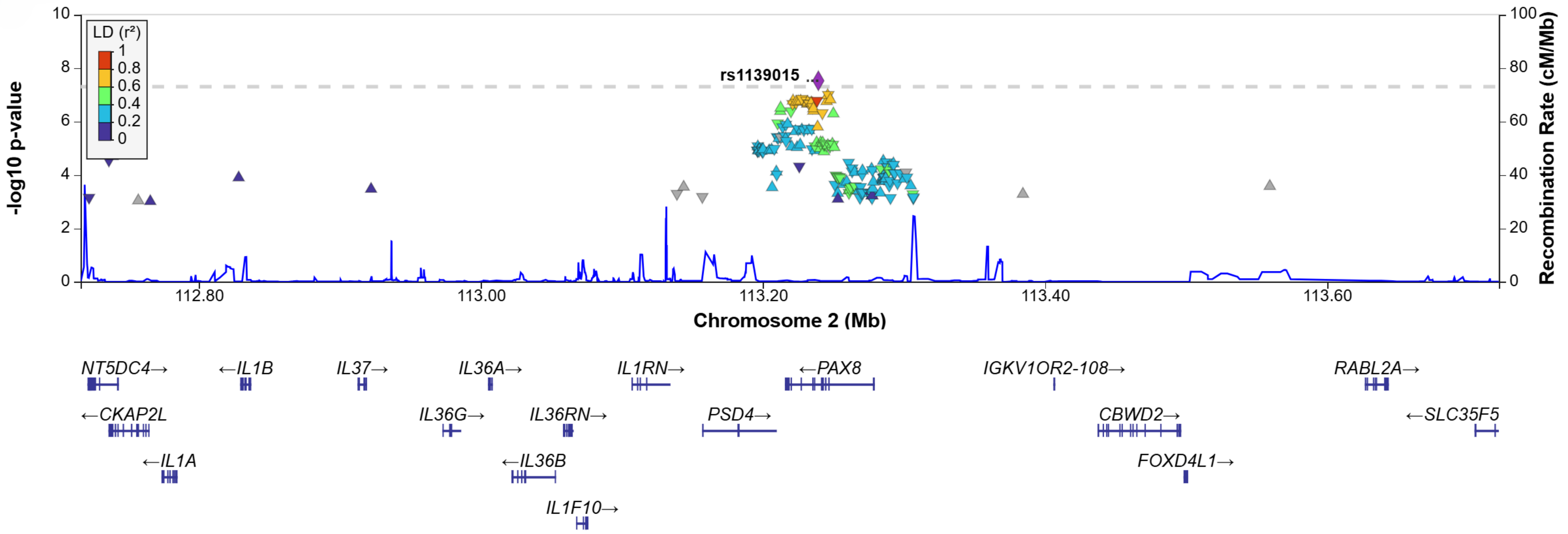

Supplementary Figure 5: Regional association plot for previously identified ovarian cancer region **chr2:119888925-120888925**

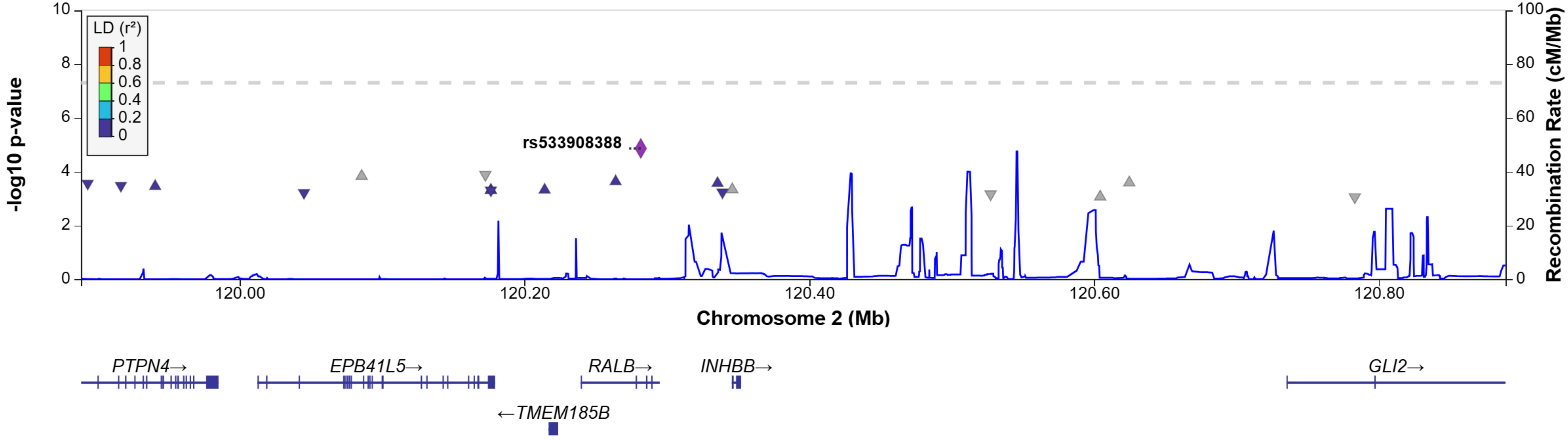

Supplementary Figure 6: Regional association plot for previously identified ovarian cancer region **chr2:175674850-177143894**

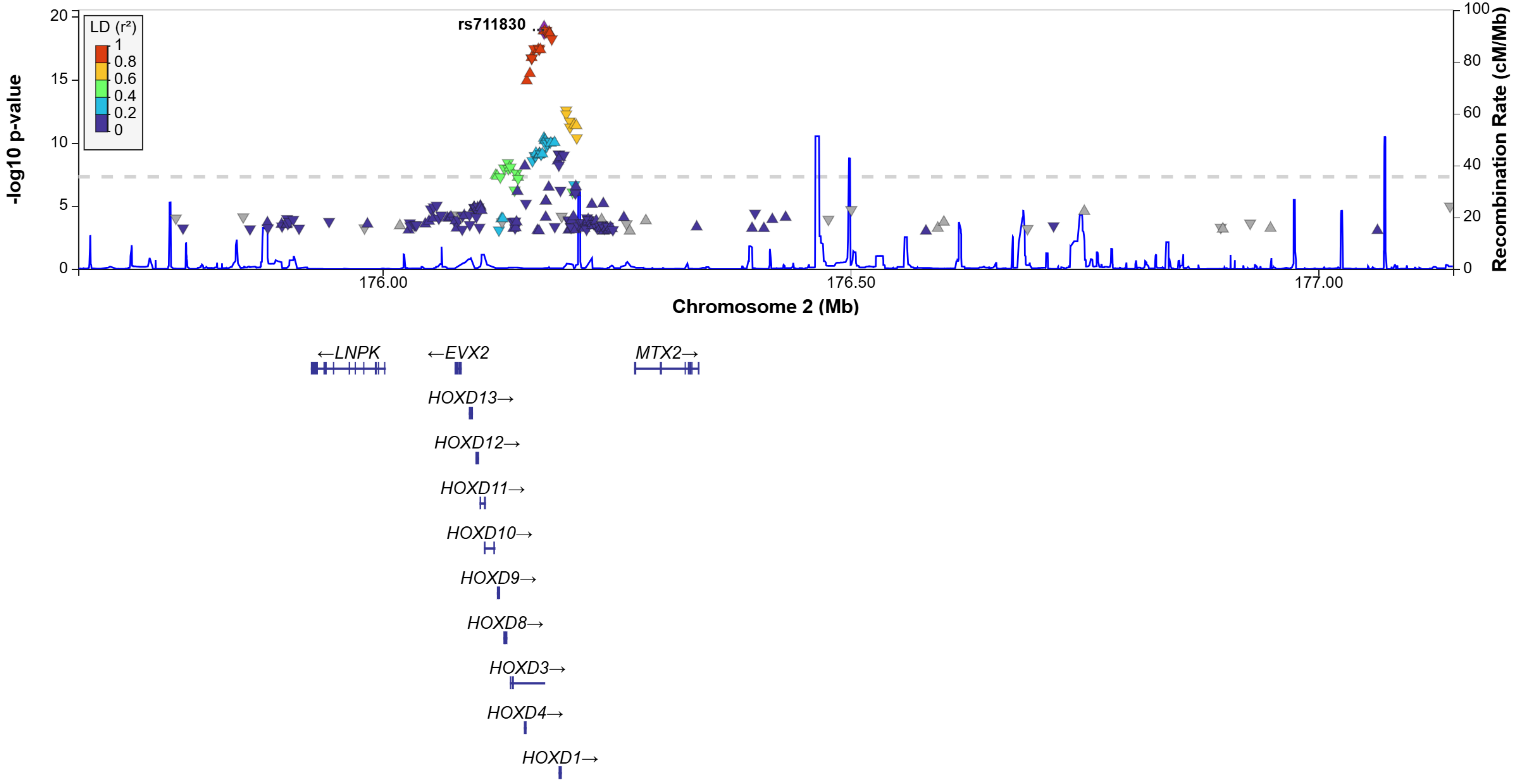

Supplementary Figure 7: Regional association plot for previously identified ovarian cancer region **chr3:138630701-139630701**

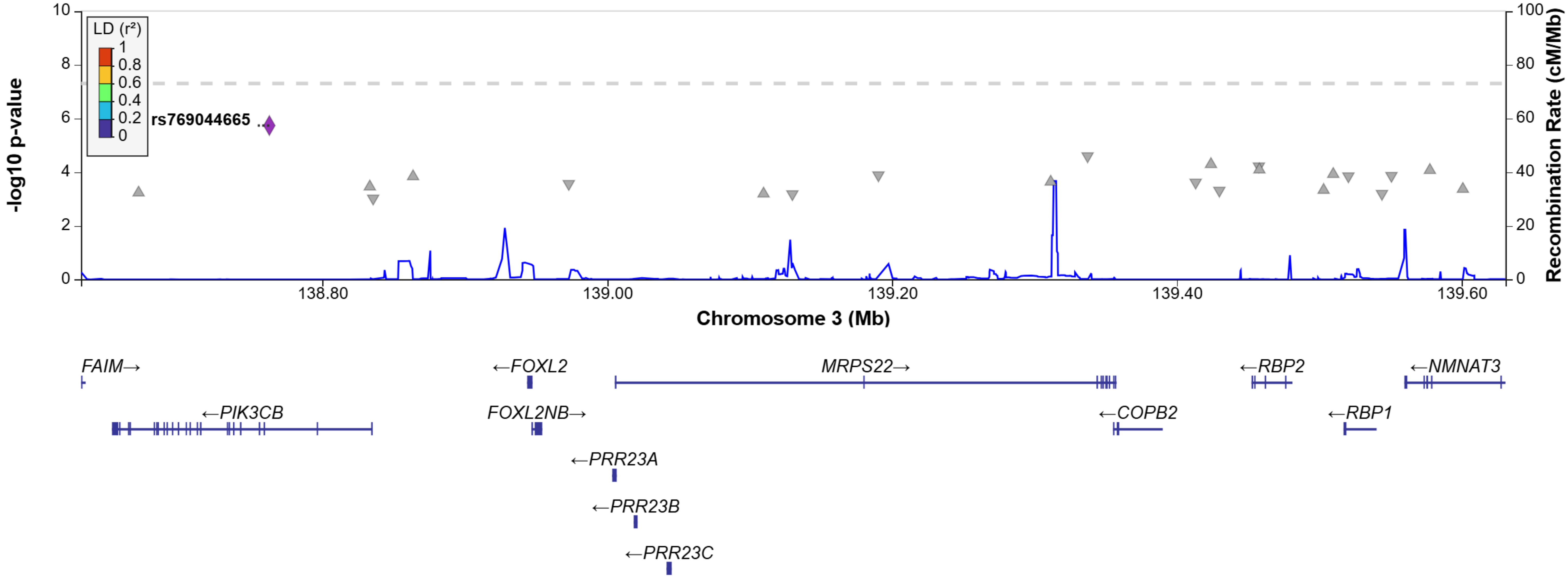

Supplementary Figure 8: Regional association plot for previously identified ovarian cancer region **chr3:156184698-157184698**

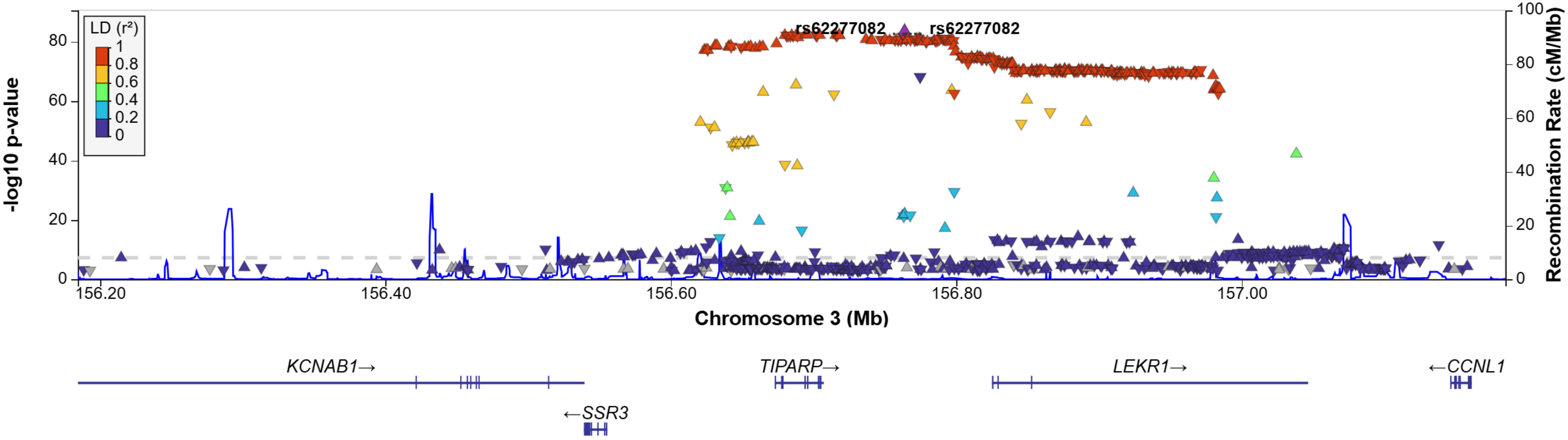

Supplementary Figure 9: Regional association plot for previously identified ovarian cancer region **chr3:190314093-191314093**

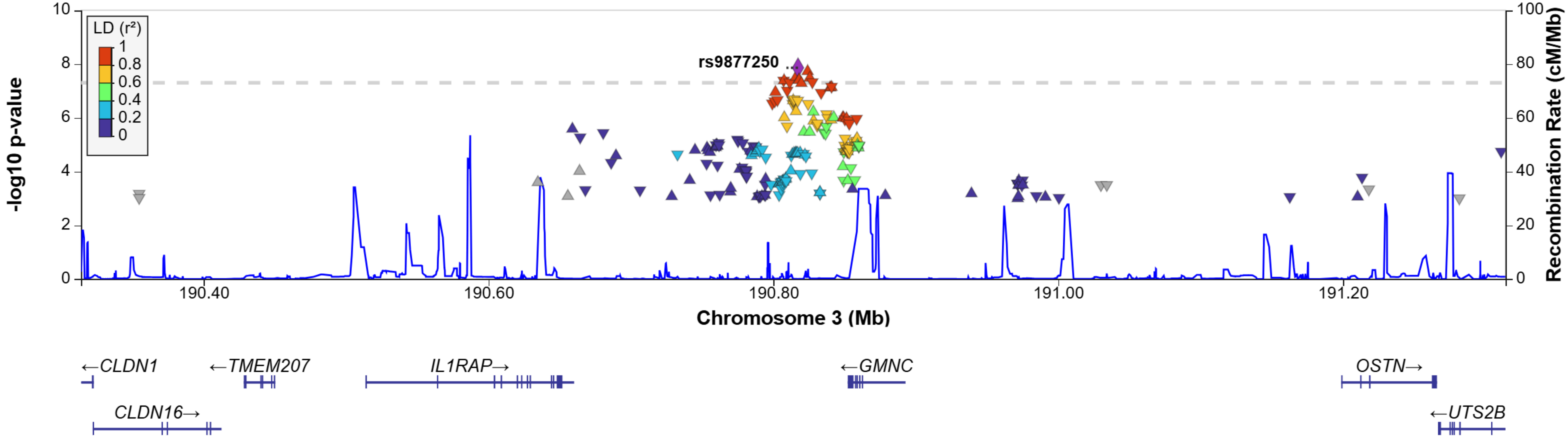

Supplementary Figure 10: Regional association plot for previously identified ovarian cancer region **chr4:118546750-119546750**

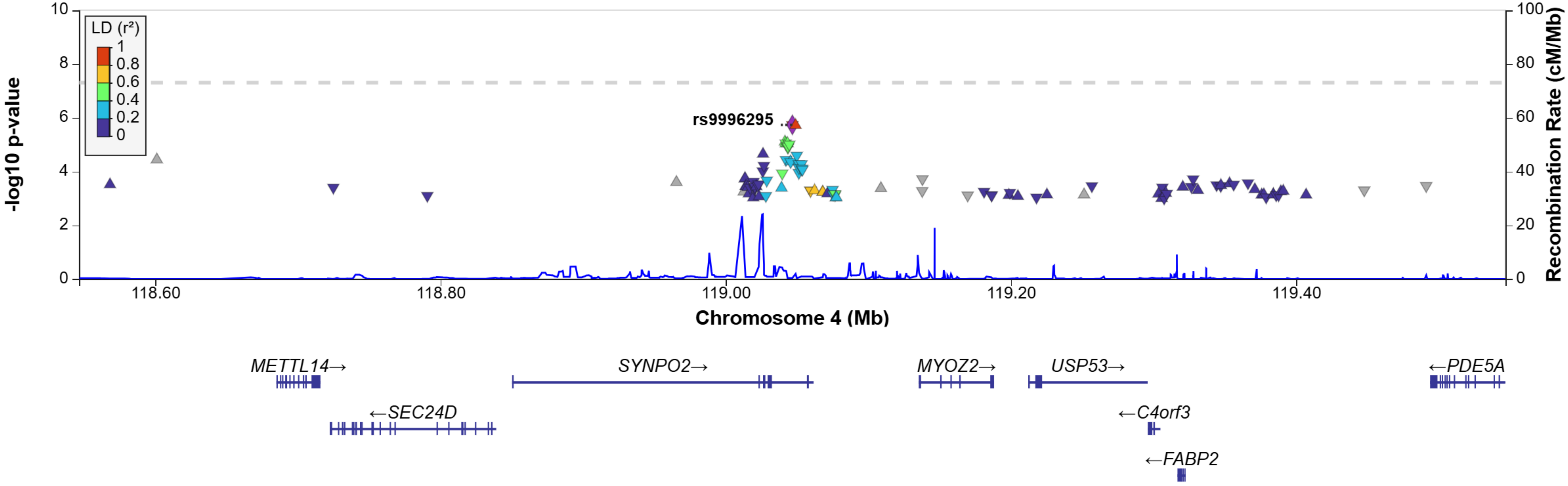

Supplementary Figure 11: Regional association plot for previously identified ovarian cancer region **chr4:164594195-166594195**

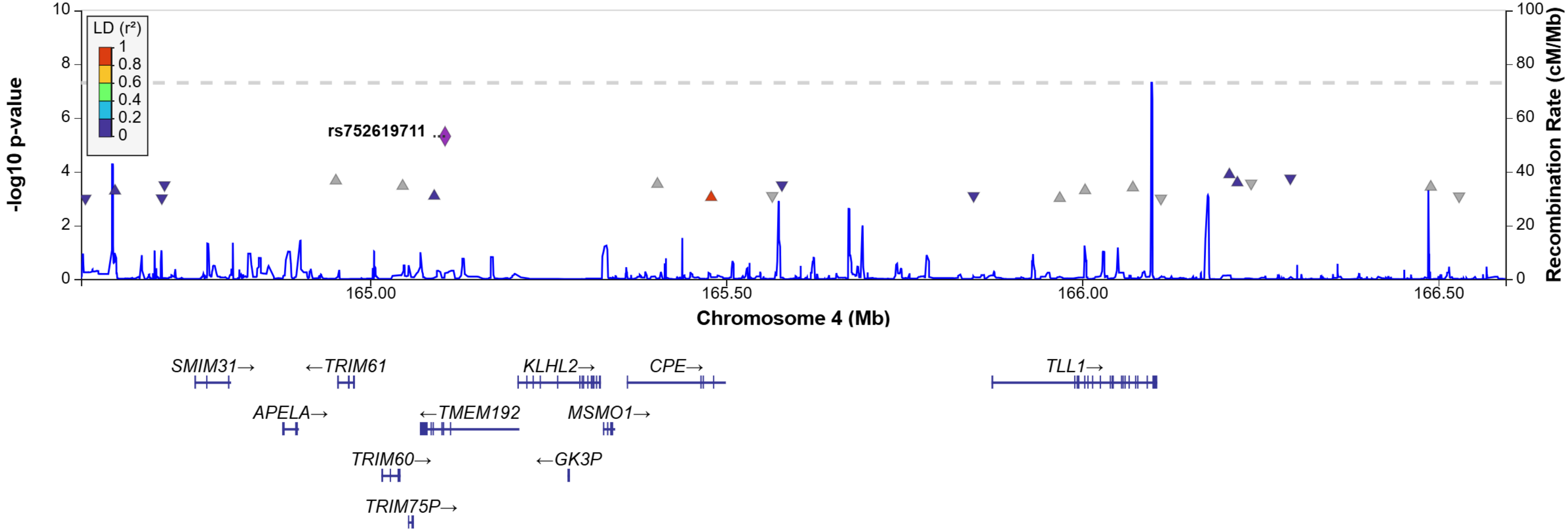

Supplementary Figure 12: Regional association plot for previously identified ovarian cancer region **chr4:69226524-70226524**

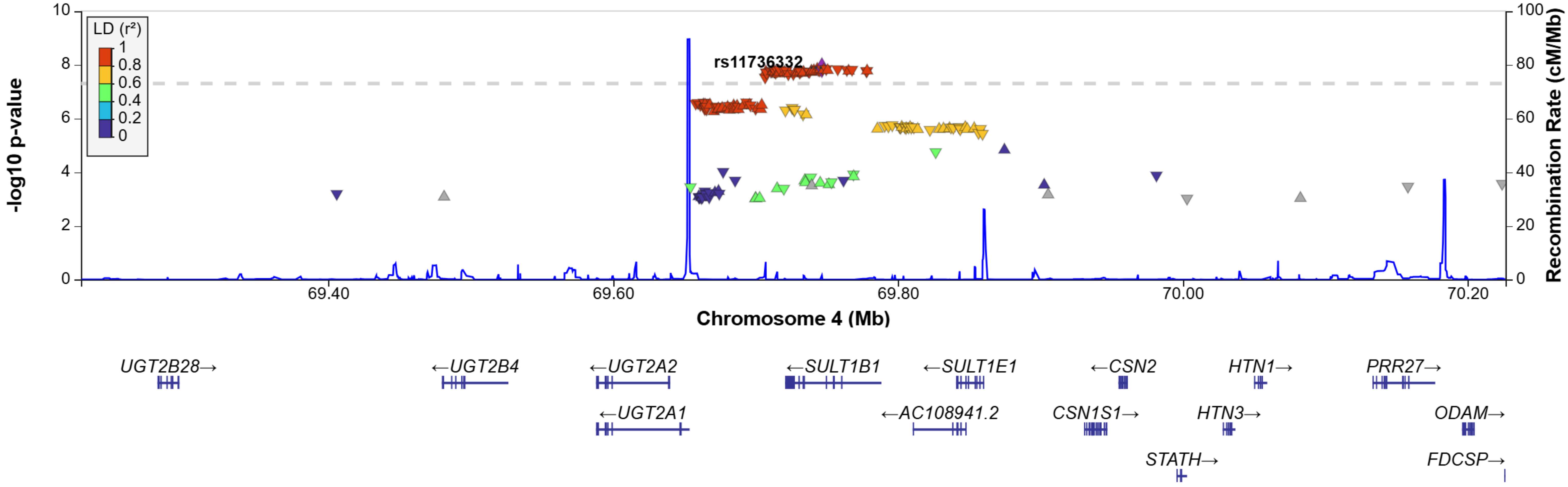

Supplementary Figure 13: Regional association plot for previously identified ovarian cancer region chr5:779675-1795234

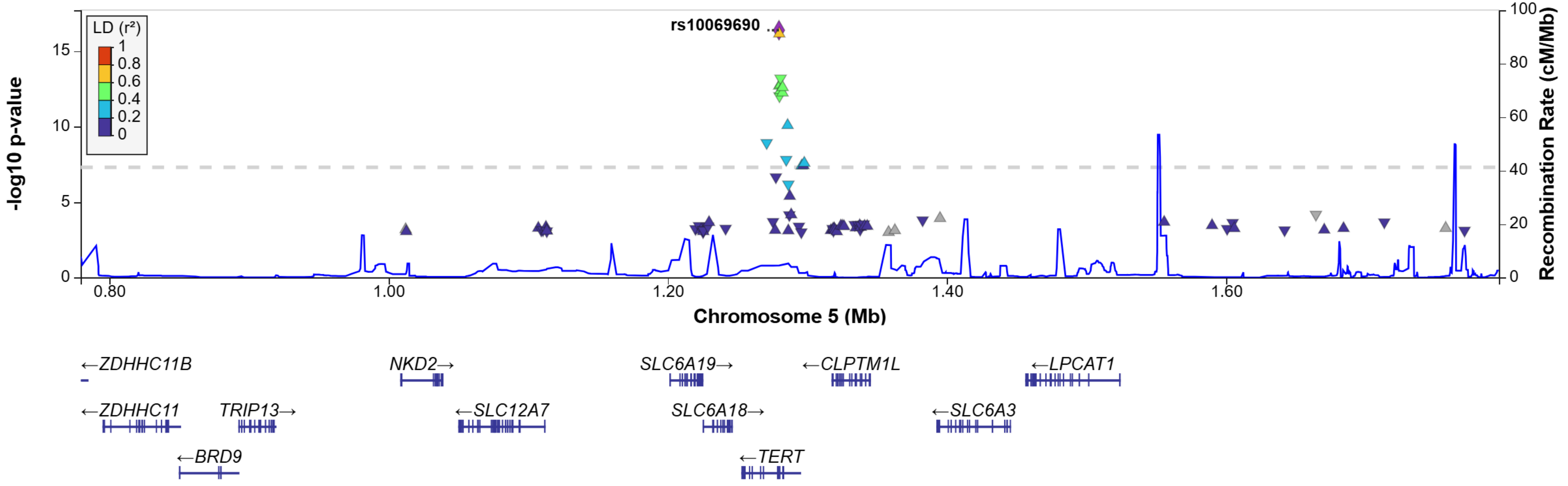

Supplementary Figure 14: Regional association plot for previously identified ovarian cancer region **chr5:54680727-55680728**

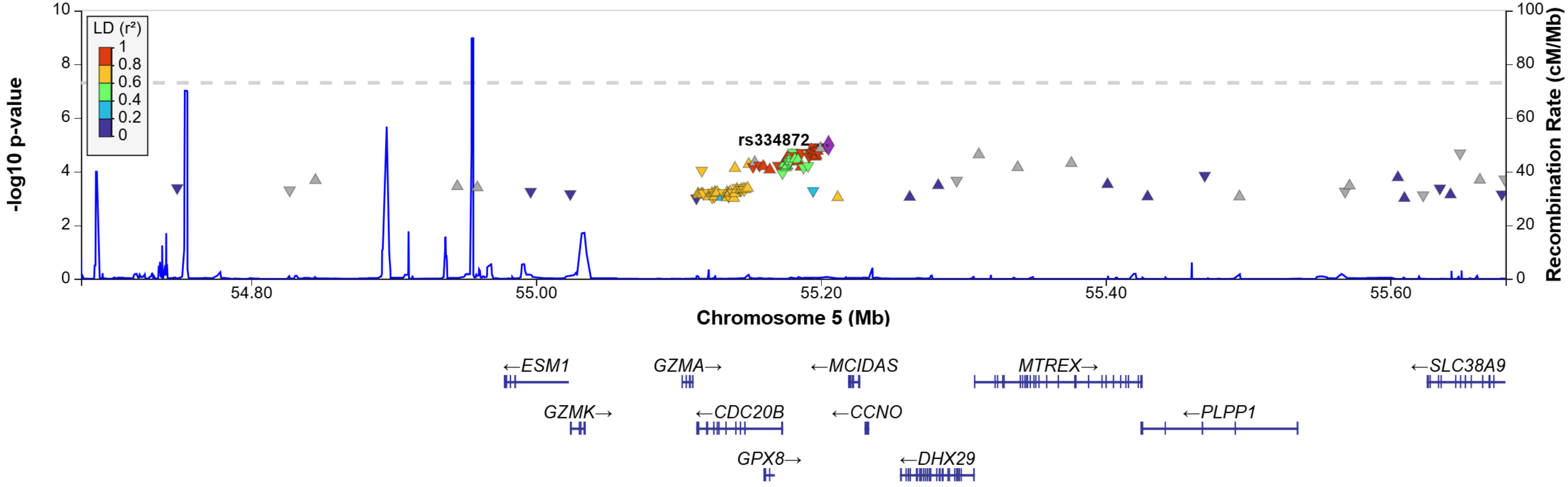

Supplementary Figure 15: Regional association plot for previously identified ovarian cancer region **chr5:66329868-67329868**

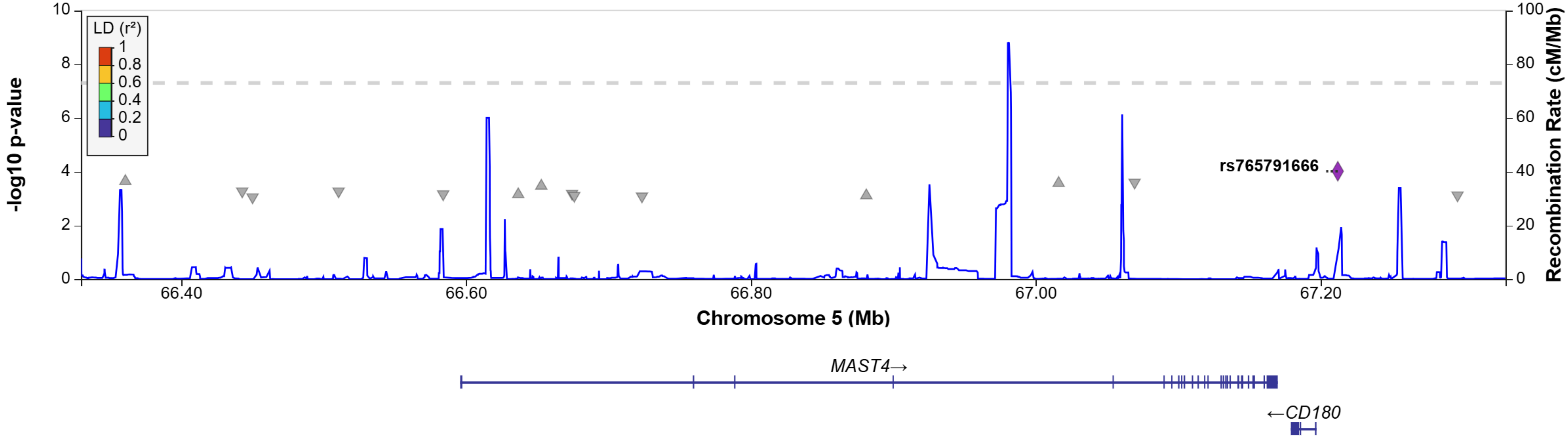

Supplementary Figure 16: Regional association plot for previously identified ovarian cancer region **chr6:27968282-28968283**

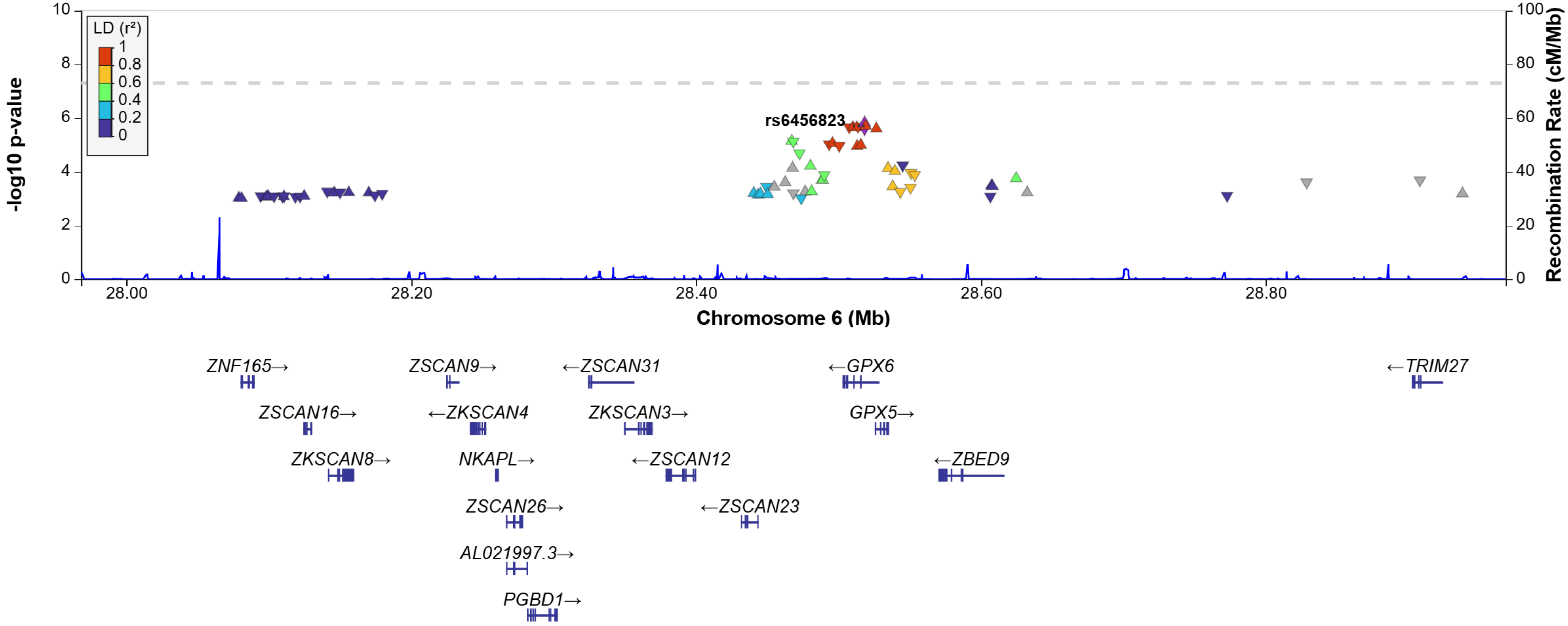

Supplementary Figure 17: Regional association plot for previously identified ovarian cancer region **chr8:75732128-76732128**

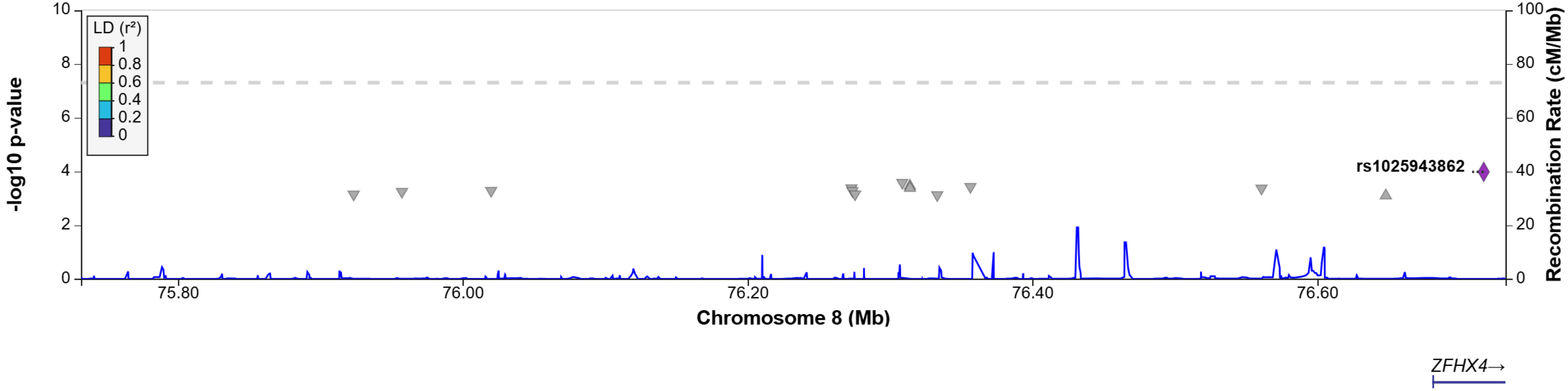

Supplementary Figure 18: Regional association plot for previously identified ovarian cancer region **chr8:81241409-82241409**

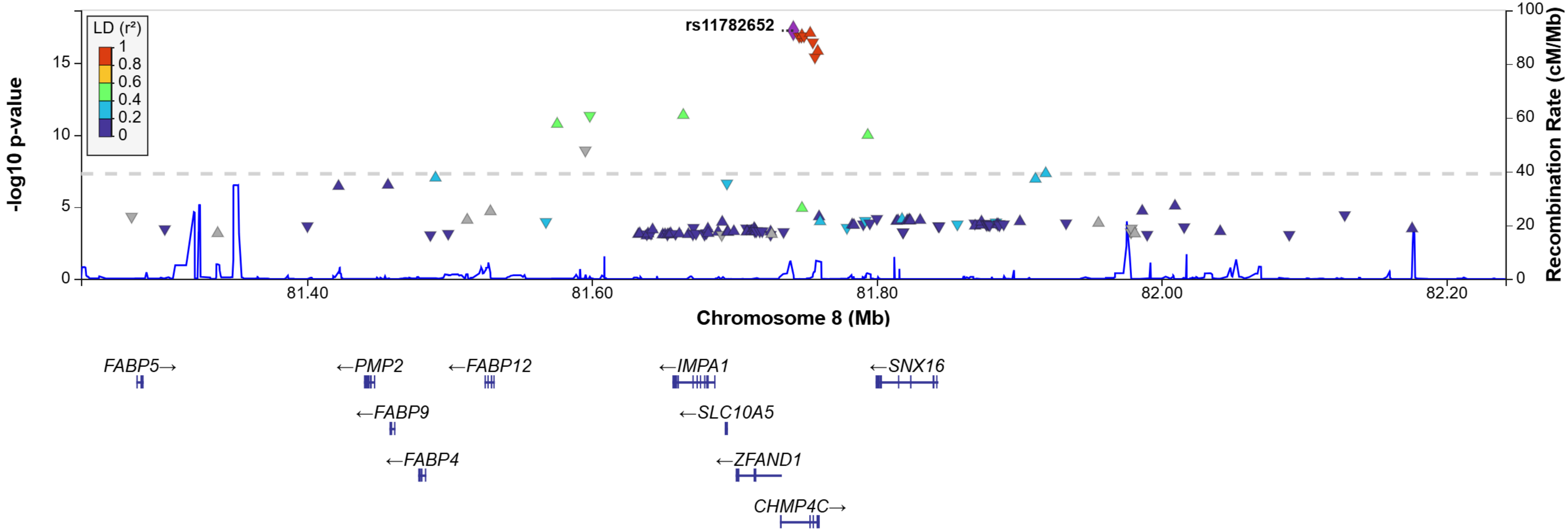

Supplementary Figure 19: Regional association plot for previously identified ovarian cancer region **chr8:126802672-128802672**

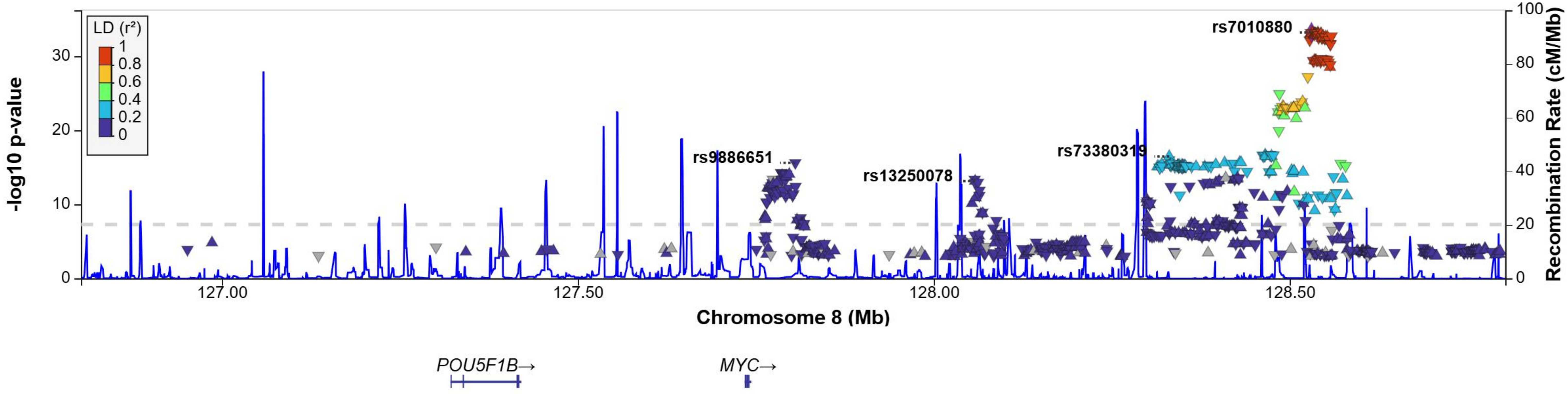

Supplementary Figure 20: Regional association plot for previously identified ovarian cancer region **chr9:101680944-102680944**

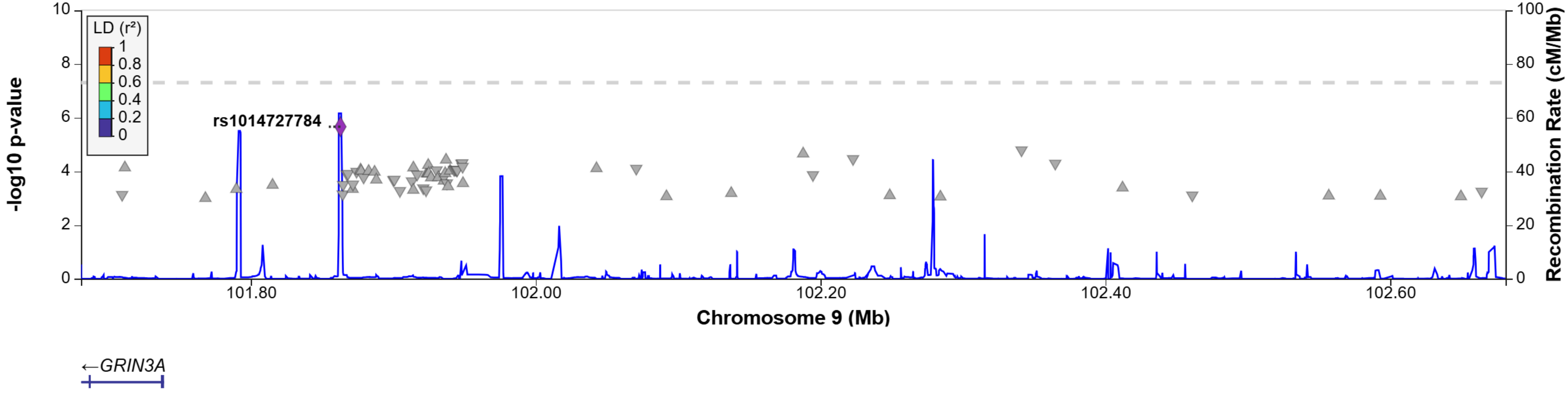

Supplementary Figure 21: Regional association plot for previously identified ovarian cancer region **chr9:103604421-104604422**

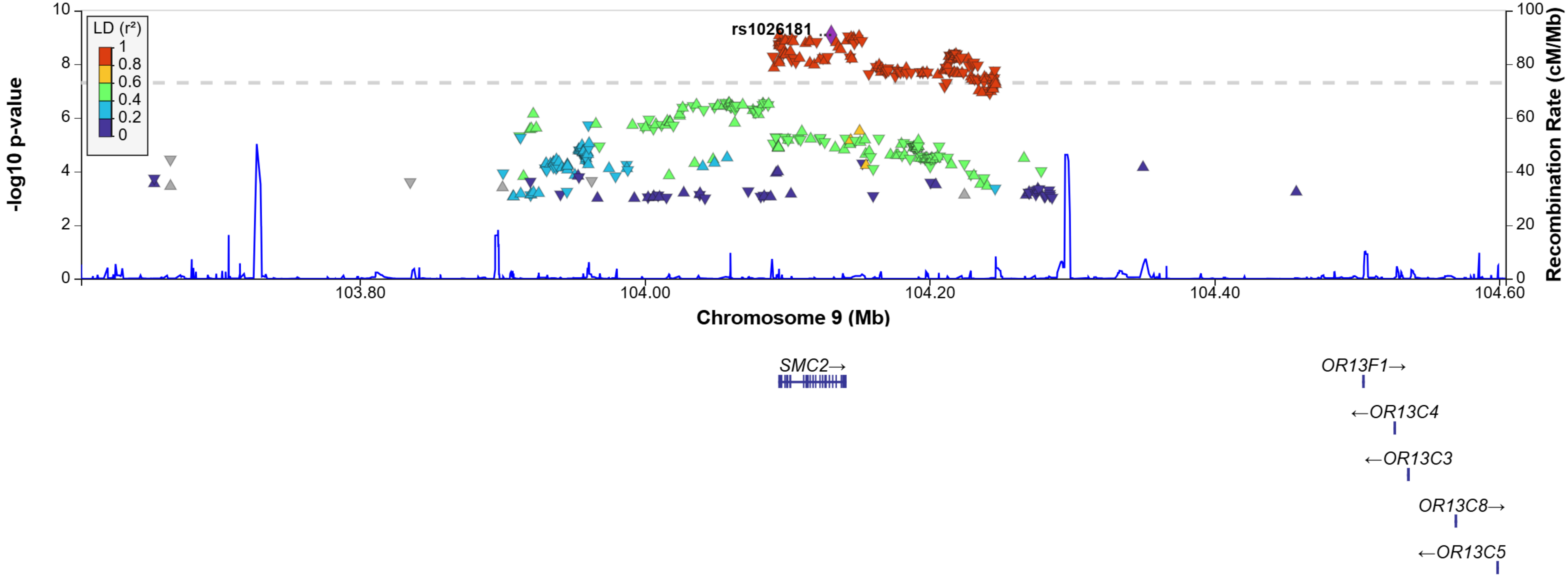

Supplementary Figure 22: Regional association plot for previously identified ovarian cancer region **chr9:132779613-133779427**

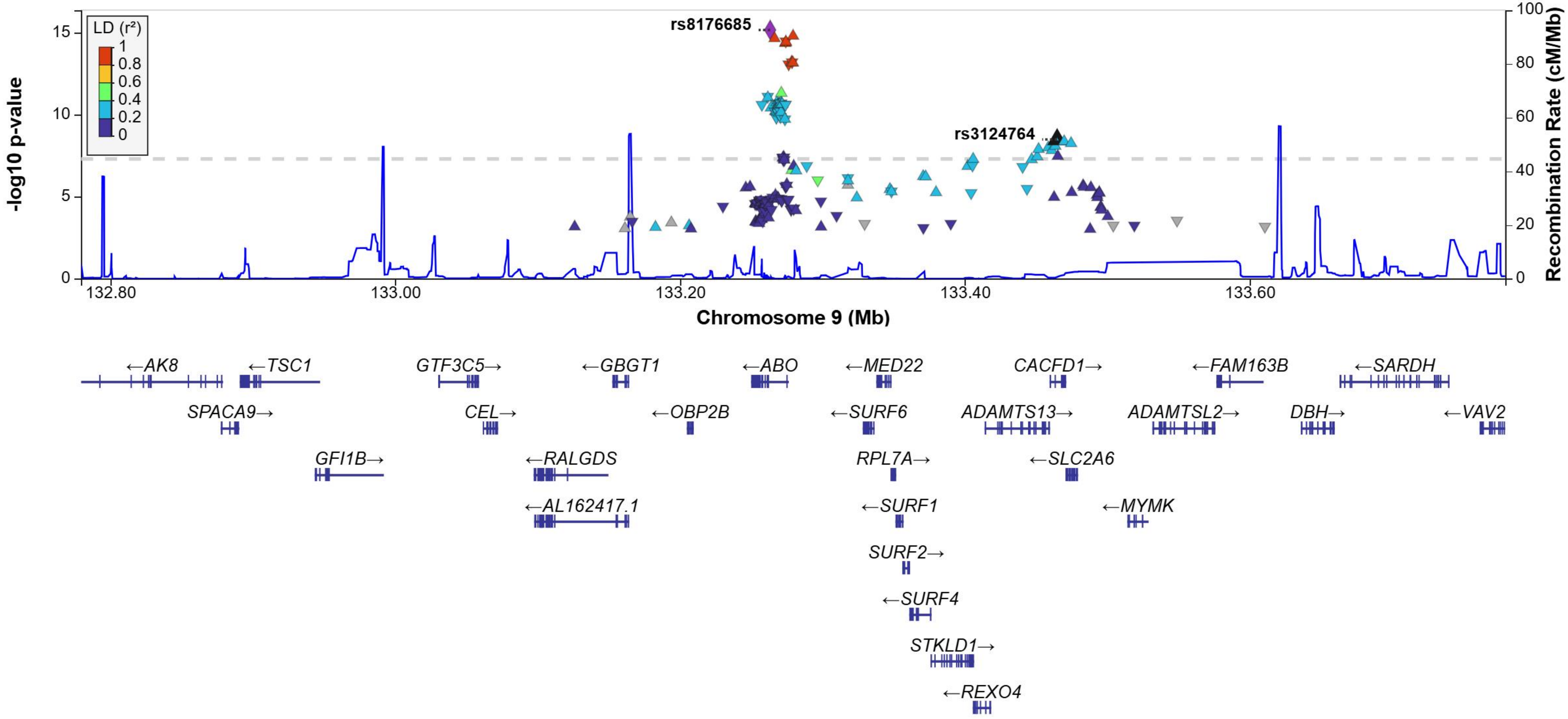

Supplementary Figure 23: Regional association plot for previously identified ovarian cancer region **chr9:16414718-17414718**

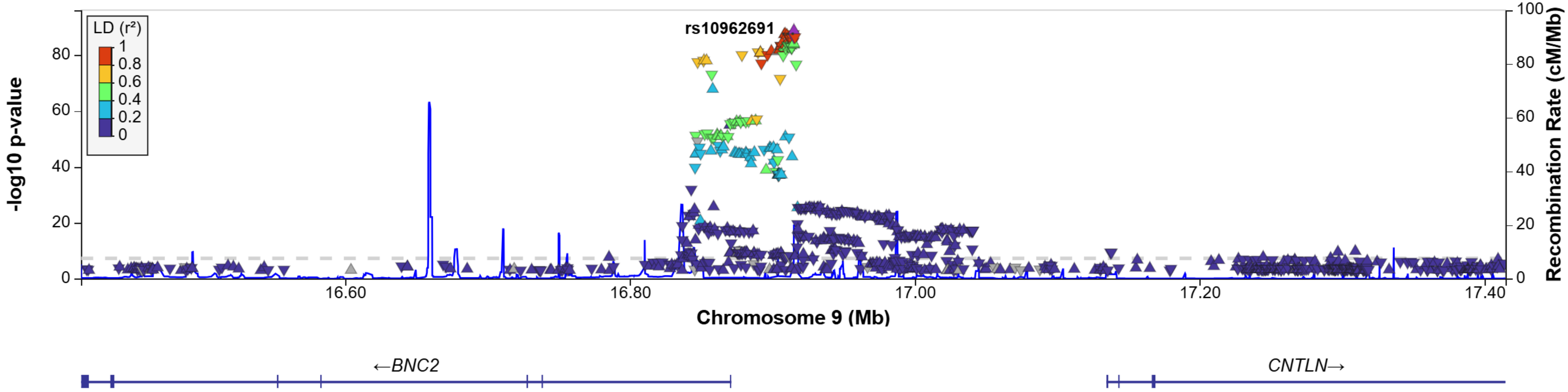

Supplementary Figure 24: Regional association plot for previously identified ovarian cancer region **chr9:18530885-19530885**

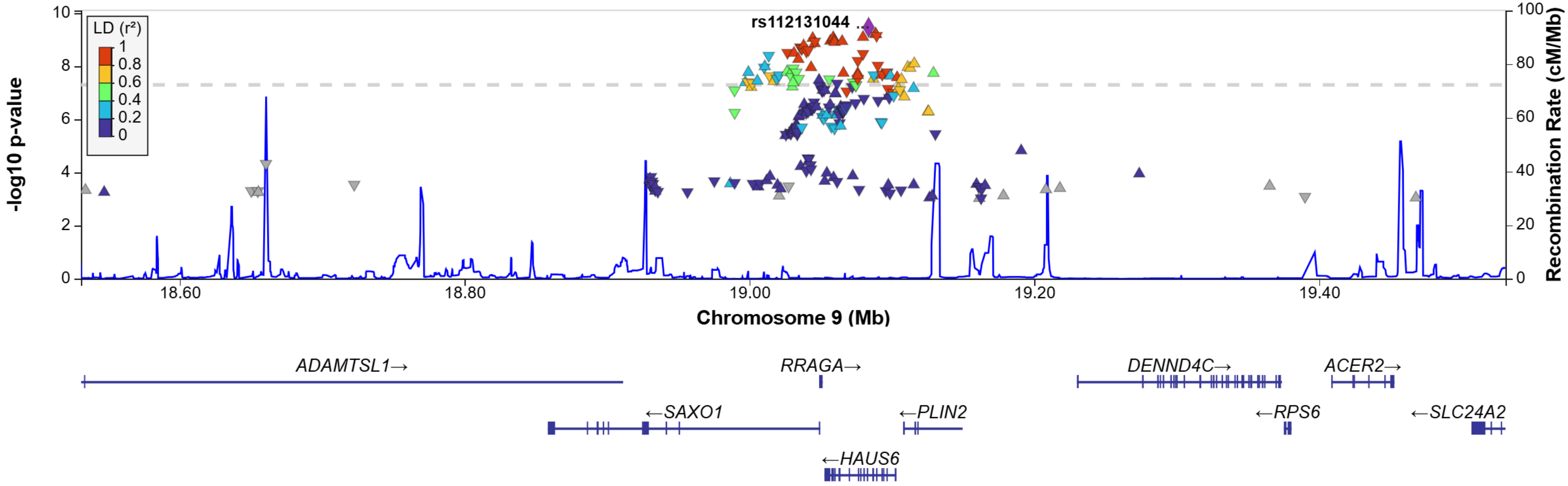

Supplementary Figure 25: Regional association plot for previously identified ovarian cancer region **chr9:98498959-99498959**

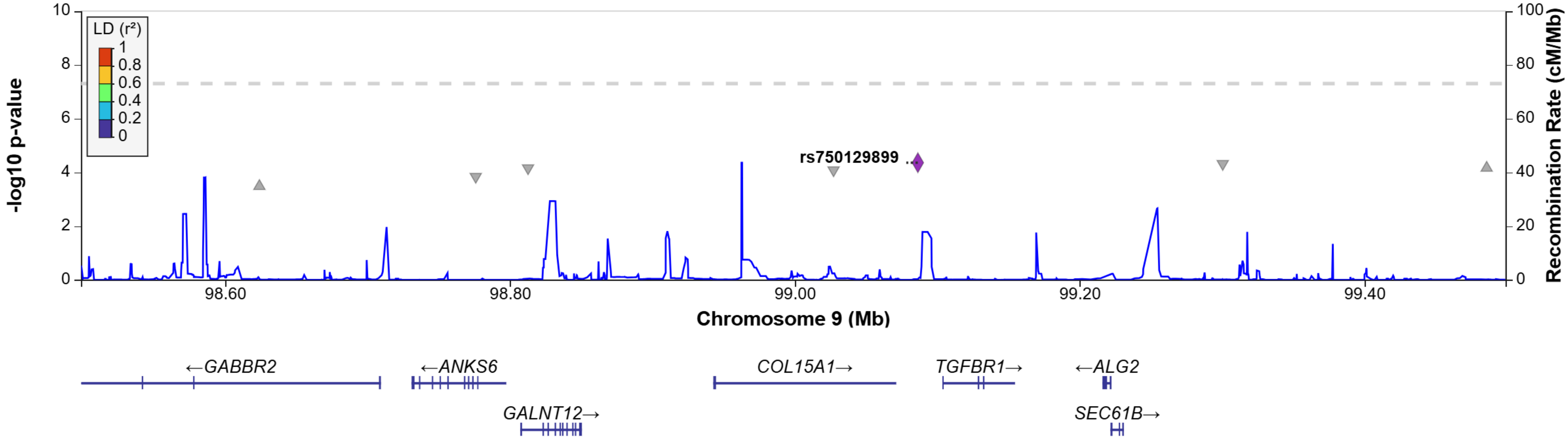

Supplementary Figure 26: Regional association plot for previously identified ovarian cancer region **chr10:103434544-104434543**

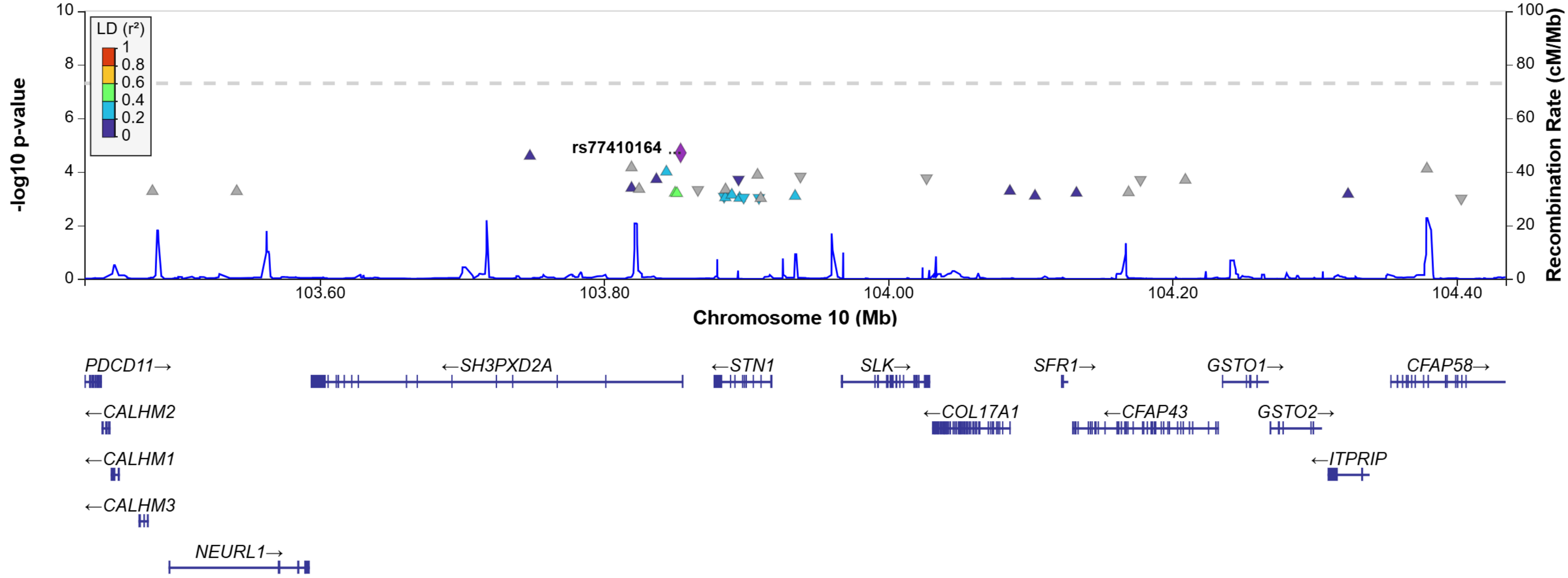

Supplementary Figure 27: Regional association plot for previously identified ovarian cancer region **chr10:21032345-22032345**

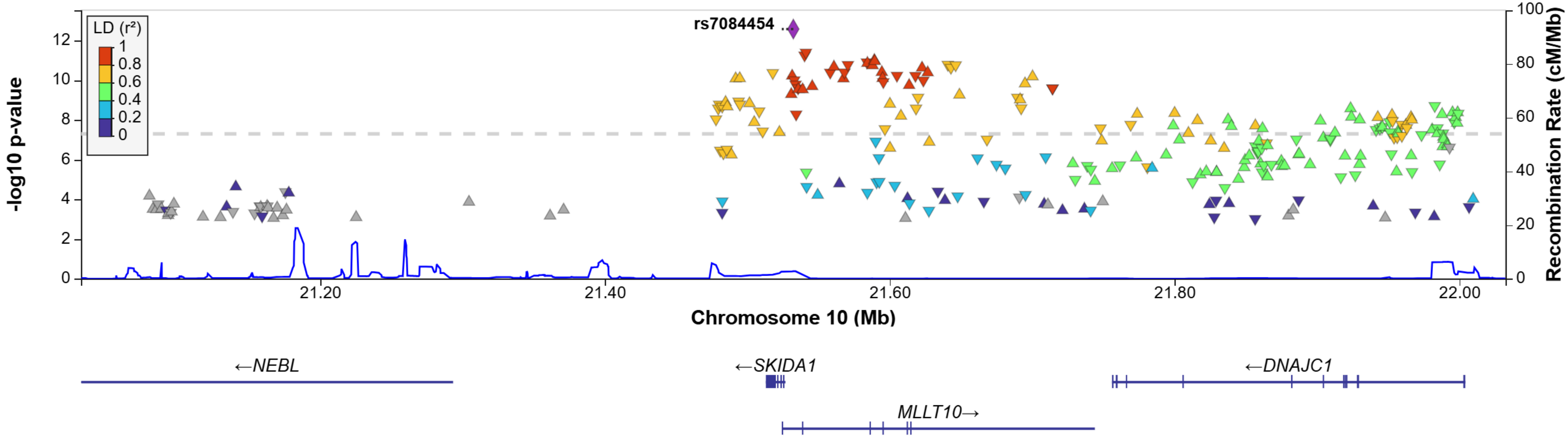

Supplementary Figure 28: Regional association plot for previously identified ovarian cancer region **chr10:36380367-37380367**

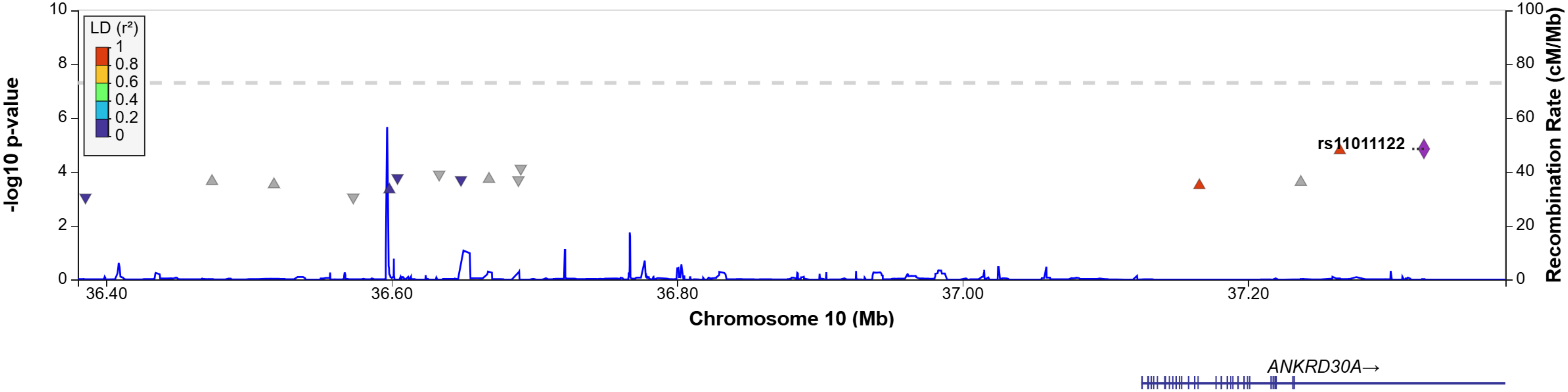

Supplementary Figure 29: Regional association plot for previously identified ovarian cancer region **chr11:61626500-62626500**

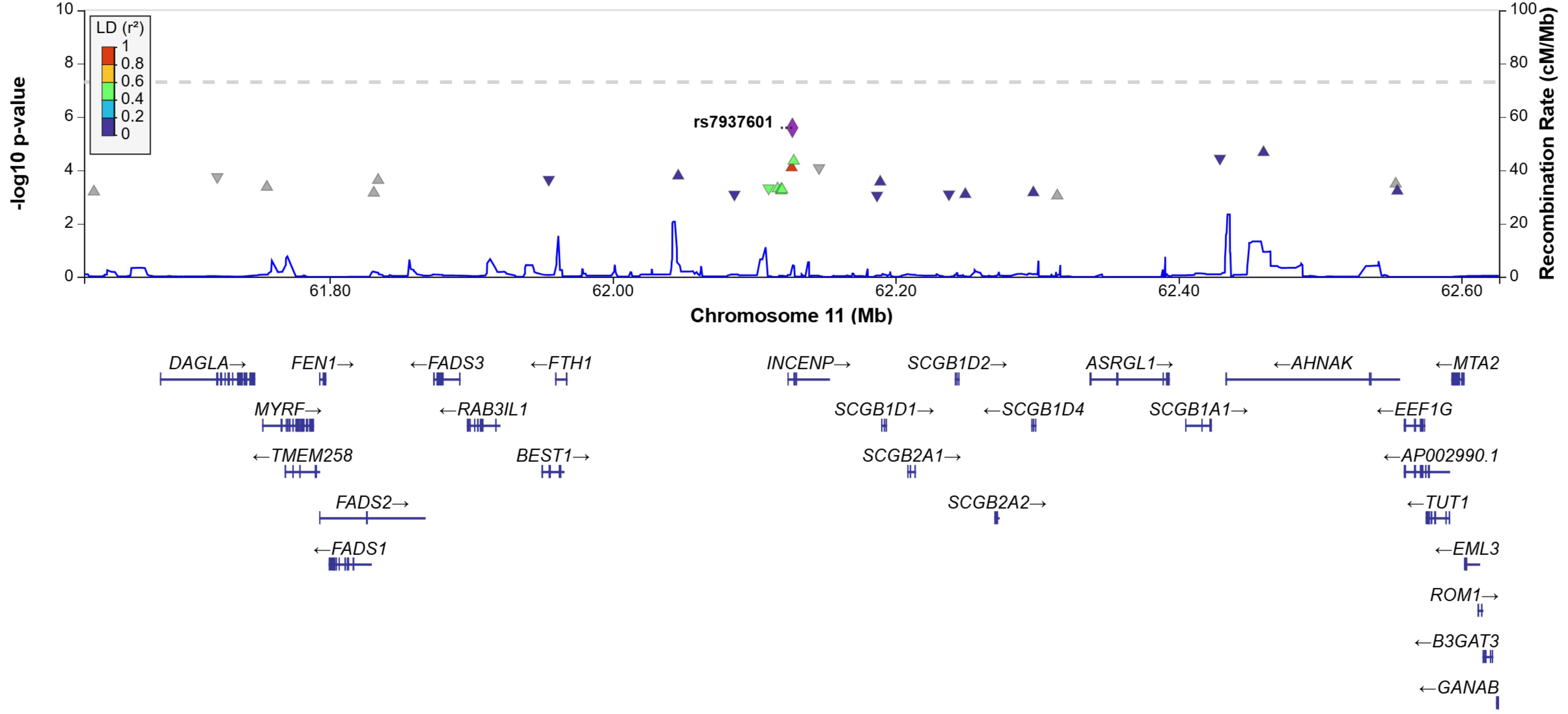

Supplementary Figure 30: Regional association plot for previously identified ovarian cancer region **chr12:120465921-121465921**

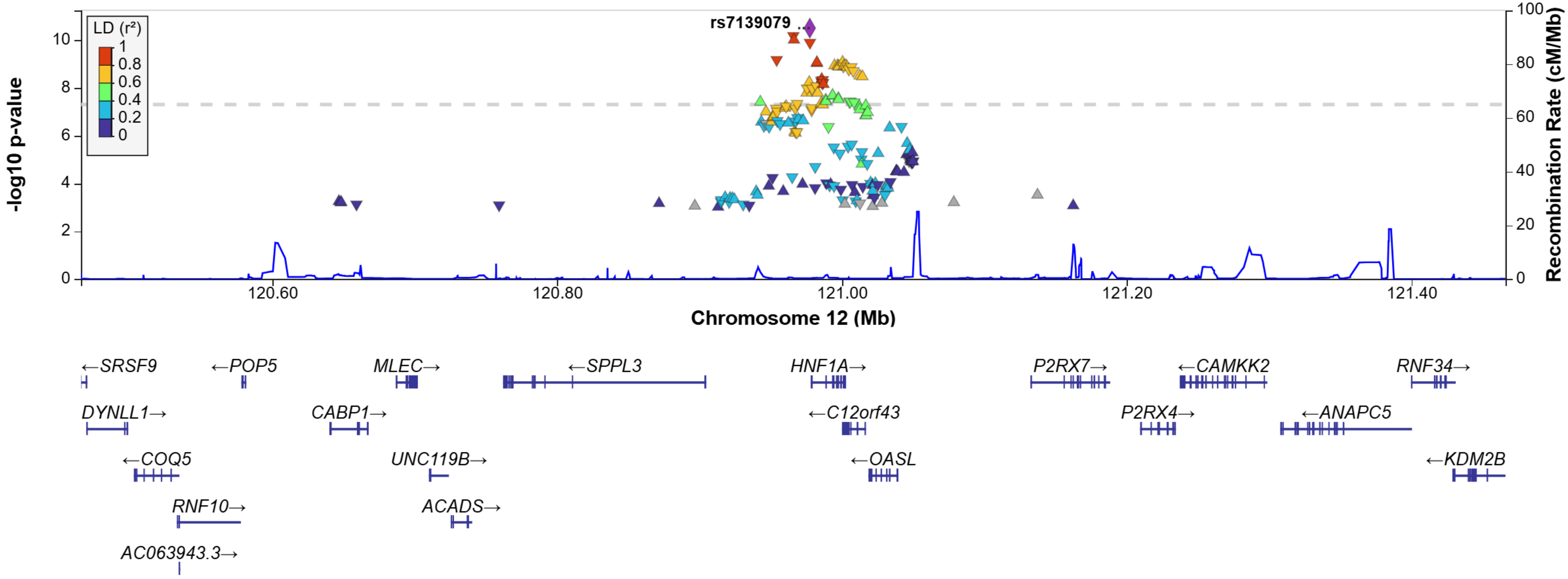

Supplementary Figure 31: Regional association plot for previously identified ovarian cancer region **chr15:90492097-91492099**

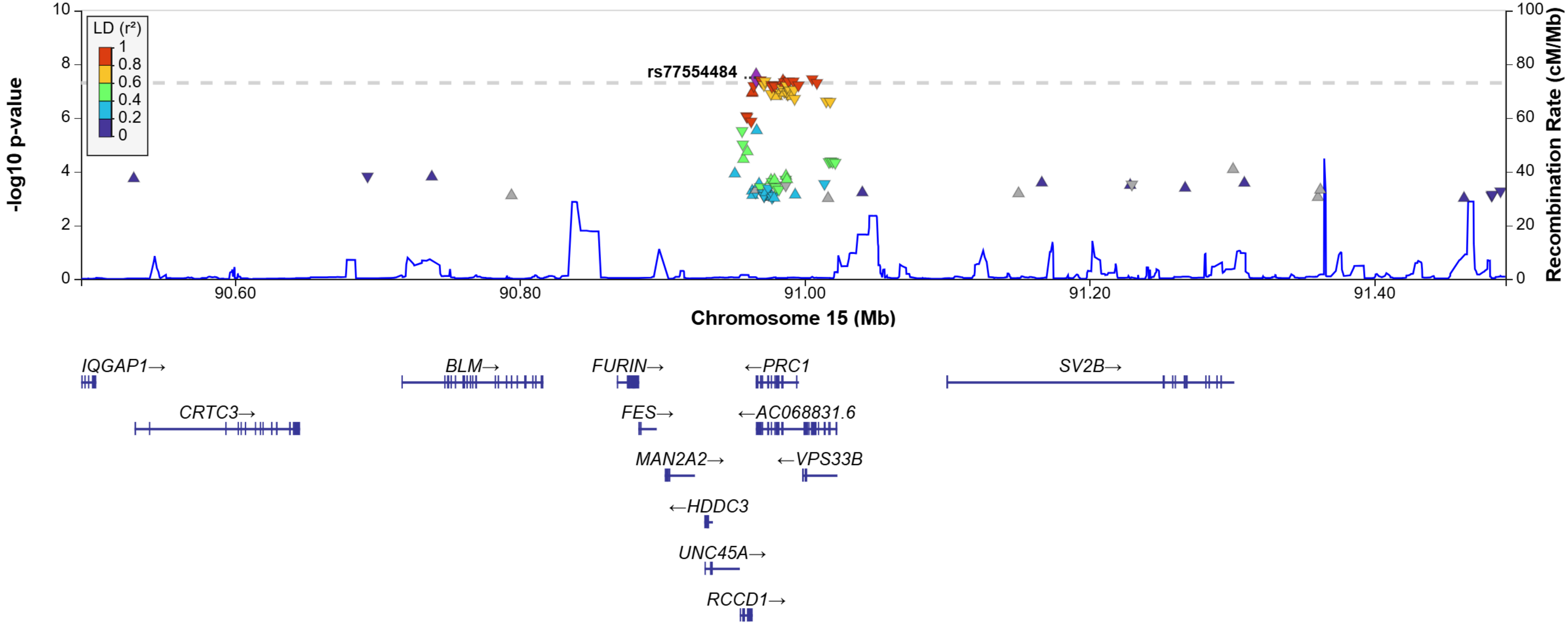

Supplementary Figure 32: Regional association plot for previously identified ovarian cancer region **chr17:30339284-31339284**

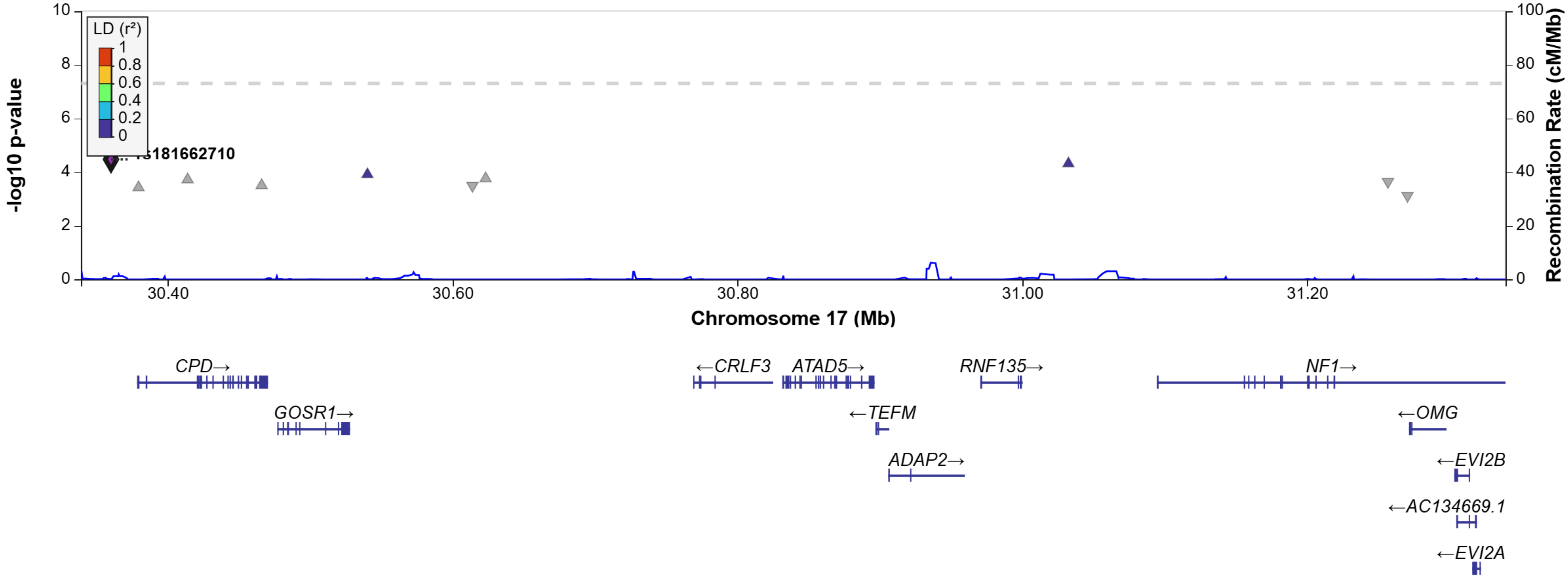

Supplementary Figure 33: Regional association plot for previously identified ovarian cancer region **chr17:37243851-38240776**

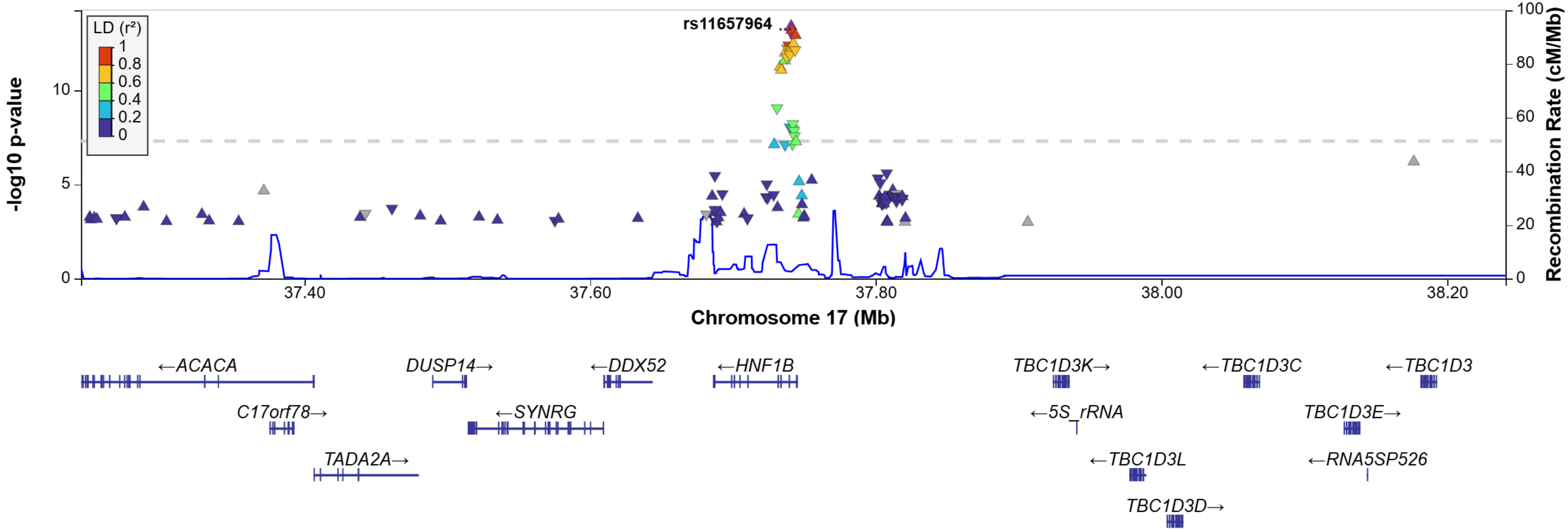

Supplementary Figure 34: Regional association plot for previously identified ovarian cancer region **chr17:45109891-47109891**

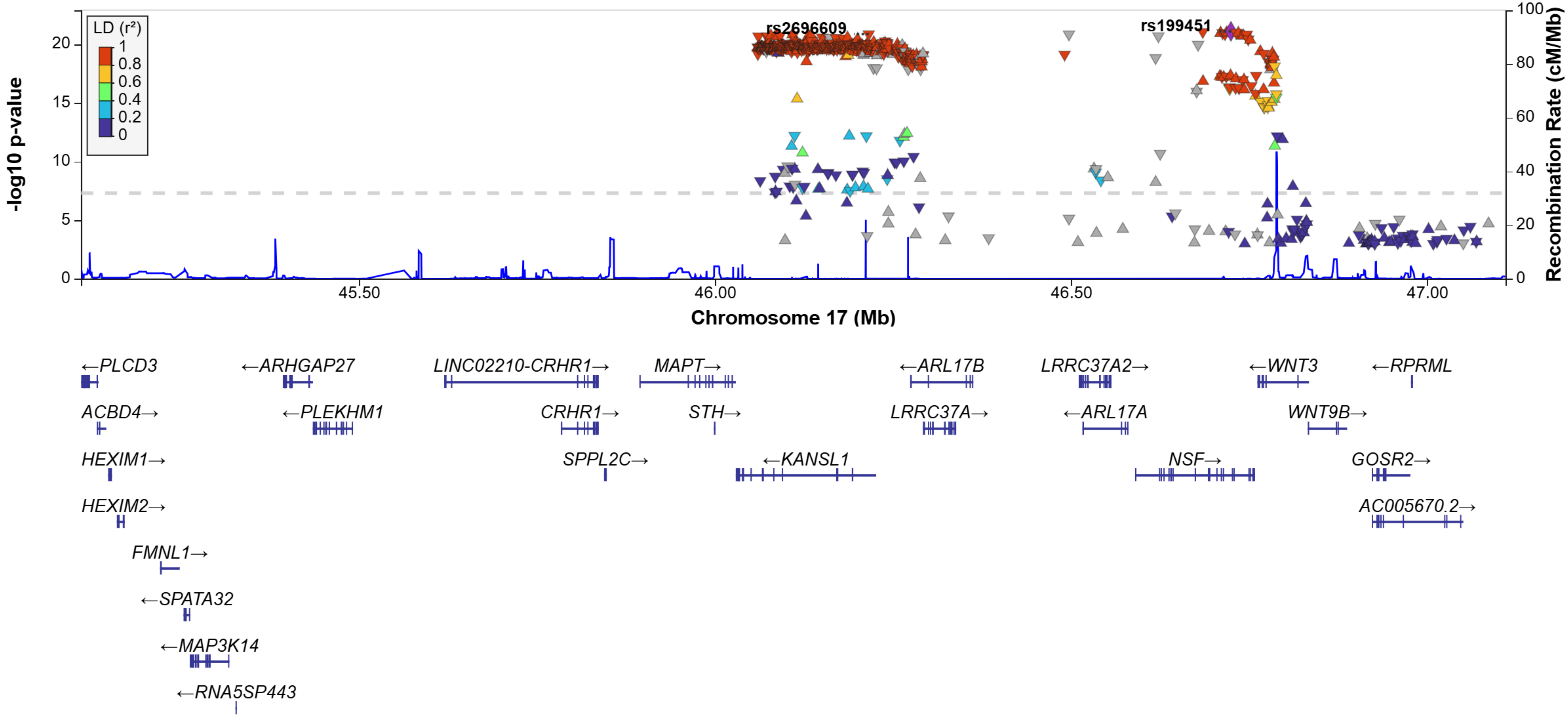

Supplementary Figure 35: Regional association plot for previously identified ovarian cancer region **chr17:47395070-49395070**

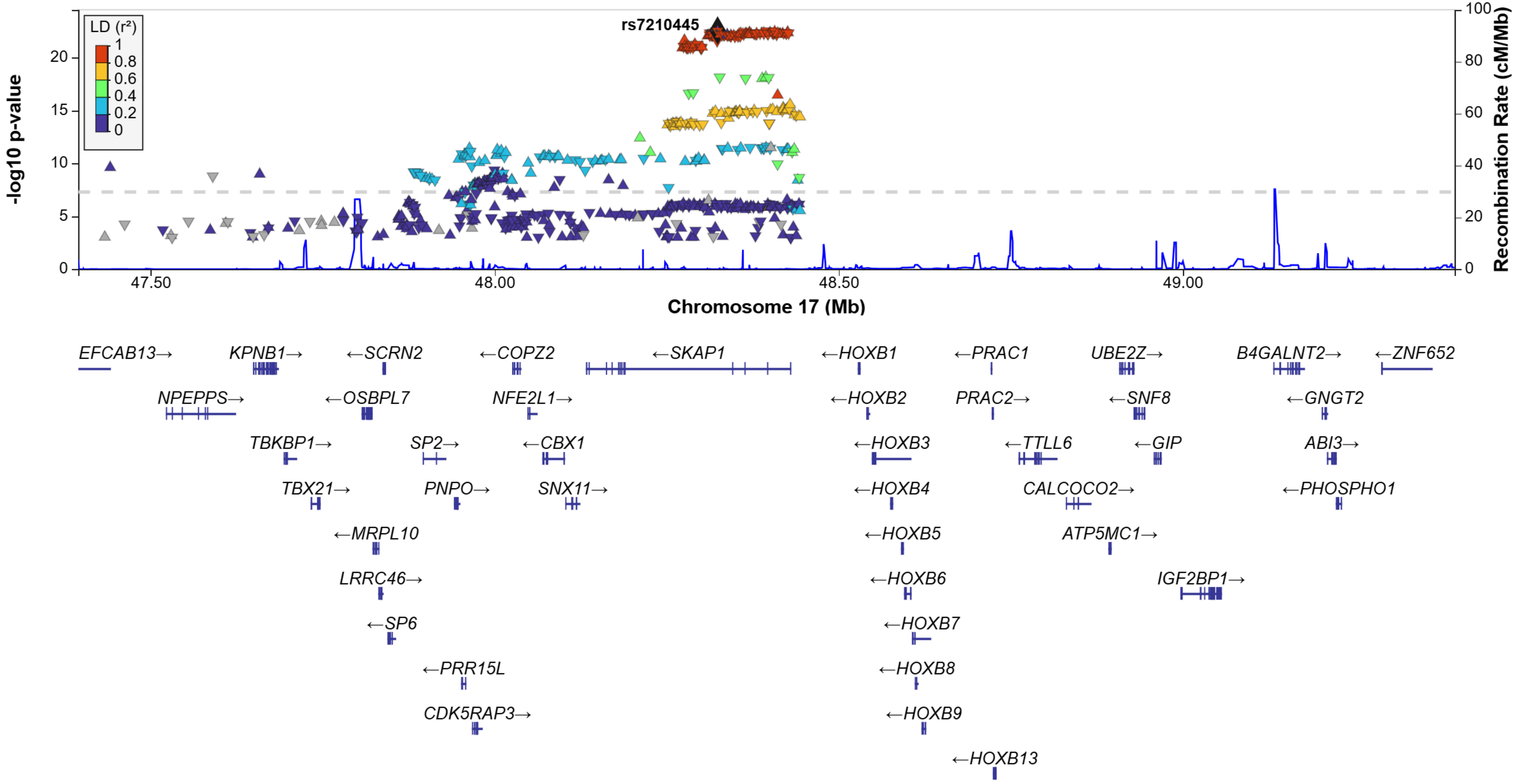

Supplementary Figure 36: Regional association plot for previously identified ovarian cancer region **chr18:23345888-24345888**

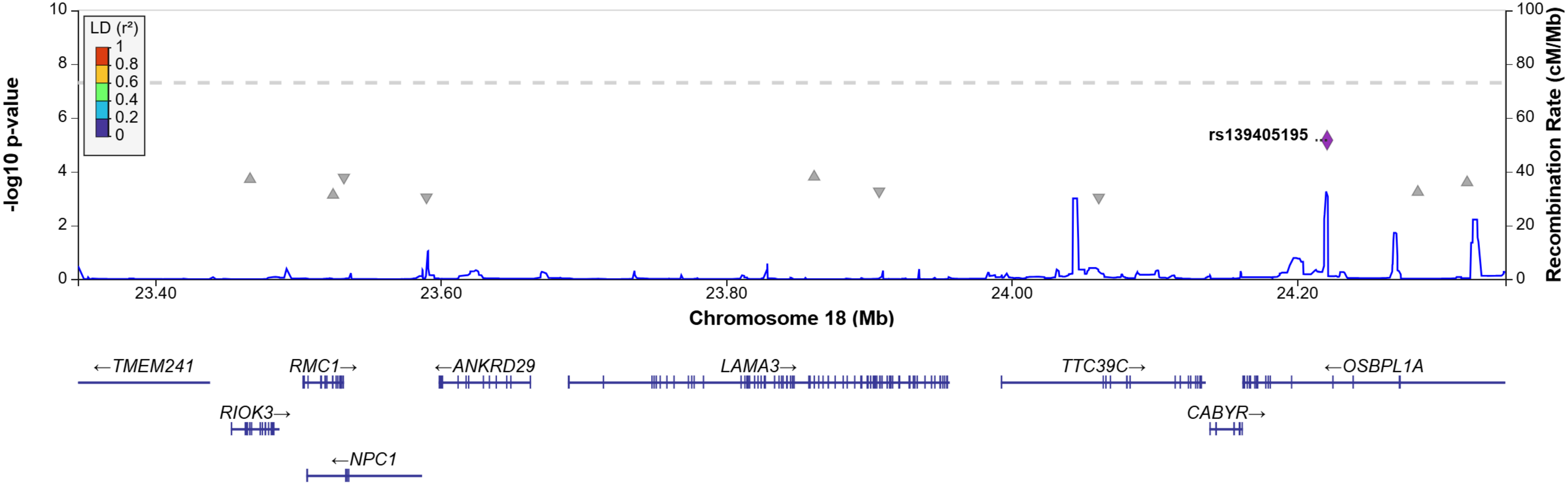

Supplementary Figure 37: Regional association plot for previously identified ovarian cancer region **chr19:16779480-17779482**

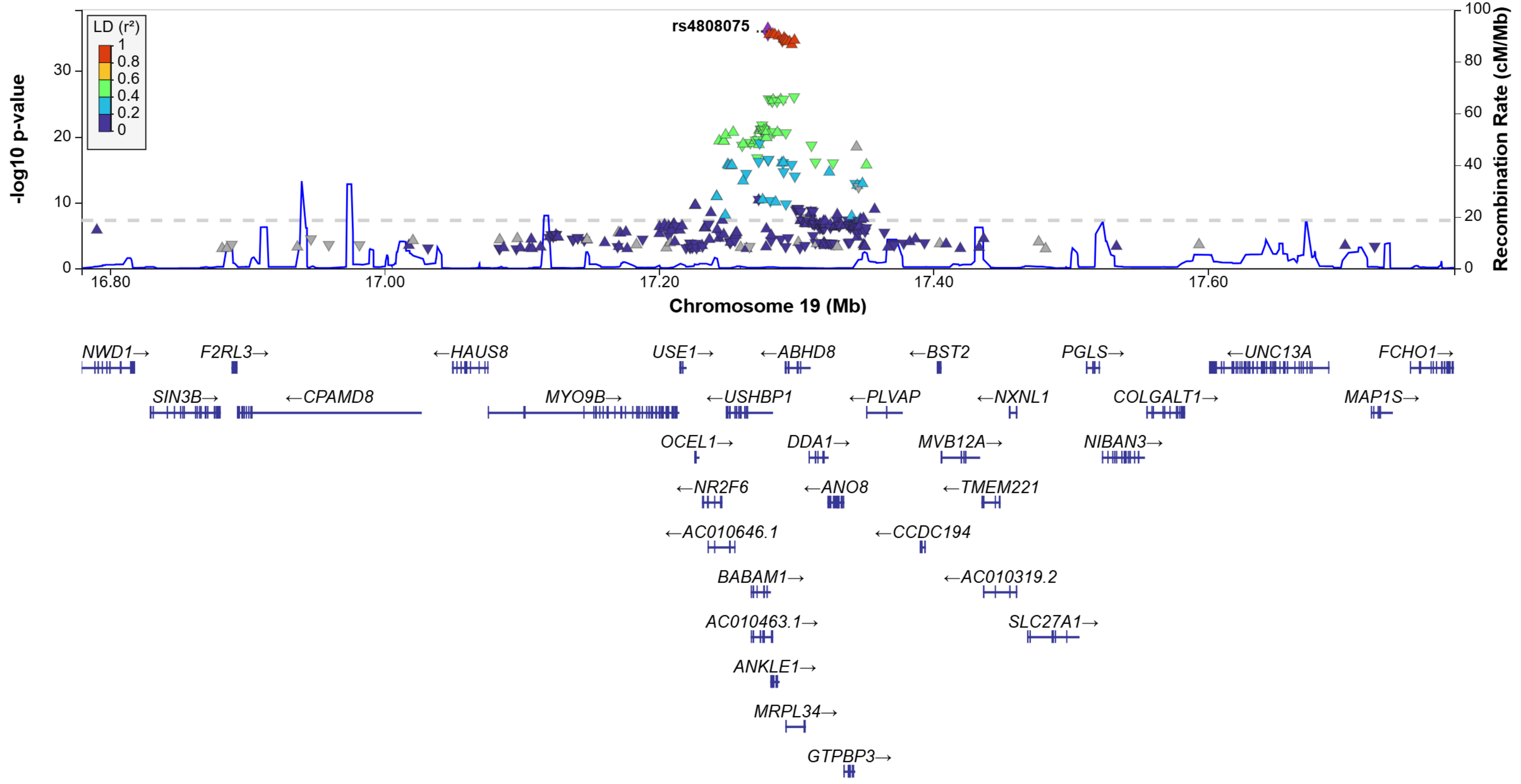

Supplementary Figure 38: Regional association plot for previously identified ovarian cancer region **chr19:18917997-19917997**

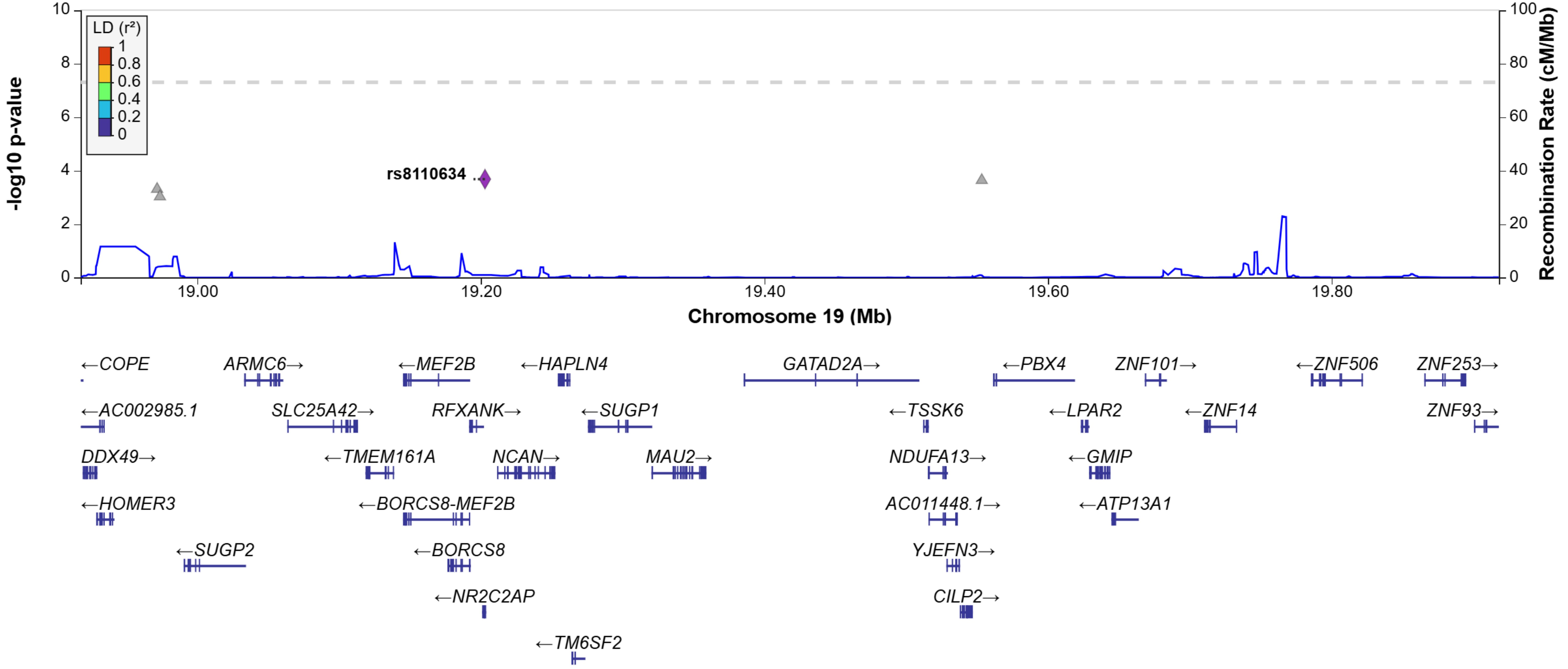

Supplementary Figure 39: Regional association plot for previously identified ovarian cancer region **chr19:38748147-39748147**

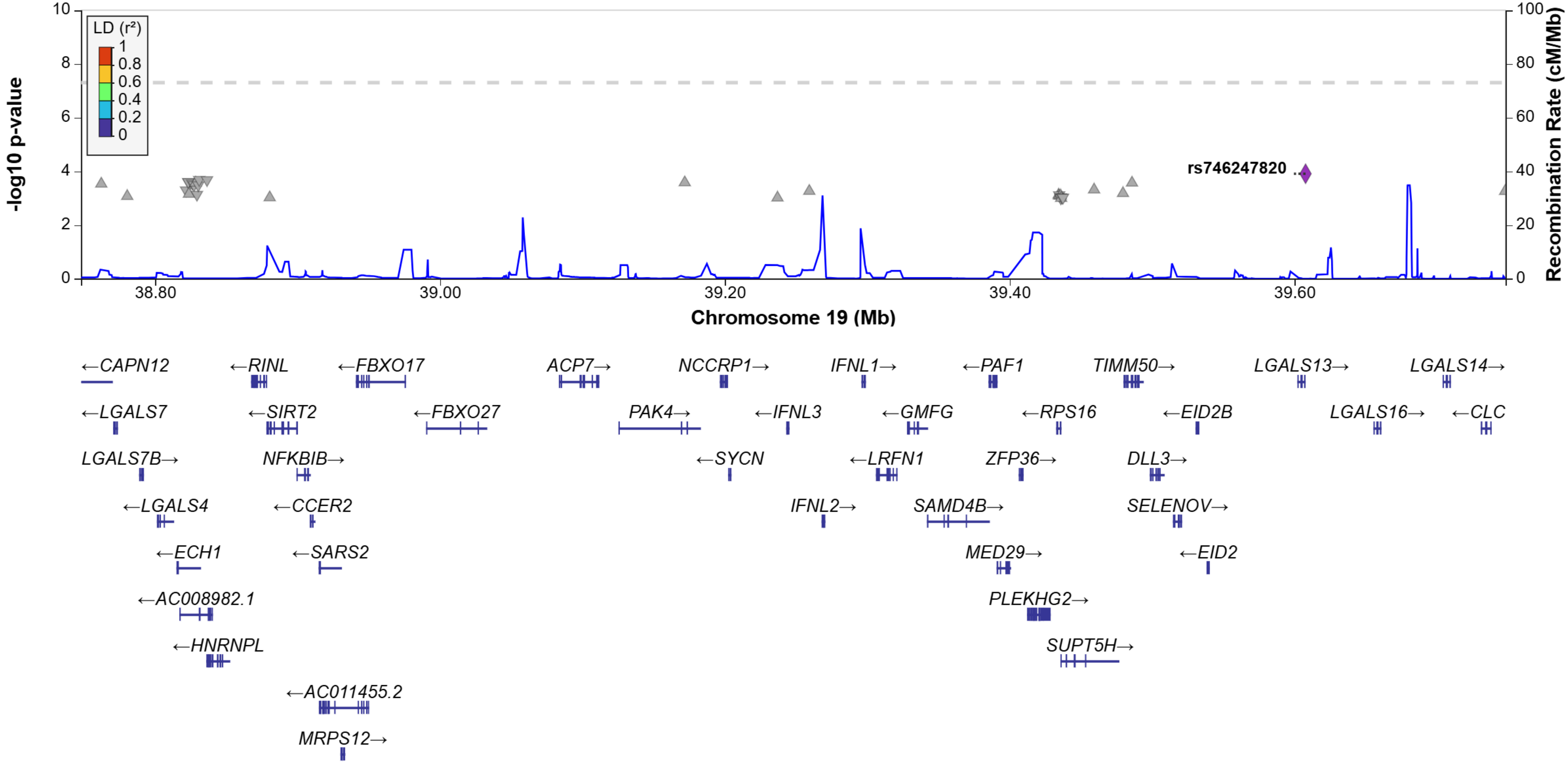

Supplementary Figure 40: Regional association plot for previously identified ovarian cancer region **chr21:34208097-35208100**

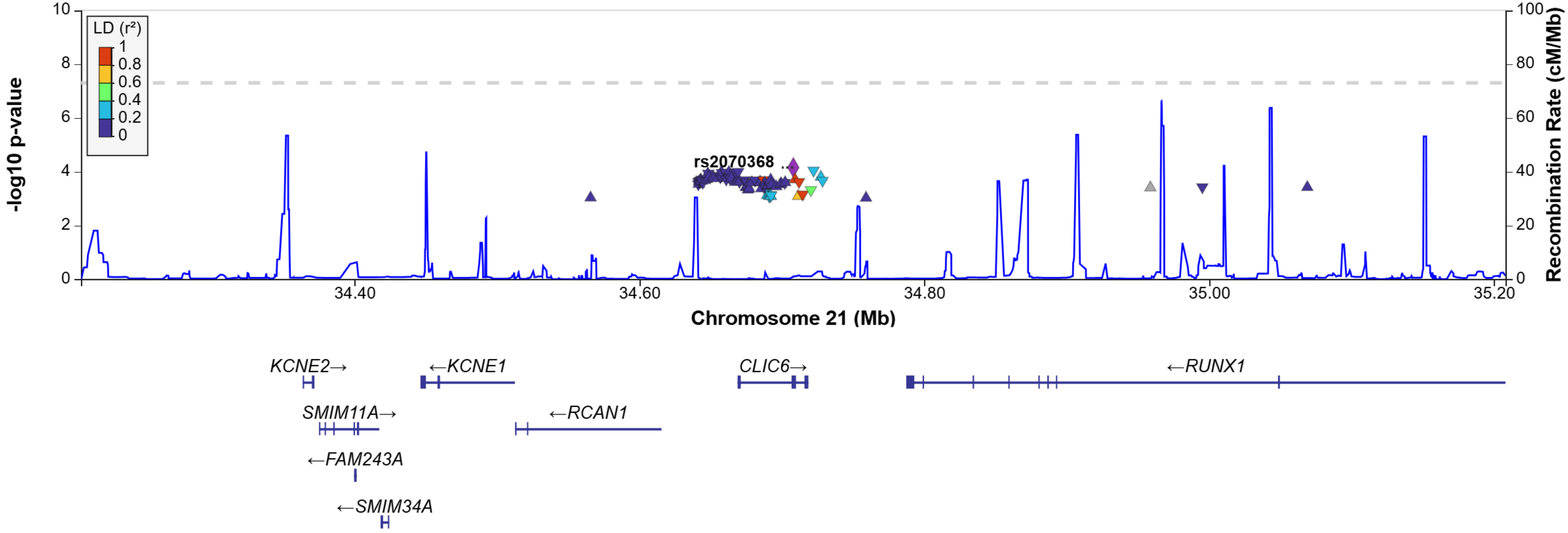

Supplementary Figure 41: Regional association plot for previously identified ovarian cancer region **chr22:28038325-29038325**

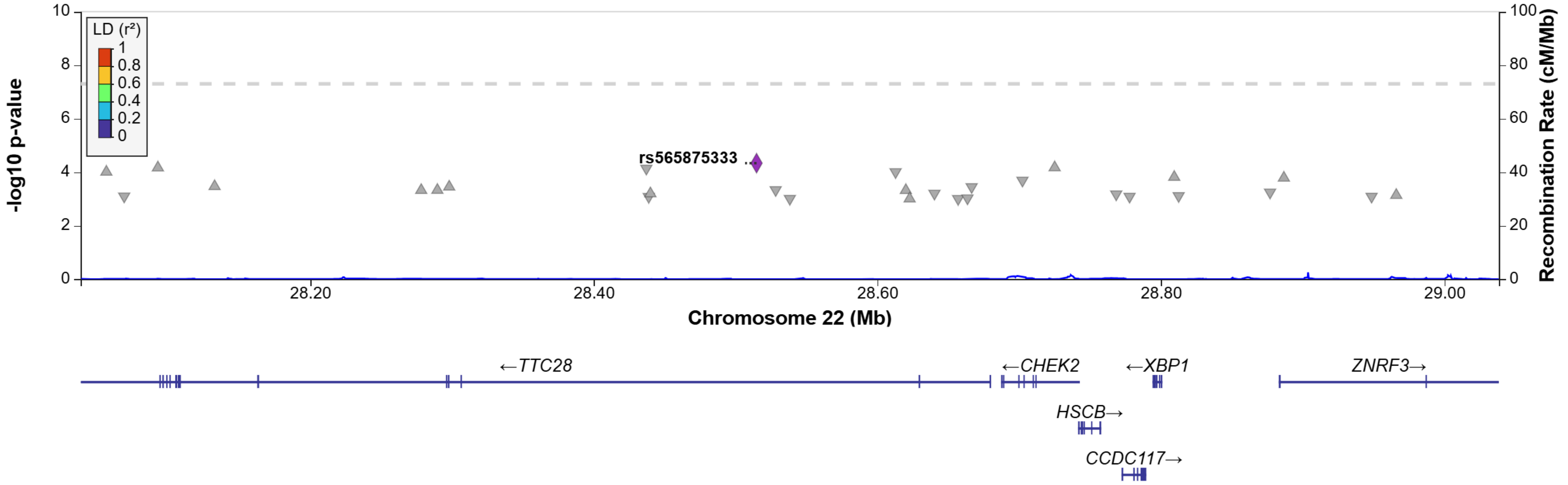

Supplementary Figure 42: Regional association plot with credible causal variants for novel region **5q11.2 rs528577783 (near *FST*)**

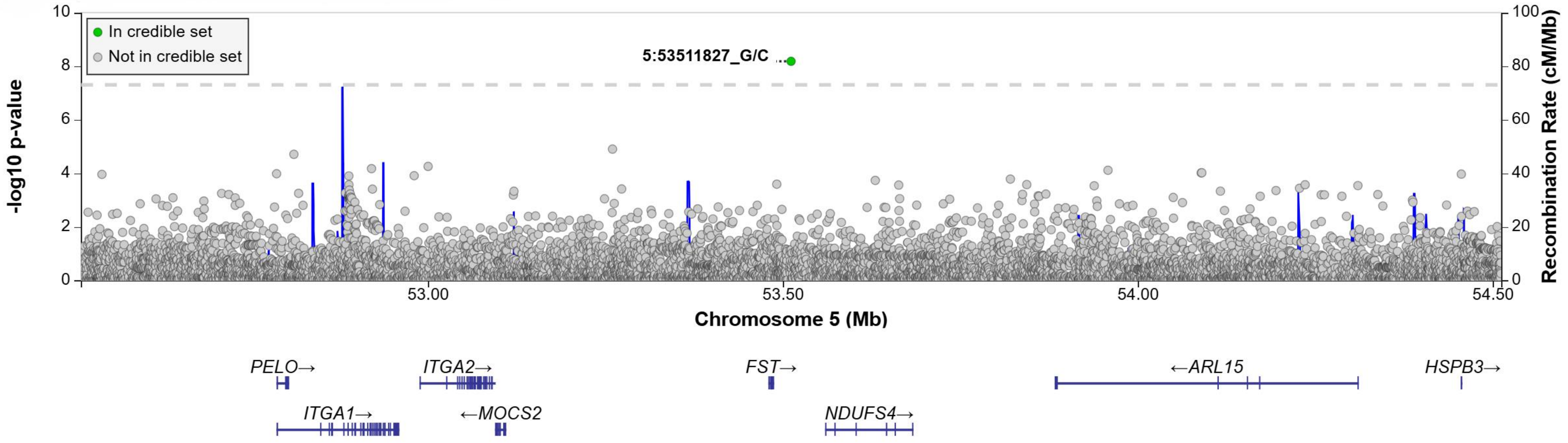

Supplementary Figure 43: Regional association plot with credible causal variants for novel region **6p12 rs1013698558 (near *GCLC*)**

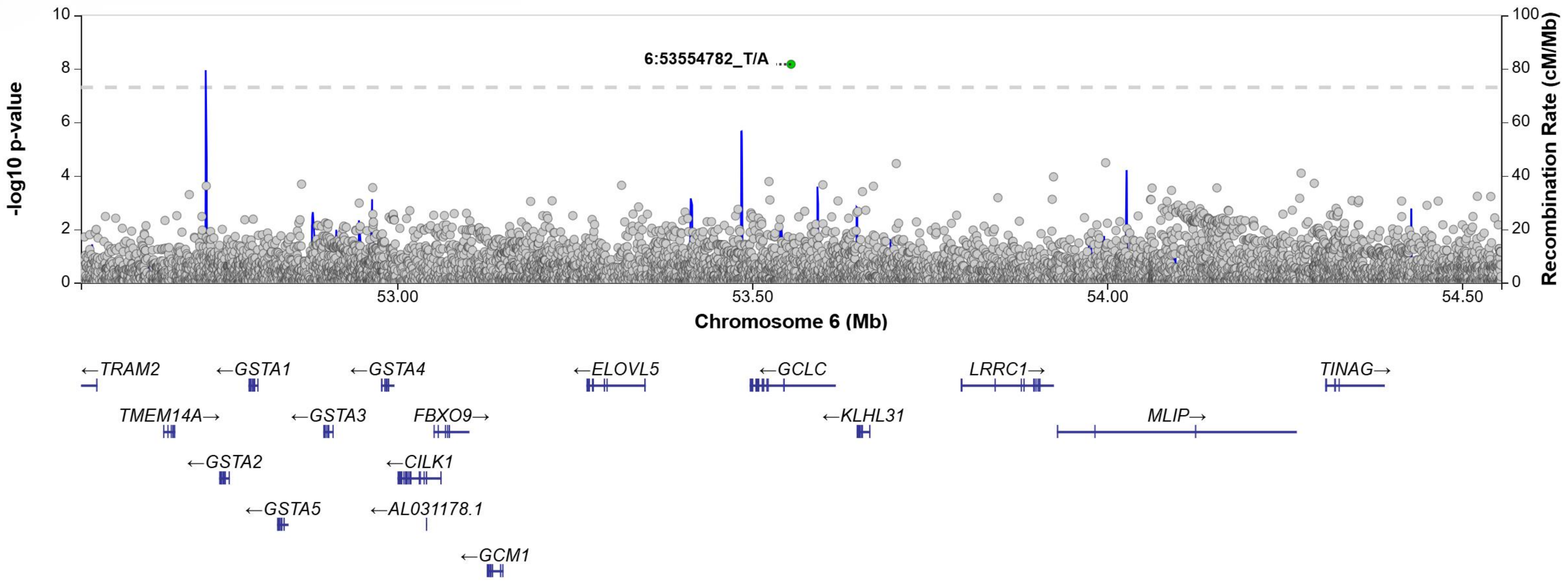

Supplementary Figure 44: Regional association plot with credible causal variants for novel region **8p21 rs540569242 (near *CDCA2*)**

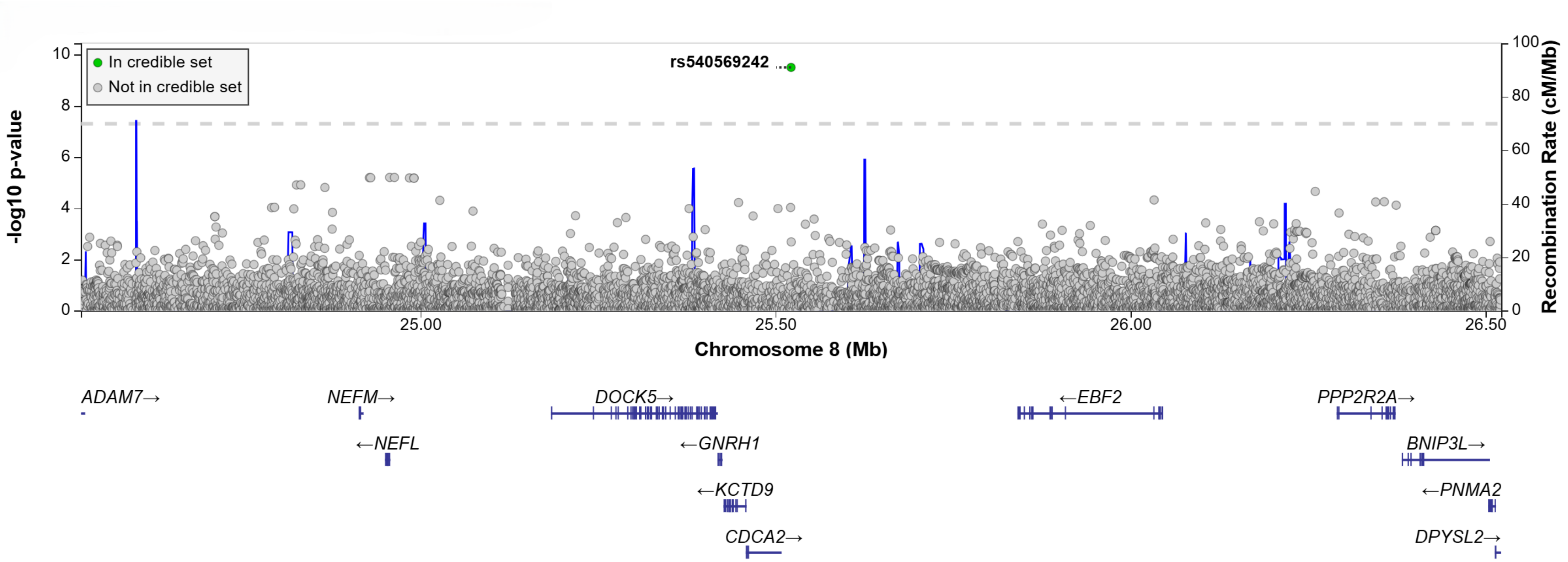

Supplementary Figure 45: Regional association plot with credible causal variants for novel region **9p24-p23 rs768719522 (*PTPRD*)**

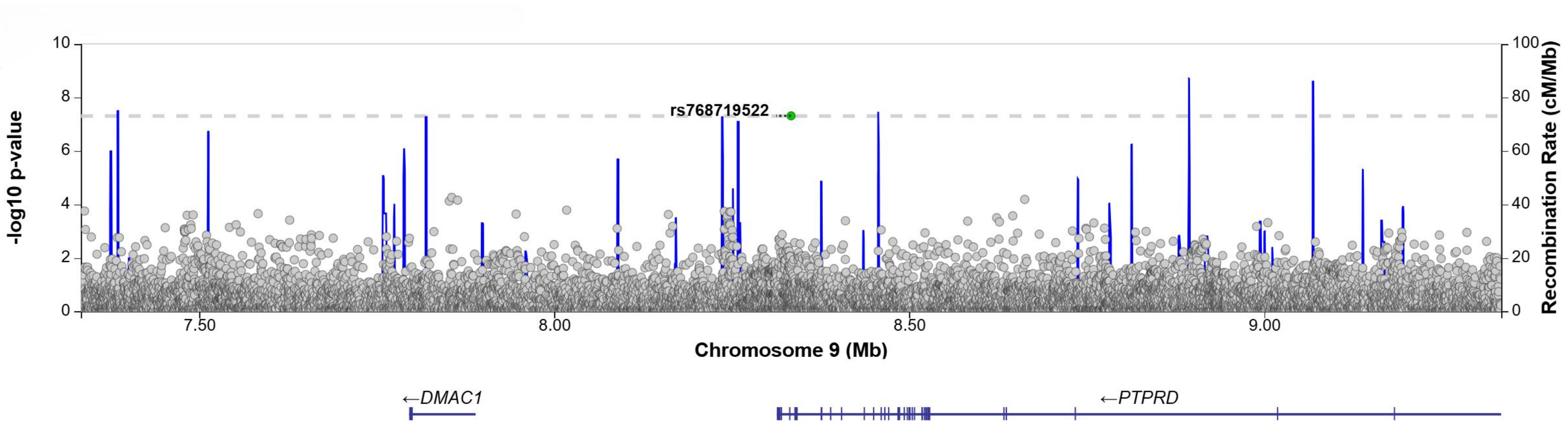

Supplementary Figure 46: Regional association plot with credible causal variants for novel region **16q22 rs6979 (*ACD*)**

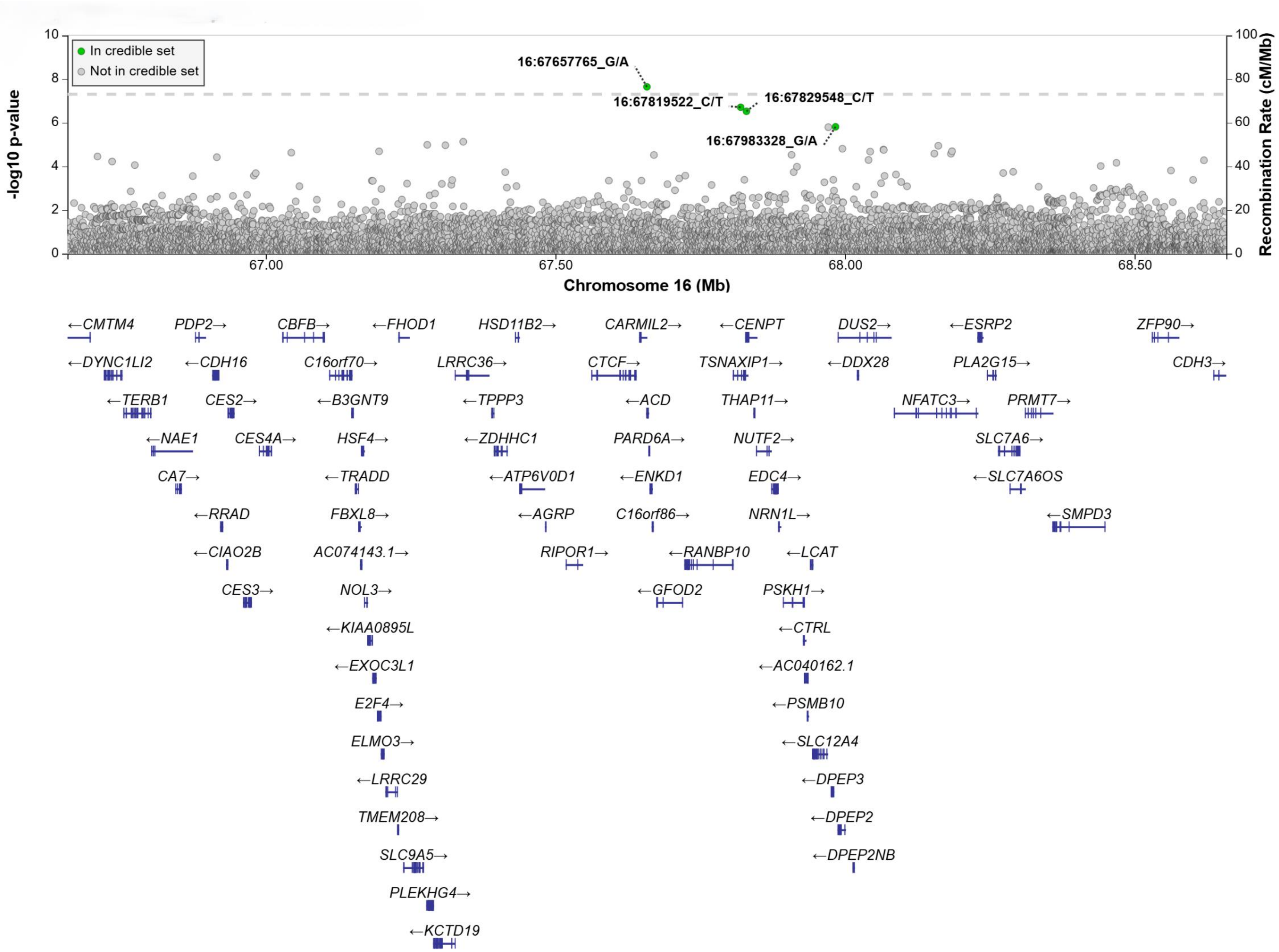

Supplementary Figure 47: Regional association plot with credible causal variants for novel region **17p13 rs78378222 (*TP53*)** and **rs143094271 (*TNFSF12-TNFSF13* / *TNFSF13*)**

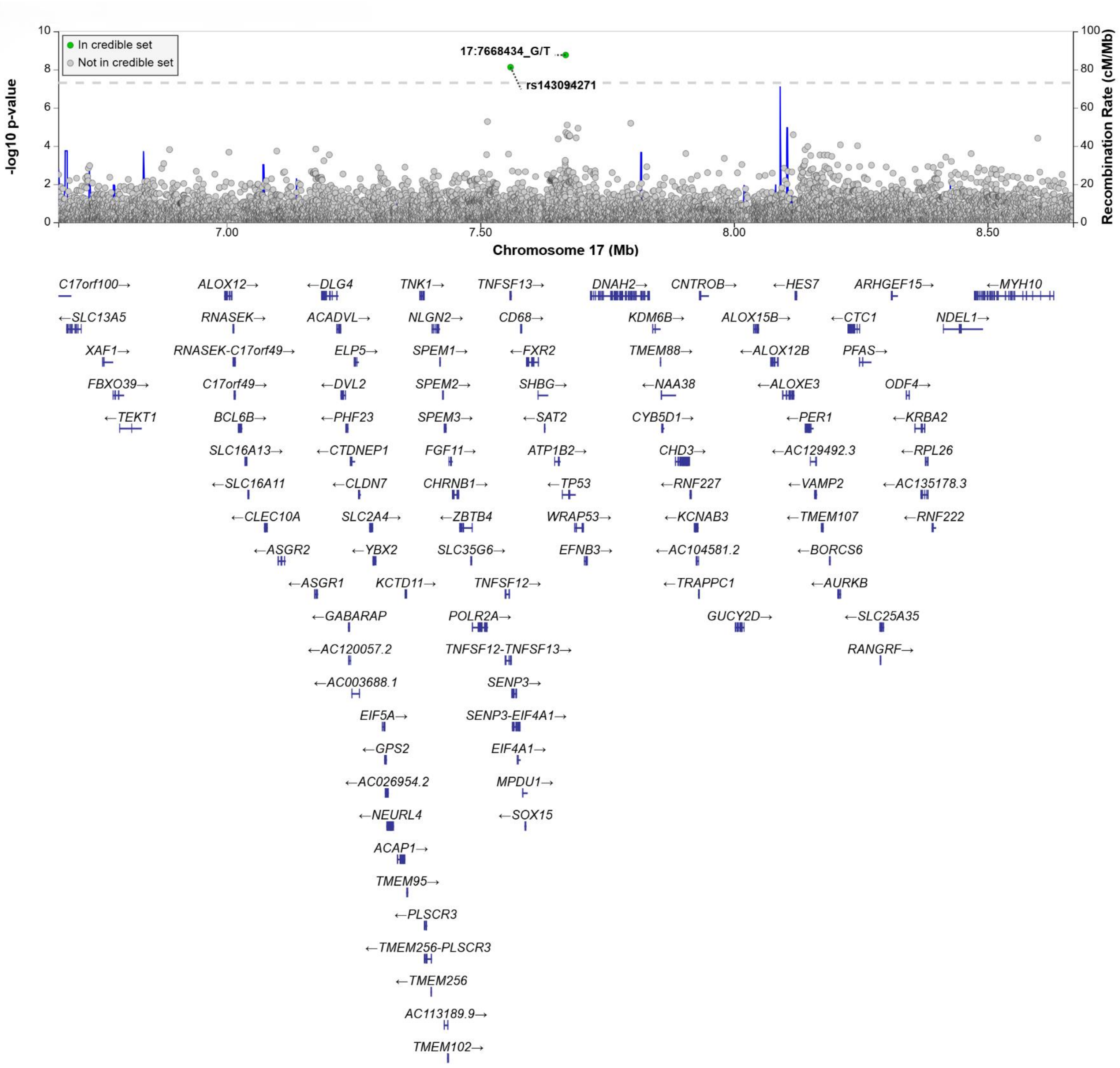

Supplementary Figure 48: Regional association plot with credible causal variants for novel region **19q12 rs62107113 (near *CCNE1*)**

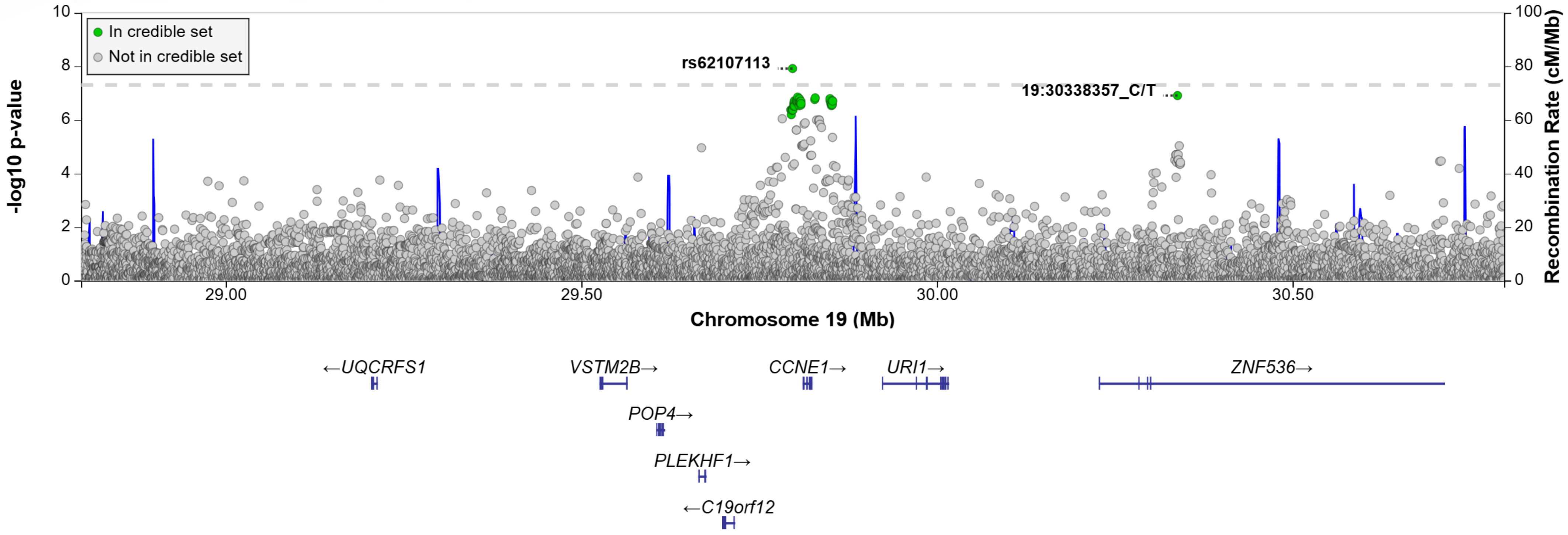

Supplementary Figure 49

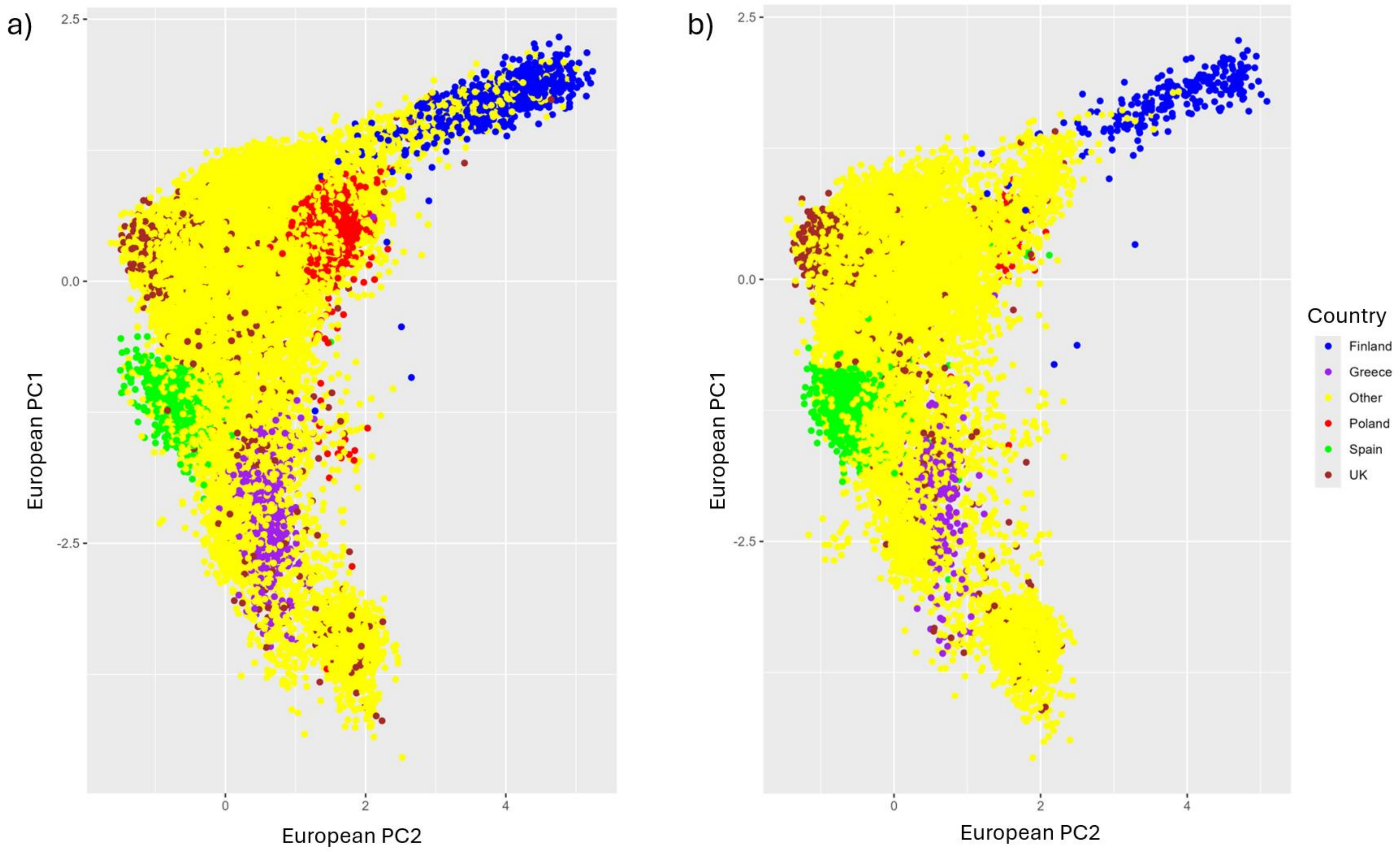

Supplement: Supplementary file 1 — Supplementary Information [file 41525_2025_529_MOESM1_ESM.pdf]
